# Deciphering mechanical cues in the microenvironment: from non-malignant settings to tumor progression

**DOI:** 10.1186/s40364-025-00727-9

**Published:** 2025-01-23

**Authors:** Yicheng Zhu, Jiaoshun Chen, Chen Chen, Rong Tang, Jin Xu, Si Shi, Xianjun Yu

**Affiliations:** 1 Department of Pancreatic Surgery, Fudan University Shanghai Cancer Center, Shanghai, 200032 China; 2 Department of Oncology, Shanghai Medical College, Fudan University, Shanghai, 200032 China; 3 Shanghai Pancreatic Cancer Institute, Shanghai, 200032 China; 4 Shanghai Key Laboratory of Precision Medicine for Pancreatic Cancer, Shanghai, 200032 China; 5 Pancreatic Cancer Institute, Fudan University, Shanghai, 200032 China

**Keywords:** Tumor microenvironment, Mechanical cues, Cellular mechanotransduction, Mechanosensor, Cancer therapy

## Abstract

The tumor microenvironment functions as a dynamic and intricate ecosystem, comprising a diverse array of cellular and non-cellular components that precisely orchestrate pivotal tumor behaviors, including invasion, metastasis, and drug resistance. While unraveling the intricate interplay between the tumor microenvironment and tumor behaviors represents a tremendous challenge, recent research illuminates a crucial biological phenomenon known as cellular mechanotransduction. Within the microenvironment, mechanical cues like tensile stress, shear stress, and stiffness play a pivotal role by activating mechanosensitive effectors such as PIEZO proteins, integrins, and Yes-associated protein. This activation initiates cascades of intrinsic signaling pathways, effectively linking the physical properties of tissues to their physiological and pathophysiological processes like morphogenesis, regeneration, and immunity. This mechanistic insight offers a novel perspective on how the mechanical cues within the tumor microenvironment impact tumor behaviors. While the intricacies of the mechanical tumor microenvironment are yet to be fully elucidated, it exhibits distinct physical attributes from non-malignant tissues, including elevated solid stresses, interstitial hypertension, augmented matrix stiffness, and enhanced viscoelasticity. These traits exert notable influences on tumor progression and treatment responses, enriching our comprehension of the multifaceted nature of the microenvironment. Through this innovative review, we aim to provide a new lens to decipher the mechanical attributes within the tumor microenvironment from non-malignant contexts, broadening our knowledge on how these factors promote or inhibit tumor behaviors, and thus offering valuable insights to identify potential targets for anti-tumor strategies.

**Supplementary Information:**

The online version contains supplementary material available at 10.1186/s40364-025-00727-9.

## Highlight

Distinct mechanical cues in the tumor microenvironment drive tumor behaviors and highlight potential therapeutic targets.

**Supplementary Information:**

The online version contains supplementary material available at 10.1186/s40364-025-00727-9.

## Background

Cancer, posing a significant global health threat, represents an abnormal form of cell growth histologically. Despite advances in cancer research, mortality rates remain troubling, with cancer ranking as the second most common cause of death in the United States [1]. Over the decades, our understanding of tumors has evolved from viewing them as solely genetic diseases driven by mutations to recognizing a broader framework [2, 3]. It is now understood that genetic alterations alone are insufficient to explain tumor development, progression, immune resistance, and response to treatment.

**Fig. 1:**
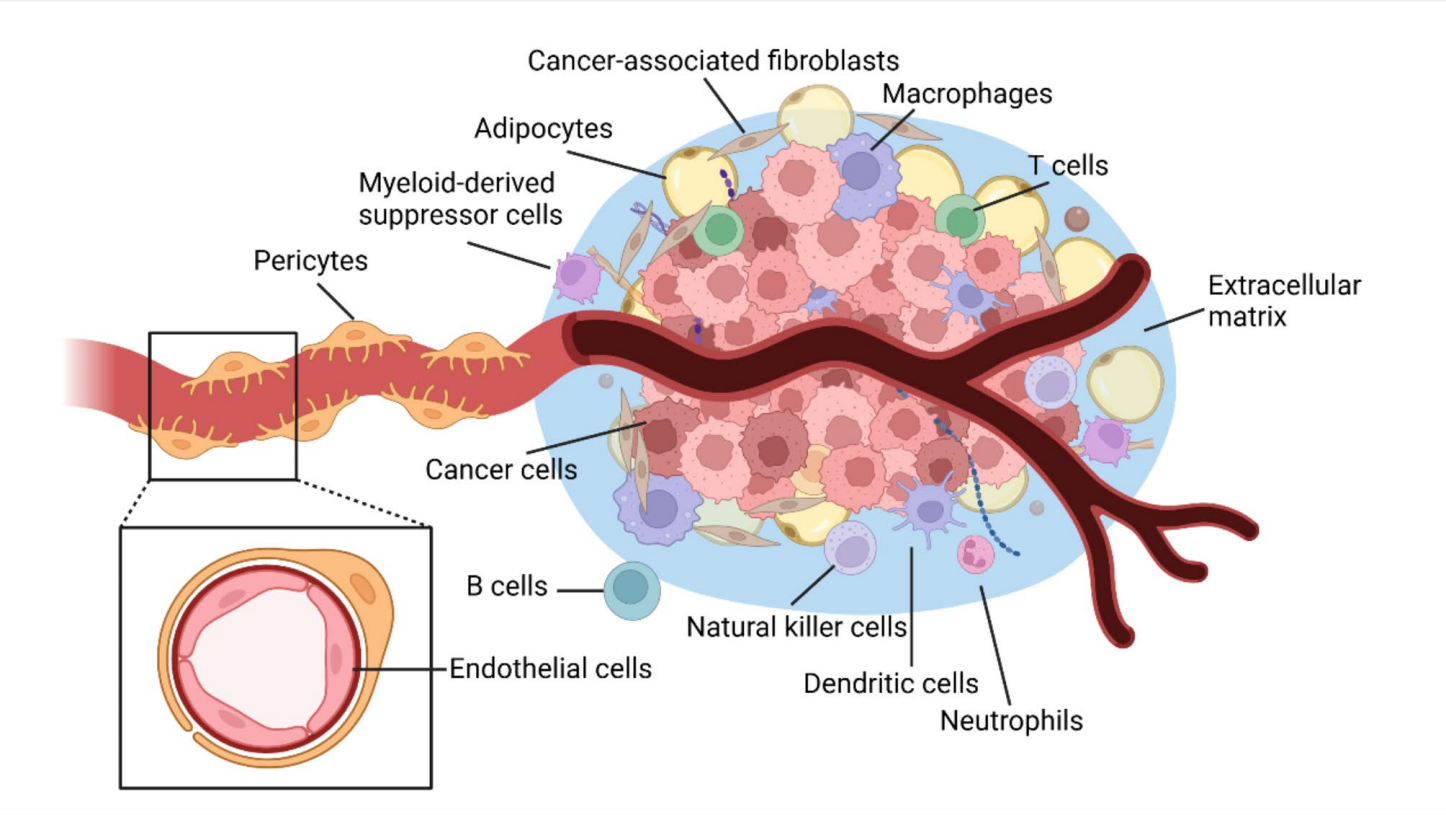
An overview of the tumor microenvironment. The tumor microenvironment is an intricate biosystem consisting of various cellular and non-cellular components, such as cancer cells, cancer-associated fibroblasts, endothelial cells, pericytes, adipocytes, myeloid-derived suppressor cells, macrophages, T cells, B cells, neutrophils, dendritic cells, natural killer cells, extracellular matrix, and blood vessels. This figure was created using Biorender.com

In efforts to unravel the complexities of tumor development and improve treatment outcomes, researchers have turned their attention to the milieu surrounding cancer cells. This focus includes investigating the intricate interactions among cancer cells, neighboring non-cancerous cells, blood vessels, and other elements within the tumor microenvironment (TME; Fig. 1) [4]. The TME concept represents a shift towards viewing tumors as more than just collections of cancer cells. They are now seen as intricate ecosystems where each component plays a distinct role in tumor behavior and characteristics such as invasiveness and metastasis [5]. Apart from cancer cells, this microenvironment consists of various non-cancerous cells, including cancer-associated fibroblasts (CAFs) [6], endothelial cells (ECs) [7], pericytes [8], adipocytes [9], neurons [10], myeloid-derived suppressor cells (MDSCs) [11], a variety of immune and inflammatory cells (i.e., macrophages, CD8^+^ T cells [12–15], CD4^+^ T cells [16–18], regulatory T cells (Tregs) [19, 20], B cells [21], neutrophils [22], dendritic cells (DCs) [23] and natural killer (NK) cells [24]), and structural components like the extracellular matrix (ECM) [25]. While this diverse mix of cells was once considered passive observers, tempering therapeutic effects with heterogeneity [26], they are now recognized as crucial players in tumor progression, offering potential targets for therapeutic intervention [27, 28].

Despite advancements in our understanding of tumor biology and treatments, gaps persist in effectively managing aggressive cancers in the pancreas, liver, esophagus, and lungs [1]. For instance, pancreatic ductal adenocarcinoma (PDAC) is a lethal disease with poor responsiveness to conventional chemotherapy [29]. Research has noted the correlation between chemoresistance and the dense desmoplastic ECM around the tumor [30], yielding a stiff mechanical environment for cells within TME. This suggests the existence of possible novel pathways through which mechanical stimuli impact cancer cells and other components in the TME, potentially influencing key tumor behaviors including invasion, metastasis, and drug resistance. This underlying biomechanical-biochemical interplay begins with a sensor-mediated cellular translation of mechanical cues, leading to fundamental changes such as alterations in protein conformation, protein translocation, or ion channel activity, which ultimately result in specific intracellular biochemical signals. These findings underscore the significance of delving deeper into the mechanical factors at play within the TME. While progress has been made in studying the metabolic and immune factors within tumors [31, 32] (Fig. 1), the interaction between mechanical properties and tumor behaviors remains elusive. This review aims to consolidate our knowledge of the mechanical aspects of both normal cell behavior and the TME, shedding light on how mechanical cues promote or inhibit tumor development and facilitating the exploration of novel anti-cancer treatment strategies.

## Cellular mechanotransduction

In response to external mechanical stimuli, certain cells possess the ability to sense these cues and translate them into biological responses within the cell, influencing cellular functions, gene expression, and epigenetic modifications. This fundamental process, known as cellular mechanotransduction, plays a crucial role in modulating cellular behaviors and is essential in both healthy and diseased states [33]. Successful cellular mechanotransduction relies on three key components: external mechanical cues, sensors for stimuli, and intrinsic signaling pathways. The study of cellular mechanotransduction initially focused on how tissues maintain homeostasis under mechanical loads, later expanding to encompass tissue growth, organ development, and organismal functions [34]. Technological advancements, including tools like atomic force microscopy, optical tweezers, laser ablation, and DNA-based force spectroscopy, have enabled precise quantitative measurements of mechanical forces at varying scales within and between cells and their microenvironments [35] (Table 1). Therefore, the current research indicates that cellular mechanotransduction plays a pivotal role in a range of physiological and pathological processes, influencing embryo morphogenesis [36, 37], immune responses [38], senescence [39], tissue regeneration [40], neural development [41], angiogenesis [42], fibrosis [43], tumor development [25], and drug resistance in cancer treatments [44]. Ongoing studies are delving into the mechanisms behind mechanotransduction’s influence on tissue homeostasis maintenance and associated diseases, aiming to identify potential therapeutic targets.

### Mechanical cues

**Fig. 2:**
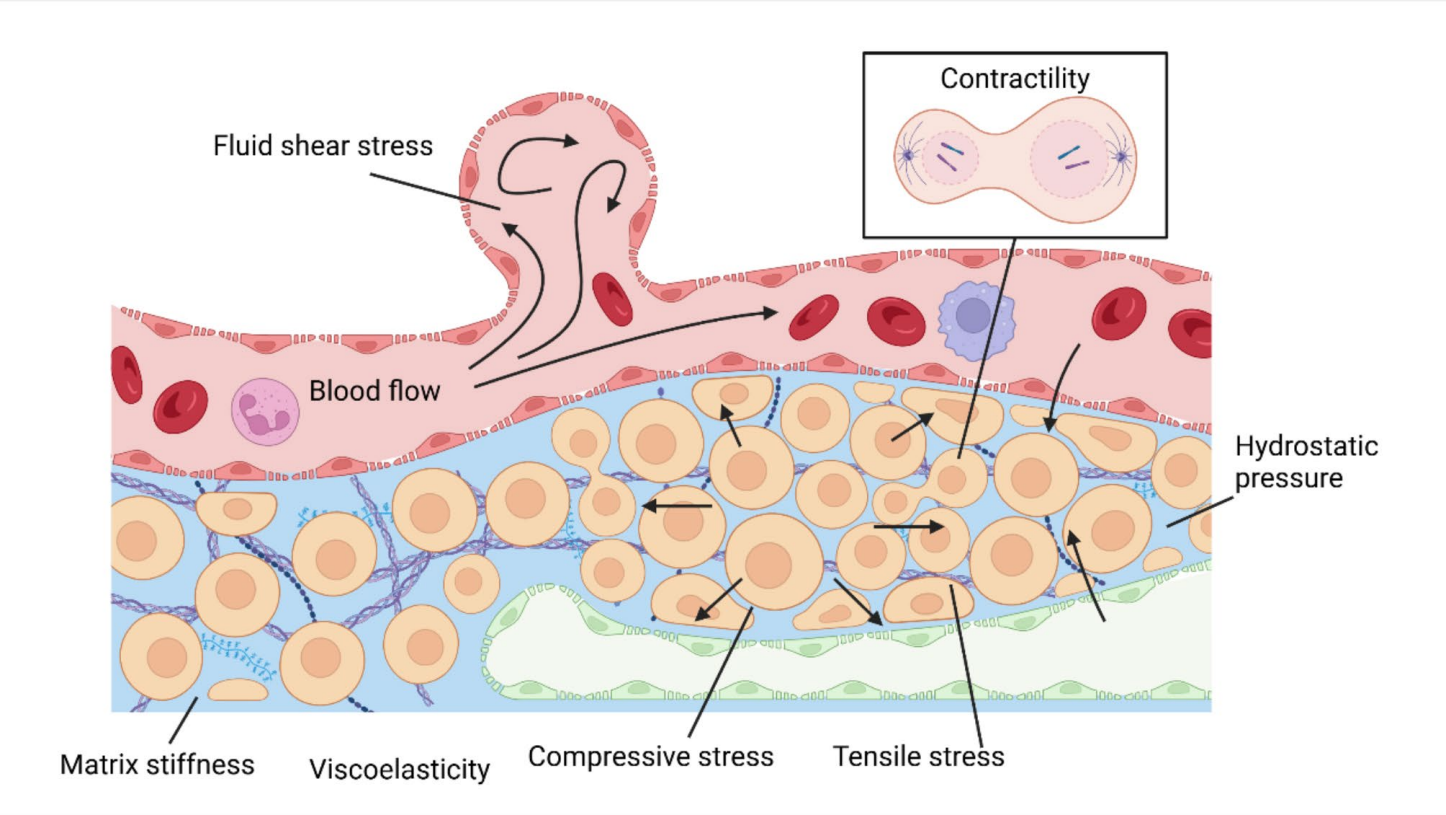
An overview of the mechanical cues. Tensile stress can manifest as membrane tension. Compressive stress can be generated by cell growth and proliferation. Fluid shear stress is induced by blood flow. Hydrostatic pressure is linked to fluid exchange between blood, tissue fluid, and lymph. Matrix stiffness and viscoelasticity are inherent properties. Contractility functions in the cytokinesis. This figure was created using Biorender.com

Mechanically, various physical traits within a living material play a pivotal role in cellular mechanotransduction by generating essential mechanical stimuli. Classical biological mechanical cues involved in this process include tensile stress [45], compressive stress [46], fluid shear stress [47], hydrostatic pressure [48], matrix stiffness [49], viscoelasticity [50], residual stresses [51], and contractility [52] (Fig. 2).

#### Tensile stress and compressive stress

Tensile stress, also known as tension or stretching force, refers to the maximum load a material can withstand without fracturing under stretching. It describes forces that either elongate the material or restore it to its original state when subjected to an external load. Biologically, tensile stress refers to the surface-stretching forces to elongate a living tissue in a direction perpendicular to its surface [45]. For instance, fibroblasts can induce tensile stress by contracting or migrating within a desmoplastic ECM microenvironment [53]. In cancer spheroid models, cancer cells generate actomyosin-mediated contractile forces that deform the ECM, producing tensile radial forces within the matrix [54]. At a cellular level, membrane tension is a distinct mechanical feature, representing the energy required to expand the membrane area. This tension can be augmented by factors such as peripheral protein binding, transmembrane proteins, and interactions with the actomyosin cortex [55].

In contrast, compressive stress, or compression, refers to the force that compresses a material, shortening or shrinking it in the direction perpendicular to its surface, and reducing its volume under external stresses [56]. Compressive stress often occurs when cells proliferate in dense, confined environments, such as regions with high deposition of ECM components.

Tensile and compressive stresses are typically quantified at the microscale using techniques like atomic force microscopy (AFM) [57, 58], which offers high-resolution imaging at the nanoscale but is costly. Optical tweezers allow for precise force manipulation but have a limited force range [59]. Alternatively, micropipette aspiration provides a straightforward approach for evaluating surface tension, albeit with potential constraints in providing detailed mechanical insights beyond surface properties [60].

#### Residual stresses

Living materials exhibit a remarkable ability to actively modify their microstructure in response to environmental factors, leading to the generation of internal stresses that persist even in the absence of external loads, such as those found in the left ventricular chamber or pericardium [61]. These self-equilibrating internal stresses, known as residual or solid stresses [62], arise from disparities in regional geometries caused by processes such as morphogenesis, development, growth, and remodeling [63]. Residual stresses are mechanical stresses present within, and propagated by, the solid and elastic components of the ECM and cells [64], including both tensile and compressive stresses. These internal stresses can fortify a material’s resistance to externally applied stress in the opposite direction, as the external load must counteract the pre-existing internal stresses. For instance, compressive residual stresses can reinforce tensile properties, while tensile residual stresses can bolster compressive strength in living materials.

Measuring residual stresses in biological tissues is more challenging than assessing stiffness. A proposed partial-cut method enables bulk measurements but fails to capture stress heterogeneity within tumors and is not suitable for small or in situ tumors [65, 66]. Alternatively, methods using fluorescent oil microdroplets, such as fluorescent oil micro-droplet injection [67] and single-molecule fluorescent force sensors [68], are constrained by the limitations of optical measurements and the incompressible nature of oil microdroplets to isotropic forces at cellular and subcellular scales. New approaches integrate experimental and mathematical frameworks tailored to different tumor types. These include planar-cut methods for 2D stress mapping, slicing for metastatic lesions, and needle biopsy for in situ assessment. Residual stresses can be estimated by analyzing stress-induced deformations using imaging techniques such as high-resolution ultrasonography or optical microscopy [69].

#### Fluid shear stress

Shear stress is defined as the force that induces deformation in a material by slippage along a plane or planes parallel to the applied stress [56]. In living materials, fluid shear stress is the most studied type. It arises from frictional forces generated by fluid flow along the luminal surfaces of organ walls or capsules, such as those in the heart, vascular system, urinary system, and respiratory system. Fluid shear stress is typically estimated using the Hagen-Poiseuille formula, which incorporates factors such as volume flow rate, viscosity, and the radius of the lumen [70]. Generally, shear stress increases as the channel narrows or the flow velocity increases. In large blood vessels and arterioles, fluid shear stress typically ranges from approximately 10 to 50 dyn/cm² based on these calculations [71].

Hemodynamics in large vessels are commonly evaluated using Doppler ultrasonography or magnetic resonance imaging (MRI) [72]. While these non-invasive techniques provide valuable functional and anatomical insights, they can be costly and require specialized equipment, typically found in clinical imaging facilities. Other macroscale methods, such as intravital microscopy for real-time vascular dynamics observation [73], ultrasound localization microscopy (ULM) for detailed microvessel quantification [74], and high-resolution micro-CT post-vascular corrosion casting for precise 3D visualization of vascular structures [75], also require significant equipment investment and specialized training. On the microscale, methods like micropipette aspiration and parallel-plate rheometry offer high accuracy in measuring fluid shear stress but are limited in throughput [76, 77].

#### Hydrostatic pressure

Hydrostatic pressure is a prevalent stress in tissues and fluid-filled organs, such as blood vessels, the heart, the eye, the urinary bladder, the liver, and joint cavities. It refers to the pressure exerted by fluid, typically due to gravity, against capillary walls or surrounding tissues. This pressure includes the interstitial fluid pressure (IFP) and plays a vital role in maintaining fluid balance within these structures. For instance, while osmotic pressure retains fluids within capillaries due to solute concentration differentials, capillary hydrostatic pressure pushes fluid out of capillaries [48]. In the interstitial space, typical hydrostatic pressure is around − 4 cmH_2_O [78]. Increased intrahepatic resistance can elevate the pressure gradient between the inferior vena cava and portal vein by more than 5 mmHg, linking liver cirrhosis to portal hypertension. This, in turn, accelerates trans-sinusoidal fluid flux and raises intrahepatic IFP [79, 80].

Multiple techniques have been developed to measure fluid pressures in vivo. Direct methods include traditional needle-based approaches, such as servo-controlled micropipettes and wick catheters, as well as novel transducer-based methods [81, 82]. These direct techniques vary in invasiveness and accuracy, with limitations such as depth restrictions and potential tissue damage, which impede their clinical application. In contrast, non-invasive methods utilize fluid flow measurement or matrix stiffness assessment through imaging modalities like dynamic contrast-enhanced MRI, convection MRI, diffusion-weighted MRI, magnetic resonance elastography (MRE), ultrasound elastography, and innovative approaches using ultrasound contrast agent microbubbles as pressure sensors [82, 83]. These indirect techniques provide valuable insights into IFP in tumors without the need for invasive procedures, offering a non-invasive way to assess the TME and potentially guide treatment strategies.

#### Matrix stiffness

Stiffness, defined as the ratio of force to displacement, is an inherent characteristic within materials, determined by both their intrinsic properties and the geometry of the structure [56]. In living materials, ECM is an essential non-cellular structure, composed of various elements, including collagens, fibronectin, elastin, laminins, proteoglycans, glycosaminoglycans, and other glycoproteins interwoven to form a complex network [84]. ECM exhibits an intrinsic property known as ECM stiffness, also referred to as tissue elasticity or rigidity, which characterizes a tissue’s resistance to deformation under slowly applied force. The stiffness of the matrix is determined by the combined elastic properties of its constituent elements, hence, any alterations or modifications in ECM components can impact the overall stiffness.

Matrix stiffness can be evaluated using various techniques at different scales. At the macroscopic level, methods such as shear wave elastography [85, 86] and MRE [87] provide non-invasive assessments suitable for clinical use, though they may require specialized equipment and training. In research laboratories, AFM can be employed to measure heterogeneous stiffness distributions in vitro with high resolution [45]. However, AFM has limitations in throughput, vertical range, and magnitude. Other techniques, such as parallel-plate rheometry and magnetic tweezers, can also be used to measure in vitro stiffness with a reasonable degree of accuracy [88, 89].

#### Viscoelasticity

Viscoelasticity is a fundamental property of living tissues and organisms, where they exhibit behaviors akin to a spring or rubber ball when subjected to pressure or stretch, deviating from the behavior of a purely elastic solid (i.e., Hookean solid) [90]. This property entails both elastic and viscous responses: an instantaneous elastic reaction typical of elastic solids and a time-dependent mechanical response characterized by energy dissipation, as seen in viscous liquids [91].

Viscoelasticity can be measured using a variety of techniques across different scales. At the macroscopic level, rheometers are commonly utilized, although they may have limitations in spatial resolution. On the nanoscale, AFM is often used to characterize surface properties, although it may not provide detailed information on intracellular properties. Particle tracking microrheology, another nanoscale method, offers insights into intracellular properties but may incur higher costs. For clinical assessments, techniques such as shear wave dispersion ultrasound vibrometry (SDUV) and MRE have proven effective [92].

#### Contractility

Contractile stress, or contractility, refers to the ability of a material to contract independently of both initial preload and afterload. Biologically, the generation of contractile force is primarily mediated by actomyosin. This phenomenon is predominantly observed in the sarcomeres of striated muscles, by which skeletal muscle cells generate movement and cardiomyocytes facilitate circulation. However, contractility also occurs in non-muscle and smooth muscle cells, where the disordered actomyosin network within the cell cortex plays a crucial role in cell shape modifications [92] and cellular movements like cytokinesis [93]. Those actomyosin-based subcellular movements are commonly assessed using traction force microscopy, which provides high-resolution imaging at the single-cell scale but is limited by extremely low throughput and sensitivity to noise [94].

**Table 1:** Standardized measurement techniques for mechanical cues

Scale	Methods	Mechanical Cues	Advantages	Disadvantages	References
Microscale	Atomic force microscopy	Tensile stress, compressive stress, stiffness, viscoelasticity	High accuracy, subcellular resolution	Low throughput, expensive, complicated setup, limited vertical range and magnitude	[45, 57, 58, 91, 95]
	Micropipette aspiration	Membrane tension, shear stress, fluid pressure, viscoelasticity	High accuracy comparable to AFM, rapid and simple	Invasive, low throughput	[60, 77, 82]
	Parallel-plate rheometry	Shear stress, stiffness, viscoelasticity	High accuracy, high reproducibility, simple setup	Low throughput, limited spatial resolution, only whole-cell scale	[76, 88, 95]
	Magnetic tweezers	Stiffness, viscoelasticity	High timing and force accuracy; measuring extracellular and intracellular properties, simple setup	Low throughput, limited force range, unidirectional forces only	[89, 95]
	Optical tweezers	Tensile stress, stiffness, viscoelasticity	Non-invasive, high timing resolution, accurate force resolution	Only whole-cell scale, limited force range	[59, 95]
	Particle tracking microrheology	Viscoelasticity	Measuring intracellular property, non-invasive	Expensive	[91, 95]
	Traction force microscopy	Contractile stress	Single-cell measurements, high resolution	Extremely low throughput, sensitive to noises	[94]
Macroscale	Magnetic resonance imaging (MRI) based methods	Shear stress, stiffness, viscoelasticity	Clinical applications, non-invasive	Expensive, specialized training, potential contrast agent	[72, 82, 87, 91]
	Ultrasound based elastography	Fluid pressure, stiffness	Non-invasive method, fast and cheap	Limited field of view	[82, 85, 86]
	Shear wave dispersion ultrasound vibrometry	Viscoelasticity	Non-invasive, possible clinical applications	Sensitive to noises	[91]
	Doppler ultrasonography	Shear stress	Clinical applications, non-invasive	Limited field of view	[72]
	Rheometer	Viscoelasticity	Most primitive and classical	Limited spatial resolution	[91]

These interwoven mechanical interplays occur between adjacent cells, cell-ECM interactions, as well as adhesion and filament networks, establishing the mechanical context of the cell microenvironment. Spanning from the nanoscale to the tissue level, these interactions significantly influence cellular mechanotransduction processes and subsequent cell responses.

### Mechanosensors

**Fig. 3:**
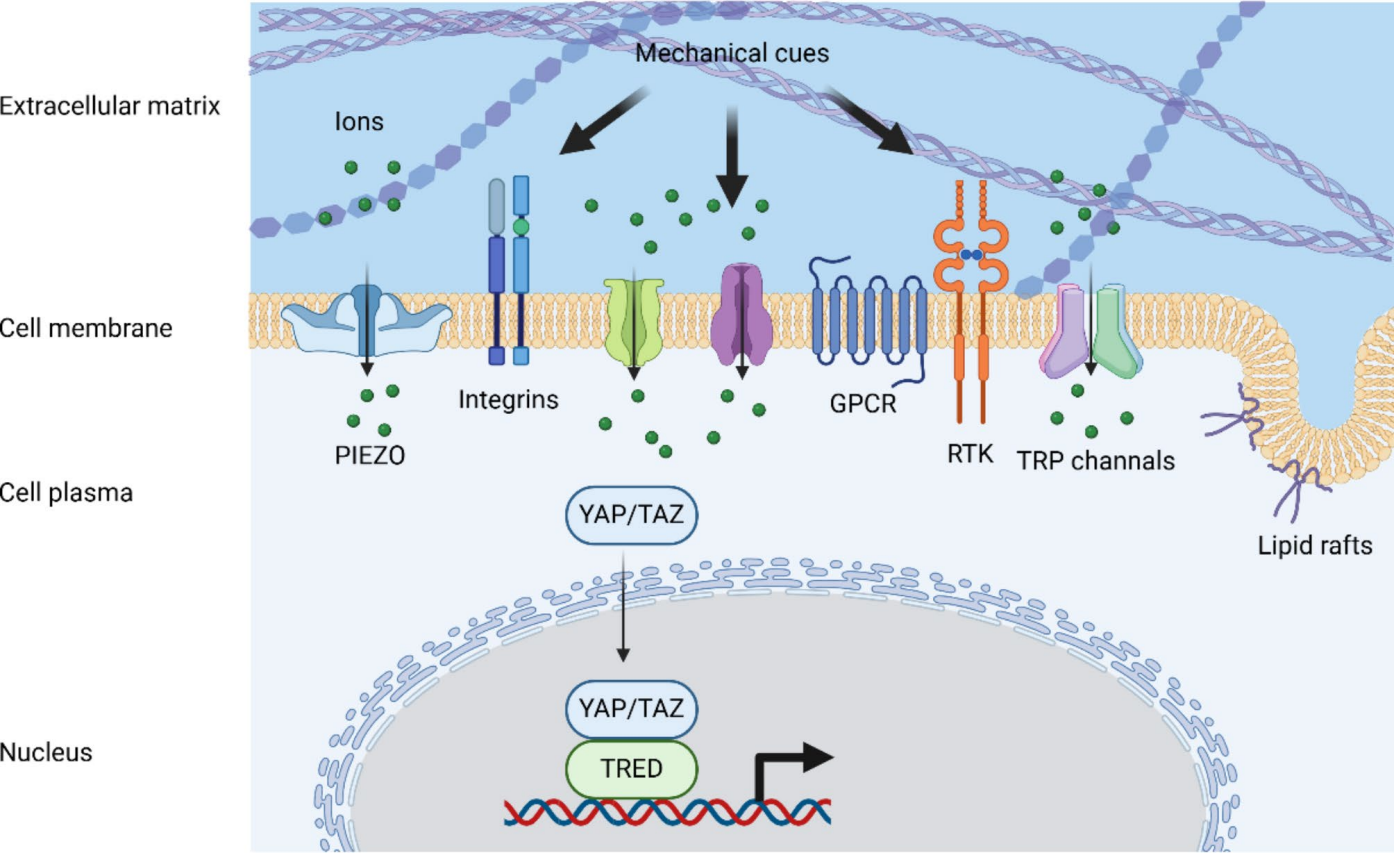
An overview of mechanosensors. The mechanical cues are typically detected through mechanosensors including ion channels (i.e., PIEZO and TRP channels), integrins, lipid rafts, G protein-coupled receptors (GPCRs), receptor tyrosine kinases (RTKs), and transcription factors (i.e., YAP/TAZ). This figure was created using Biorender.com

The mechanical cues outlined earlier are typically detected through mechanical stress-induced conformational or organizational shifts in entities including ion channels [96], integrins [97], cadherin complexes [98], lipid rafts [99], G protein-coupled receptors (GPCRs) [100], receptor tyrosine kinases (RTKs) [101], and transcription factors [102] (Fig. 3). Activation of these sensors initiates signaling cascades that alter cell destiny. However, these sensors exhibit distinct behaviors in mechanotransduction, with some mechanisms remaining inadequately understood. Whether acting independently or in collaboration, these sensors contribute to intricate interactive networks that regulate cellular functions. Their involvement underscores the pivotal role of cellular mechanotransduction in homeostasis and disease pathogenesis, highlighting the potential for therapeutic interventions targeting these pathways to address a wide range of pathological conditions.

#### PIEZO

PIEZO proteins (PIEZO1 and PIEZO2) are classified as mechanosensitive ion channels, exhibiting three kinetic states: open, closed, and inactivated. These channels play a pivotal role in translating mechanical cues into biochemical signals, contributing significantly to established mechanosensory functions like touch [103], mechanical allodynia [104], and the baroreceptor reflex [105].

The significance of PIEZO channels has been underscored in various developmental processes such as lymphatic valve development [106], heart valve development [107], angiogenesis [108], stem cell differentiation [109], and cell division [110], as well as regulatory functions including bone formation [111], cell migration [112], axon regeneration [113], the inflammatory response [38], and red blood cell (RBC) volume regulation [114]. Notably, a mild gain-of-function mutation in PIEZO1 has been linked to RBC dehydration and conferred malaria resistance among Africans [115].

#### Integrins

Integrins are cell surface transmembrane receptors that serve as crucial links between ECM and the cytoskeleton, playing a vital role in cell adhesion and the transmission of biochemical signals [116]. Activation of integrins occurs through intracellular signals that facilitate the binding of proteins (i.e., talin and kindlin) to the β-integrin tail. This binding induces a conformational change in the receptor, enhancing its affinity for ECM ligands [117]. The subsequent interaction between integrins and ECM ligands initiates signaling cascades involving protein complexes of scaffold and adaptor molecules, kinases, and phosphatases to regulate cellular behavior [118].

In mammals, the varied combination of 18 α-subunits and 8 β-subunits leads to the generation of 24 distinct integrin heterodimers, enabling engagement with different ECM ligands and the activation of diverse signaling pathways [119]. Consequently, the expression and activity of integrins on the cell membrane significantly impact the biological response to environmental cues. Integrins are not only crucial for cell-ECM interactions but also act as mechanotransducers in cell-cell communication processes, facilitating numerous physiological and pathophysiological processes including cell adhesion, proliferation, differentiation, spreading, and migration [120, 121]. Beyond common mechanical forces, integrins can also be activated by magnetic force, influencing osteocyte fate through an ECM-integrin-C terminal Src kinase (CSK) axis [122].

#### YAP/TAZ

Yes-associated protein (YAP) and transcriptional coactivator with PDZ-binding motif (TAZ) are essential transcriptional co-activators that interpret a diverse array of mechanical stimuli ranging from shear stress to cell shape, density, ECM stiffness, and convert them into specific transcriptional programs by nuclear translocation [123]. These proteins serve as key regulators of the Hippo pathway [124], orchestrating various biological processes including tissue development [42, 125], regeneration [40, 126], angiogenesis [42], cell migration, proliferation [127], cell renewal, differentiation [128], and cellular homeostasis [129–131].

#### Lipid rafts

Lipid rafts are specialized microdomains in the cell membrane, composed of cholesterol, sphingolipids, and specific proteins [132]. These rafts not only serve as platforms for cellular signaling and membrane trafficking, but also function as mechanosensors, responding to mechanical stresses such as blood flow, touch, and hearing. For instance, lipid rafts can detect stress-induced deformation in focal adhesions [99] and initiate downstream signaling pathways, such as the activation of phospholipase D2, which generates phosphatidic acid [133]. They also play a critical role in integrin activation through the translocation of integrin α5 to lipid rafts, a process essential for EC responses to shear stress [134]. Caveolae, a distinct type of lipid raft, can help buffer mechanical stress and provide mechanoprotection [135]. Furthermore, intact lipid rafts play a key role in responding to shear or compressive stress by triggering the phosphorylation of p38 mitogen-activated protein kinases (MAPK), a process that promotes pro-survival autophagy in cancer cells [136, 137].

#### Transient receptor potential channels

Transient receptor potential (TRP) channels belong to a family characterized by a canonical tetrameric structure, participating in a spectrum of sensory functionalities encompassing chemosensation, thermosensation, mechanosensation, and osmosensation [138, 139]. These channels serve a critical role through the sensing and conversion of various stimuli, including nociceptive and thermal cues [138]. Notably, TRP channels play an integral part in multiple disorders such as neuroinflammation [140], inflammatory bowel diseases (IBD) [141], and dry eye disease [142], while also contributing to essential physiological functions like cell migration [143] and osmoregulation [144].

#### Other ion channels

Prokaryotic channels (i.e., mechanosensitive channel large conductance (MscL), mechanosensitive channel small conductance (MscS), and their homologs), along with mechanosensitive channels from the two-pore potassium (K2P) and hyperosmolality-gated calcium-permeable (OSCA/TMEM63) channel families, have been validated as authentic mechanically activated ion channels [139]. Additionally, there are channels like the degenerin/epithelial sodium channel (DEG) family that also contribute to mechanosensation processes [145].

**MscL**,** MscS**,** and MscS-like Channels** were initially identified as mechanosensitive ion channels, and are capable of detecting membrane tension and responding to osmotic changes by allowing the passage of ions and osmolytes, thereby safeguarding cells against lysis [146].

**Two-pore Potassium Channels** including TREK-1, TREK-2, and TRAAK are present in sensory neurons. While these channels do not participate in action potential generation, they can modulate transduction currents and reduce sensitivity to mechanical stimuli by inducing cellular hyperpolarization [147].

**OSCA/TMEM63 Proteins** form a substantial family of mechanosensitive channels, exhibiting stretch activation at a high threshold compared to PIEZO channels [148]. They are crucial factors in detecting osmolarity, food texture [149, 150], audition [151], mitochondrial morphology [152], and myelination [153]. Structurally similar, the transmembrane-like channels (TMCs) also hold a significant position in the auditory system, highlighting their involvement in sensory perception and cellular responses to mechanical stimuli [154].

## Mechanical attributes in non-malignant biological behaviors

In the biological context, mechanical stimuli (i.e., tensile stress, compressive stress, shear stress, hydrostatic pressure, matrix stiffness, and viscoelasticity) are omnipresent within the microenvironment. When exposed to these mechanical cues, tissues and cells undergo intricate cellular mechanotransduction processes that convert physical stresses into biochemical signals. This activation of downstream pathways initiates cascades of biological behaviors, thus influencing numerous physiological or pathophysiological processes (Table 2). Fortunately, mechanical cues and cellular mechanotransduction processes are not exclusive to tumors. By studying shared features from non-malignant biological behaviors, valuable insights can be garnered into the common underlying principles governing cellular behavior in both health and disease.

### Tensile stress

Tensile stress plays a significant role in various biological processes, including muscle movement [155], embryo morphogenesis [36], angiogenesis [156], tissue development [157, 158], and immune responses [159]. The intricate interplay of tensile stress across different scales, from organ function to cellular behavior, underscores its immense importance in maintaining homeostasis and regulating essential mechanisms within the human body.

In the locomotor system, tensile stress is integral to the creation of tension-compression asymmetry in skeletal muscles, influencing muscle fiber volume changes during deformation [155]. Dynamic tensile stress, generated during warm-up exercises as opposed to static stretching, has been observed to affect muscular temperature and post-activation potentiation, contributing to an increased range of motion, enhanced muscle power, and improved muscular performance [160]. Additionally, tensile stress is crucial for voice production through laryngeal muscles, with dysfunction leading to conditions such as muscle tension dysphonia [161].

In the cardiovascular system, tensile stress is generated by blood flow and detected by ECs, smooth muscle cells (SMCs), and cardiomyocytes, triggering various downstream effects. Numerous candidates regulate stretch-induced gene expression in cardiomyocytes, such as nuclear factor-like 2 (Nrf2), interferon regulatory transcription factors (IRFs), and the let-7 family of miRNAs. For instance, tensile stress can induce cardiomyocyte hypertrophy through activation of Nrf2-mediated oxidative stress pathways and inhibition of IRF3/7-mediated ERK1/2 and nuclear factor κB (NF-κB) signaling [162]. Tension heterogeneity among developing cardiomyocytes coordinates cellular behavior via constitutively active myosin light chain 9, reshaping the cardiac chamber wall, inducing cardiomyocyte delamination, and navigating cell fate during cardiac trabeculation [163, 164]. In angiogenesis, tensile stress plays a key role in vascular remodeling and morphogenesis, facilitated by the stretch-responsive secretion of angiogenic factors like angiotensin II and platelet-derived growth factor (PDGF) [156]. Significantly, the integration of tensile stress and electrochemical signaling in cardiomyocytes is critical for ectopic automaticity, electro-anatomical remodeling, and atrial arrhythmogenesis [165].

Tensile stress also impacts neural development, upregulating gene expression such as glycerol-3-phosphate acyltransferase 3 (GPAT3) and small proline-rich protein 1B (SPRR1B), and downregulating neuronal regeneration-related protein (NREP) [157]. PIEZO channels, activated by membrane tension, inherently inhibit axon regeneration via the calcium/calmodulin-dependent protein kinase II (CaMKII)-nitric oxide synthase (NOS)-protein kinase G (PKG) pathway [113]. Additionally, the stability of cadherin-based adherens junctions is tension-dependent, influencing radial glial apicobasal polarity and neuroepithelial cell cohesion [166]. Notably, tensile stress maintains the stemness of neural stem cells through engineered mechanoresponsive RTKs in an adhesion- or endocytosis-mediated mechanism [167]. In wound healing and scarring, Engrailed-1 activation is driven by tensile stress via YAP-mediated mechanotransduction signaling [168]. In the urothelium, tensile stress is mediated through integrin α6-focal adhesion kinase (FAK) signaling, PIEZO channels, and TRP channels, affecting processes such as proliferation and the urethral micturition reflex [158, 169]. Furthermore, organ distension generates tensile stress [165], as seen in the Drosophila crop, where mechanical signals activate Piezo^+^ neurons to transmit satiety signals along the “brain-gut axis” [170].

At the microscale, tensile stress plays a crucial role in cellular deformation. The activation of PIEZO1 in response to local membrane tension is vital for the recruitment of lymphocyte function-associated antigen-1 (LFA1, also known as CD11a/CD18), during T cell chemotaxis [171]. Intriguingly, elevated membrane tensile stress within immunological synapses has been found to significantly augment pore formation by perforin, underscoring the importance of tensile stress in immunity [159]. In human embryo morphogenesis, heightened surface tension at the cell-medium interface is pivotal for processes like compaction, where cells closely interact through factors such as myosin heavy chain 9 (MYH9) and MYH10 [36].

Mechanically, within a matrix resistant to cell tractional forces, the cytoskeleton adapts in a tension-dependent manner, redistributing specific mRNAs and ribosomes to focal adhesion complexes, thereby influencing post-transcriptional regulation of gene expression. For instance, in response to acute ventricular wall stress, there is a rapid escalation in protein synthesis and phosphorylation of translation initiation factor eIF-4E in cardiocytes, a process mitigated by inhibitors of actomyosin-based tension generation [172]. Furthermore, stretching-induced activation of the ataxia telangiectasia mutated (ATM) pathway boosts the nuclear presence of YAP, modulates the actin-related protein 2/3 (ARP2/3) complex, and triggers Kap1 phosphorylation, resulting in cytoskeleton and chromatin remodeling [173]. The nuclear envelope plays a critical role in cellular mechanotransduction, detecting tensile stress and impacting various biological processes, including nuclear migration [174], cell polarization [175], nuclear deformation [176], mitotic entry [177], and chromatin reorganization [176]. For instance, PIEZO1 activation triggers calcium release in response to stretching, leading to the loss of nuclear lamina-associated heterochromatin, thus maintaining genome integrity under nuclear deformation [178].

### Fluid shear stress

Despite its relatively low magnitude under physiological conditions, fluid shear stress plays a crucial role in organ development and tissue homeostasis. Various cell types, including vascular ECs, epithelial cells, osteocytes, chondrocytes, and inner ear hair cells, can perceive shear stress. Among these, blood flow-induced shear stress and its effects on ECs are the most extensively studied [47]. Vascular ECs at the blood-vascular interface respond to shear stress by initiating complex intracellular signaling cascades, involving transcription factors such as Krüppel-like factor 2 (KLF2), KLF4, and N-myc downstream-regulated gene 1 (NDRG1). These cascades modulate vasodilation through endothelial NOS, and regulate cell proliferation and angiogenesis via mediators like vascular endothelial growth factors (VEGFs), resulting in either atheroprotective or atheroprone vascular phenotypes [179, 180].

In embryonic development, fluid shear stress is crucial for PIEZO1-dependent EC alignment and elongation, supporting yolk sac vascular remodeling and vasculogenesis [181]. Notably, the flow patterns within the lumen play a critical role in determining shear stress. In the human vasculature, common regions where typical shear stress is observed include vessel bifurcations, areas of stenosis, aortic aneurysms, heart valves, and capillary networks [183]. Two predominant blood flow patterns are observed: high shear stress (laminar or pulsatile shear) prevalent in straight artery segments and low shear stress (oscillatory or disturbed shear) noted at branching points with abrupt changes in flow direction [183, 184]. Laminar shear stress is generally considered atheroprotective, promoting cell alignment via peroxisome proliferator gamma coactivator-1α (PGC1α) without triggering cell cycle progression, enhancing EC survival while inhibiting coagulation, leukocyte diapedesis, and SMC proliferation [184, 185]. Specifically, high-mobility group box 1 (HMGB1) nuclear translocation in response to laminar flow exerts anti-inflammatory, anti-adhesive, and anti-thrombotic effects [186–187]. Furthermore, pulsatile blood flow induces mechanical strains that can downregulate YAP/TAZ in vascular SMCs, activating the cGAS-STING pathway and inducing a senescence-associated secretory phenotype (SASP) [39]. In contrast, oscillatory shear stress can stimulate endothelial DNA synthesis without inducing cell alignment or discernible cell retraction, promoting EC turnover in atheroprone regions [189]. Persistent oscillatory shear stress can enhance pro-inflammatory responses and atherogenesis through nuclear translocation of BTB and CNC homology 1 (BACH1) and downregulation of uncoupling protein 2 (UCP2) [190–192]. Turbulent flow induces integrin conformational activation, which plays a pivotal role in vascular inflammation by regulating endothelial connexin hemichannel gating [193]. Additionally, disturbed shear stress upregulates pro-oxidant and pro-inflammatory agents, such as activating protein-1 (AP-1) and NF-κB, and hypercholesterolemic agents like proprotein convertase subtilisin/kexin type 9 (PCSK9) [184], triggering an intricate interplay between shear stress and atheroprone responses. Beyond ECs, shear stress affects endothelial adhesiveness for monocytes by enhancing nitric oxide release over prostacyclin at early time points [194], subsequently decreasing endothelial superoxide production, vascular cell adhesion molecule 1 (VCAM1) expression, and monocyte binding [186].

Fluid shear stress is not confined to the cardiovascular system but is also observed in the urinary and respiratory systems. In the kidneys, physiological urinary fluid flow generates shear stress, which regulates renal functions such as proximal reabsorption. Whereas dysregulation of shear stress in the urinary system is linked to various kidney diseases [195]. Primary cilium-dependent autophagy enables kidney epithelial cells to adjust their metabolism in response to fluid flow [196], governed by the AMP-activated protein kinase (AMPK)-sirtuin 1 (SIRT1)-YAP axis, which varies with flow intensity [195]. Furthermore, shear stress from filtrate flow through the glomerular barrier can physically detach podocytes from the glomerular basement membrane, impacting renal function [197]. In the lungs, airflow induces fluid shear stress in the bronchial cross-sectional area, where the TRPV2 channel modulates shear stress-induced ATP release from bronchial epithelial cells [198].

### Hydrostatic pressure

In a healthy physiological state, normal hydrostatic pressure contributes to various biological processes, including osteogenesis [199], neural development [200], immune responses [38], and the regulation of mitosis on a cellular level [201]. This highlights the significance of hydrostatic pressure in maintaining homeostasis and supporting essential cellular functions.

Research has illuminated the bone formation-promoting effects of periodic hydrostatic pressure by upregulating osteogenic genes, such as osteonectin and osteopontin [199]. Additionally, physiological cyclic hydrostatic pressure has been found to stimulate a positive, though variable, commitment of human bone marrow stem cells toward the osteogenic lineage in a cyclo-oxygenase 2 (COX2)-dependent manner [202]. Conversely, hydrostatic pressure has been demonstrated to impair neural crest induction by suppressing YAP activation and diminishing Wnt signaling [200]. During mitosis, animal cells undergo a spherical transformation driven by osmotic pressure, which generates intracellular hydrostatic pressure when the intracellular osmolarity exceeds that of the external environment. This fluid influx influences cell shape, movement, and mitotic mechanics in coordination with local actomyosin cortical tension [201].

Beyond its physiological roles, hydrostatic pressure is also implicated in various pathophysiological processes. Cyclic hydrostatic pressure has been observed to activate PIEZO1 in immune cells, resulting in AP-1 activation and the transcription of endothelin-1 (EDN1), which stabilizes hypoxia-inducible factor 1α (HIF1α) and prolongs pro-inflammatory gene expression. In contrast, PIEZO1 deficiencies impair inflammation in scenarios such as bacterial infections or fibrotic autoinflammation [38]. Deviations in hydrostatic pressure levels can lead to cancellous bone devitalization via Caspase 3-mediated apoptosis, resulting in decompensated lesions. However, maintaining biomechanical stability and specific molecular cues may promote cell differentiation [203, 204]. Moreover, elevated hydrostatic pressure has been linked to atrial electrophysiological remodeling and inflammatory responses, by dampening the L-type calcium current, enhancing the transient outward and ultra-rapid delayed rectifier potassium currents, and upregulating tumor necrosis factor-α (TNF-α) and macrophage migration inhibitory factor (MIF), all of which can increase susceptibility to atrial fibrillation [205].

### Matrix stiffness

Stiffness is a fundamental characteristic of the ECM, intricately linked to the deposition and organization of ECM elements. Fibrillar proteins like collagens and elastic fibers, including elastin, exhibit distinct tensile properties and elasticity, which are primary determinants of ECM stiffness [206]. The organization and composition of these structural components are pivotal in defining ECM’s mechanical properties, impacting cellular behavior and tissue functionality. Additionally, post-translational modifications of ECM components, such as nonenzymatic glycation and collagen crosslinking, and pathological remodeling, can contribute to matrix stiffening [50, 207].

ECM stiffness influences cellular processes through mechanotransduction, with effectors like integrins in fibroblasts sensing stiffness signals and initiating downstream signaling pathways. These cascades are essential for numerous physiological and pathophysiological processes, including cell adhesion, proliferation, migration [121, 171, 208], differentiation [49, 209], neural development [210], neural protection [211], angiogenesis [212], and immune responses [213].

Recent studies have elucidated the impact of ECM stiffness on cellular behavior and tissue-specific differentiation. A stiff ECM environment triggers integrin-dependent activation of glycogen synthase kinase 3 (GSK3) and Src, promoting β-catenin degradation and inhibiting differentiation in human embryonic stem cells. In contrast, in a compliant matrix, β-catenin accumulates at cell-cell adhesions, enhancing Wnt-dependent mesoderm differentiation [214].

In a skeletal progenitor model, an intricate interplay was observed among various matrix-associated bone morphogenetic proteins (BMPs, i.e., BMP 2, 4, 7, and 9) and corresponding receptors, modulating cellular processes such as cell spreading, adhesion, migration, and differentiation in a stiffness-dependent manner [215]. Elevated ECM stiffness has also been implicated in osteoclastogenesis via impeding the integrin β3-mediated ras homolog family member A (RhoA)-Rho-associated coiled-coil containing protein kinase 2 (ROCK2)-YAP mechanotransduction pathway [49]. Intriguingly, curved ECM fibers, rather than straight ones, have been shown to enhance cell bridge formation, facilitating cell proliferation and osteogenic differentiation [216].

In a Xenopus laevis neural crest cell model, augmented matrix stiffness stimulates epithelial-to-mesenchymal transition (EMT), triggering collective cell migration critical for morphogenesis, tissue remodeling, and cancer invasion [217]. Extensive research has elucidated the morphogenetic role of ECM on neural development, particularly synaptic plasticity and regeneration [218, 219]. Neurons typically thrive in compliant environments, while glial cells exhibit a preference for stiffer substrates [220, 221]. Mechanically, PIEZO1 can trigger calcium influx and YAP nuclear translocation in a stiffness-dependent manner, navigating neurogenesis while hindering astrogenesis in human neural stem cells [109]. Moreover, microglial PIEZO1 has been identified as a critical sensor for the stiffness of extracellular amyloid-β fibril, triggering phagocytosis to restrict Alzheimer’s disease progression [211].

Recent investigations have unveiled the stiffness-responsive phenotypic shifts of human tendon stromal cells, modulated by transcriptional programs linked to chromatin remodeling and Hippo signaling, while compliant matrices support cell stemness, synapse formation, and angiogenesis [212]. In skin organoids, a stiff microenvironment activates YAP, leading to the induction of Wnts and matrix metalloproteinases (MMPs), which facilitate cellular movements and promote epidermal cell protrusion, enabling stem cell self-organization [222].

Matrix stiffness also regulates the release and activation of transforming growth factor (TGF)-β [223], a crucial modulator of various pathophysiological processes such as wound healing, fibrosis, immune responses, cancer progression, and metabolic disorders [213]. For instance, integrin αvβ8-mediated TGF-β activation by epithelial cells, DCs, and fibroblasts modulates T cell behavior, maintaining T cell populations in circulation and tissues. Notably, targeting TGF-β activation in Tregs and DCs can evoke either immune-enhancing or immune-suppressing responses [213].

Abnormal tissue stiffness is implicated in various non-cancerous diseases, including multiple organ fibrosis [43], vascular smooth muscle disorders [224], and benign pancreatic diseases [225]. Excessive collagen synthesis and accumulation, particularly collagen types I and III, contribute to myocardial fibrosis and the progression of cardiac dysfunction in hypertensive heart disease [226–229]. In patients with heart failure with preserved ejection fraction (HFpEF), a pronounced increase in passive myocardial stiffness is observed, which depends on collagen and titin, underscoring the pivotal role of ECM homeostasis in cardiac failure progression [230]. ECM stiffness is also associated with aortic aneurysm pathogenesis, where it sustains vascular SMC mechanosensation via Netrin 1-induced PIEZO1 upregulation [231]. Chronic hepatic insults, such as hepatitis B, hepatitis C, alcoholic hepatitis, or bile duct obstructions, trigger mechanical stresses that activate hepatic stellate cells (HSCs), accelerating ECM deposition. This results in matrix stiffening through collagen crosslinking by lysyl oxidases (LOX), transglutaminases (TG), or nonenzymatic glycation, fostering fibroblast-myofibroblast transformation and liver fibrosis [232, 233]. Moreover, a feedback loop is noted where cellular senescence and increased ECM stiffness boost SRY-box transcription factor 9 (SOX9) expression, fueling further ECM alterations that amplify stiffening and senescence, further modulating vascular SMC phenotypes [224].

### Viscoelasticity

Viscoelasticity is a prevalent trait across mammals, observed in both soft and stiff tissues. Soft tissues such as the liver, breast, muscle, skin, and adipose tissue [234–237], exhibit viscoelastic properties, as do stiffer skeletal tissues like bone, tendon, ligaments, and cartilage [238–242]. Regenerative structures such as fracture hematomas [243] and blood clots [244], also demonstrate viscoelastic behavior. The brain, as one of the softest and most dissipative tissues, shows regional variations in viscoelasticity between grey and white matter, as well as among different brain regions [245, 246].

While past studies have primarily emphasized elasticity, defined by stiffness, at the expense of viscosity [247], viscoelasticity plays a pivotal role in diverse biological processes. It impacts focal adhesion formation, cell motility, spreading, proliferation, mesenchymal stem cell (MSC) differentiation, cell migration, ECM synthesis, and spatiotemporal tissue organization [91, 248–250]. Interestingly, the viscoelastic properties of zygotes can predict human blastocyst formation shortly after fertilization [251].

At the tissue or organ level, viscoelastic behavior is crucial for maintaining homeostasis [91, 252]. For instance, in periodontal ligaments (PDLs), viscoelasticity plays a significant role in tissue stability. During periodontitis or dental trauma, the loss of PDL viscoelasticity disrupts ECM interactions, accelerating tissue damage [241]. However, restoring viscoelastic properties can mitigate this damage by upregulating the integrin-FAK pathway and promoting cytoskeletal remodeling. This process facilitates cell spreading, proliferation, and fibrogenic differentiation, ultimately aiding in tissue regeneration [243, 250]. In MSCs, viscoelastic signals trigger the activation of TRPV4 channels, which in turn induces the nuclear localization of Runt-related transcription factor 2 (RUNX2), promoting osteogenic differentiation in a YAP-independent manner [253]. Conversely, restricted cell volume expansion has been linked to the activation of interleukin 1β (IL-1β) signaling, which drives the osteoarthritic phenotype in chondrocytes [90].

### Residual stresses and compressive stress

Residual stresses play a crucial role in modulating stress distributions and influencing the mechanobiological environment of cells in soft biological tissues [63]. In muscles, these stresses help optimize muscle contraction length and impact various physiological processes like arterial extension, inflation, and torsion [254–256]. Residual stresses can prevent stress concentration, maintain compliance, and influence vessel permeability [257, 258]. For instance, in coronary plaque, residual stresses redistribute the stress across the vessel wall, decreasing strain on the inner wall while increasing it on the outer wall [51]. In contrast, studies suggest that residual stresses generated by ventricular volume reduction have minimal effects on left ventricular function [259]. Residual stresses are not confined to the cardiovascular system but are also present in organs like the small intestine, ureter, esophagus, skin, brain, and trachea [260–265]. In the gastrointestinal tract, these stresses help reduce stress concentrations and play a role in shaping villi height [260]. Specifically, smooth muscle-generated residual stress activates the nuclear localization of YAP via a ciliogenesis-associated kinase 1 (CILK1)-dependent mechanism, upregulating proliferative genes such as EDN1, anoctamin 5 (ANO5), and C-C motif chemokine ligand 11 (CCL11), which drive gut elongation [266]. Intriguingly, the presence of high residual stresses in mature, healthy brains contrasts with reduced stresses in brains post-hemorrhagic stroke, which correlates with alterations in cellular density [267]. Beyond their direct physiological functions, residual stresses are instrumental in embryonic cardiac morphogenesis, influencing ventricular wall stress levels and potentially impacting left ventricular function [268].

Compressive stress regulates various cellular processes like regeneration, migration, and autophagy across different tissues [46, 269, 270]. For instance, a loss of apical compression post-injury can trigger muscle stem cell renewal, whereas increased apical compression prompts stem cells to revert to a quiescent state by upregulating specific Notch signaling genes, such as HER genes [46]. Compressive loads also regulate cementocyte-driven osteoclastogenesis via the sphingosine-1-phosphate (S1P)-S1PR1-RAC1 signaling axis [271]. Furthermore, cadherin-mediated cell adhesion plays a crucial role in response to compressive stress, driving cell unjamming in stressed monolayers without initiating EMT [269].

### Contractility

Contractility is fundamental to cellular dynamics, driven primarily by the ubiquitous actomyosin, which facilitates simple cell contraction. For instance, platelets exhibit a uniform, isotropic contraction that shrinks their overall size, facilitating the compaction of blood clots [272]. In contrast, polarized fibroblasts and epithelial cells generate anisotropic contractile stress on the surrounding cells or ECM, playing a crucial role in processes such as matrix remodeling and tissue morphogenesis [93].

At the microscale, contractility is pivotal in key biological processes like embryo morphogenesis [273] and genomic stability [274]. Through asymmetric division, the varying contractility of blastomeres determines pre-implanted cell positioning and fate specification. Blastomeres with lower contractility tend to adopt inner cell mass-like phenotypes [273]. Specifically, contractility regulates YAP subcellular localization, illustrating its role in mechanotransduction during mammalian embryonic development [275]. In scenarios where actomyosin-driven nuclear rupture induces DNA damage, modulating contractility has shown promise in mitigating excess DNA damage, particularly in clinical cases involving lamin A deficiency. Cells on a stiff ECM typically display reduced phosphorylation of lamin A and slower degradation by MMP2, which helps preserve genomic integrity [274]. Furthermore, RhoA/ROCK-mediated cytoskeletal contractility is also critical in integrin αvβ6-dependent TGF-β activation [276], influencing various pathophysiological processes such as wound healing, tissue fibrosis, immune responses, cancer progression, and metabolic disorders [213].

At the macroscale, fluid-filled intracellular, extracellular, and capillary spaces within muscles play significant roles in influencing muscle contractility and morphology [277]. In the heart, cardiomyocytes employ a mechano-chemical transduction mechanism, linking cardiac excitation to enhanced contractility via calcium flux, which is crucial for maintaining stroke volume and cardiac output [52].

**Table 2:** Impact of mechanical cues in non-malignant biological behaviors

Mechanical Cues	Tissues or Cells	Impact	References
Tensile stress	Skeletal muscles	Muscle movement, such as voice production	[155, 160]
	Cardiomyocytes	Cardiomyocyte hypertrophy, cardiac chamber maturation, and atrial arrhythmogenesis	[162–165]
	Vascular ECs	Angiogenesis	[156]
	Nervous system	Neural development and axon regeneration	[113, 157, 166]
	Fibroblasts	Wound healing and scarring	[168]
	Urothelium	Cell proliferation and urethral micturition reflexes	[158, 169]
	Drosophila crop	Satiety and feeding	[170]
	T cells	Chemotactic migration	[171]
	Embryonic tissues	Embryo morphogenesis	[36]
Shear stress	Vascular ECs	Vasodilation, cell proliferation, angiogenesis, cell survival, vascular inflammation, and atheroprotective or atheroprone phenotypes	[179, 180, 184, 185, 193]
	Urinary system	Renal functions such as proximal reabsorption	[195]
	Bronchial epithelial cells	ATP release	[198]
Hydrostatic pressure	Skeletal system	Osteogenesis and cell differentiation	[199, 202–204]
	Neural crest cells	Neural development	[200]
	Hela-Kyoto cells	Regulation of mitosis	[201]
	Immune cells	Inflammatory response	[38]
	Heart	Atrial electrical remodeling, even atrial fibrillation	[205]
Matrix stiffness	Embryonic stem cells	Mesoderm differentiation	[214]
	Skeletal system	Cell proliferation, cell spreading, cell adhesion, cell migration, and cell differentiation such as osteogenic differentiation	[215, 216]
	Neural crest cells	Epithelial-to-mesenchymal transition (EMT) and collective cell migration	[217]
	Nervous system	Neural development such as synaptic plasticity and regeneration, and restrict Alzheimer’s disease	[211, 218, 219]
	Tendon stromal cells	Cell stemness, synapse formation, and angiogenesis	[212]
	Immune cells	Maintaining T cell populations, and immune-enhancing or -suppressing responses	[213]
	Heart	Myocardial fibrosis progression and cardiac function decline	[226–229]
	Liver	Fibroblast-myofibroblast conversion and liver fibrosis	[232, 233]
Viscoelasticity	Periodontal ligaments	Tissue homeostasis, cell spreading, cell proliferation, cell differentiation, and tissue regeneration	[241, 243, 250]
	MSCs	Osteogenic differentiation	[253]
Residual stresses	Muscles	Optimizing muscle contraction length such as arterial extension, inflation, and torsion	[254–256]
	Blood vessels	Vessel permeability and stress redistribution	[257, 258]
	Gastrointestinal tract	Shaping villi height, cell proliferation, and gut elongation	[260, 266]
	Heart	Embryonic cardiac morphogenesis	[268]
Compressive stress	Muscle stem cells	Cell renewal	[46]
	Cementocytes	Osteoclastogenesis	[271]
Contractility	Platelets	Shrinking size and facilitating the compaction of clots	[272]
	Fibroblasts and epithelial cells	Matrix remodeling and tissue morphogenesis	[93]
	Pre-implanted cells	Embryo morphogenesis	[273]
	Cardiocytes	Genomic integrity	[274]
	Muscle cells	Muscle morphology, and cardiac output	[277, 52]

## Mechanical attributes within the tumor microenvironment

**Fig. 4:**
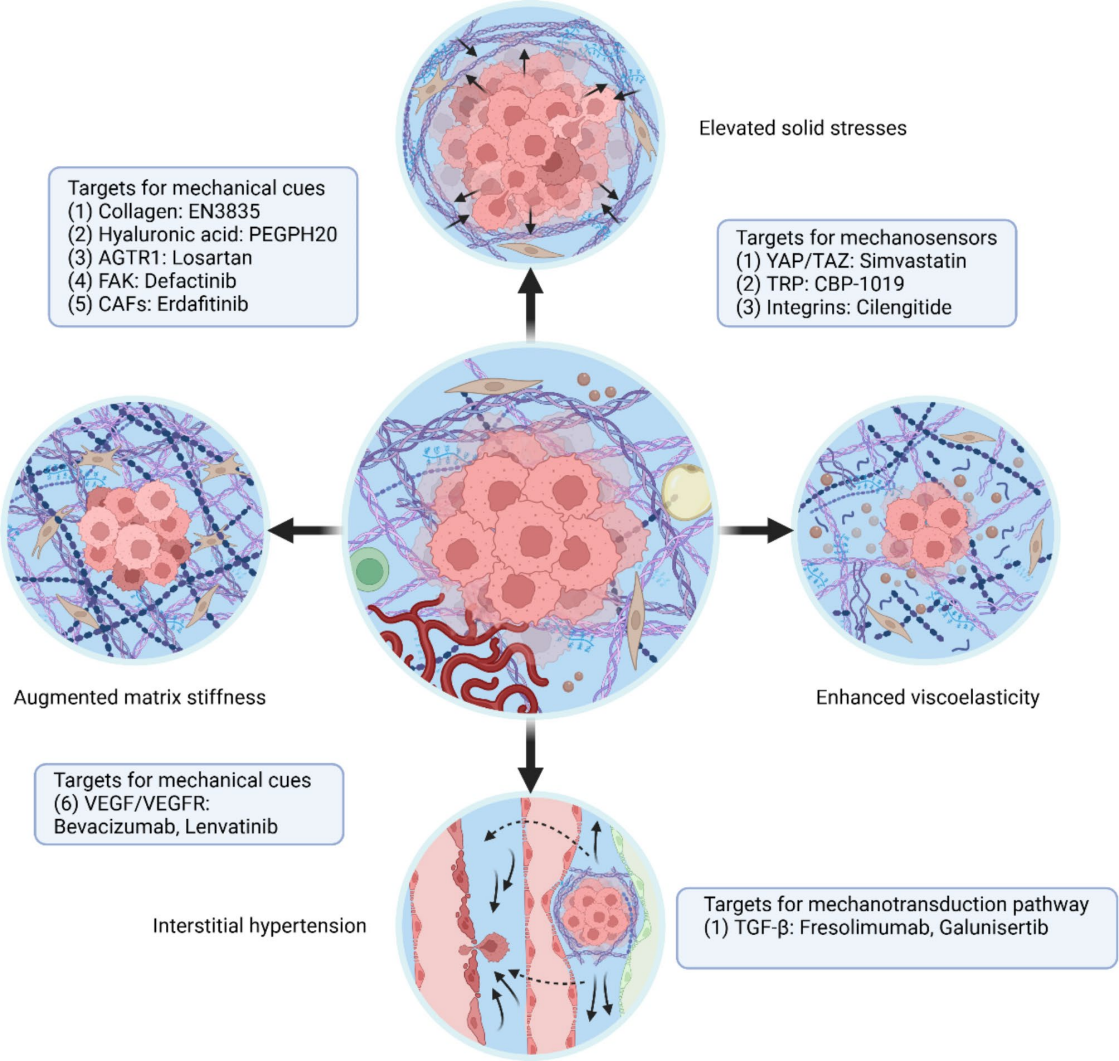
An overview of mechanical cues in tumor microenvironment. The tumor microenvironment exhibits distinct mechanical traits from the non-malignant tissues, including elevated solid stresses, interstitial hypertension, augmented matrix stiffness, and enhanced viscoelasticity. Cell growth in a confined space generates elevated solid stresses. Leaky and compressed blood vessels and impaired lymphatic drainage increase interstitial fluid pressure. Matrix deposition and crosslinking augment matrix stiffness. Accumulation of macromolecules leads to enhanced viscoelasticity. This figure was created using Biorender.com

The TME plays a critical role in driving various pathological changes in tumors. Among the dimensions of TME, it exhibits distinct mechanical traits from the non-malignant tissues, such as elevated solid stresses, interstitial hypertension, augmented matrix stiffness, and enhanced viscoelasticity (Fig. 4). These alterations can lead to diverse growth patterns, migration capabilities, metastatic potential, and dedifferentiation profiles across different types of tumors via cellular mechanotransduction, independent of immune and metabolic factors. Moreover, similar mechanotransduction processes can happen to other cellular components within TME, modulating their behaviors to either promote or suppress tumor development (Table 3).

### Solid stresses

Solid stresses, also known as residual stresses, encompass the cumulative mechanical stresses exerted by the solid and elastic components within the TME.

The initiation of solid stresses typically originates from an increase in tissue volume due to factors such as cell infiltration, proliferation, and matrix deposition. This augmented volume displaces existing viscoelastic structures within and around the tumor, giving rise to solid stresses within the tumor and its neighboring tissues [69, 278]. For instance, the rapid proliferation of cancer cells strains the TME, where cells located in the central region typically encounter predominantly compressive stress in all directions. In contrast, periphery cells experience a combination of circumferential tensile stress and radial compressive stress [279]. Therefore, the reduction of cancer cells via anti-tumor treatments can alleviate solid stresses and decompress blood vessels. Additionally, orchestrated displacement of normal tissue significantly influences solid stresses [280]. Tumors can exhibit varying growth patterns, some favor expanding as well-circumscribed nodular masses, generating considerable solid stresses by pushing the surrounding tissue. Conversely, invasive rather than cohesive tumors can infiltrate through normal tissue, creating less solid stresses. Furthermore, components like hyaluronic acid (HA) present in the glycosaminoglycan matrix can swell due to water absorption, leading to the generation of solid stresses [281]. Intriguingly, the interaction between collagen and HA is considered crucial for decompressing tumor vessels, particularly in collagen-rich tumors [282]. Additionally, cell movements and repair activities within the TME contribute to contractile stress through actomyosin-mediated mechanisms. This cellular contractility generates tensile forces that contract ECM components [283], leading to tension within the tumor, generally balanced by compression in other elements, resulting in the manifestation of solid stresses within TME [69]. Specifically, certain CAFs activated by TGF-β can acquire a myofibroblastic phenotype capable of generating substantial contractile stress and encapsulating cancer cells by excessive ECM production. This capsule, far from being a passive physical barrier, actively exert compressive stress on cancer cells through actomyosin contractility, which also contributes to solid stresses [284].

#### Impact of solid stresses

The impact of solid stresses on cancer cell biology has been recognized since 1997 when it was observed that accumulated solid stress inhibited the growth of tumor spheroids [64]. Further studies have consistently demonstrated this inhibitory phenomenon in various in vitro settings, irrespective of host species, tissue origin, or differentiation state [64]. The inhibitory stress for multicellular tumor spheroid growth in agarose matrices has been identified to range from 45 to 120 mmHg, exceeding typical blood pressure in tumor vessels, leading to compression and potential collapse of vascular or lymphatic structures within tumors. This phenomenon results in compromised blood flow, hypoxia, impaired lymphatic drainage, reduced drug delivery, and diminished treatment efficacy in solid tumors [65, 285–287]. At the cellular scale, stress-induced growth inhibition is associated with increased cellular packing density, potentially preventing apoptosis through mechanisms related to cell volume or shape transduction [64]. Solid stresses can also directly influence tumor biology by inducing phenotypic transformation in periphery cells, triggering collective cell migration in a RhoA/ROCK-dependent pathway [288].

Cells respond to mechanical forces through various mechanosensitive mechanisms involving cell-ECM and cell-cell interactions, stress-sensitive ion channels, and pathways associated with ECM component deformation [62]. On a micro-scale, the nucleus acts as a mechanosensitive organelle capable of responding to solid stresses via nuclear pore complexes and associated proteins [289–291]. When subjected to deformation, the nucleus experiences alterations in nuclear envelope plasticity and nuclear translocation, the stretching of chromatin, and subsequent changes in gene expression patterns [292, 293]. Additionally, the crucial role of YAP/TAZ has been observed in responding to solid stresses within the TME [294, 295]. Activation of the YAP/TAZ pathway contributes to various aspects of tumor malignancy, including suppression of mitochondria-induced apoptosis by upregulating BCL2 family members [296], promotion of cell proliferation by inducing proto-oncogenes such as KRAS and c-MYC [297, 298]. Specifically, the mechanosensing capabilities of cancer cells allow them to detect the compressive stress induced by CAFs, leading to YAP nuclear exclusion and decreased proliferation rates [284, 299].

In response to solid stresses, ECM undergoes a range of alterations in key elements such as fibronectin and collagen [300, 301]. The integrin α5β1 of liver cancer cells possesses the ability to detect and respond to tensile stress, triggering the activation of integrin-Src-FAK, AKT, and ERK signaling pathways [53]. Subsequently, the interplay between the upregulation of integrins and the ECM initiates the remodeling of collagen type I and fibronectin, thus facilitating tumor progression and angiogenesis [53, 302].

Apart from cancer cells, solid stresses also affect other cells within TME. For instance, compressive stresses can induce the dedifferentiation of adipocytes via Wnt/β-catenin signaling activation, yielding a unique adipocyte subset with distinct properties (i.e., persistent self-renewal, serial clonogenicity, and potential myofibrogenesis) that support tumor growth [303]. Additionally, solid stresses induced by hyper-proliferative adjacent crypts can stimulate tumorigenesis in non-cancerous epithelial cells via the activation of the Ret-β-catenin-MYC pathway [304]. Activation of latent TGF-β, which is found in the ECM, on the surface of Tregs and tumor-associated platelets, has been observed in response to solid stresses. This activation occurs in an integrin αvβ8-dependent manner and is associated with cancer progression by inhibiting anti-tumor immune responses [213, 305, 306].

In dynamic cellular environments, equally fit cell populations are continuously replenished by stochastic replacement events. Conversely, inferior cells are actively eliminated by their more vigorous counterparts. As cancer progresses and cells proliferate, the consequent cellular crowding triggers competition among cell populations for nutrients and spatial resources. This phenomenon represents a distinctive surveillance mechanism termed cell competition, where highly proliferative cells can outcompete and eliminate neighboring suboptimal cells, ensuring optimal tissue fidelity. This process serves dual roles as both a tumor-suppressive mechanism and a tumor-promoting mechanism, thus critically influencing cancer initiation and progression [307–309]. For instance, heterogeneous expression of YAP can drive cell competition, inducing apoptosis and the subsequent elimination of cells with lower YAP. In scenarios where certain cancer cells overexpress YAP, the more tumorigenic YAP^high^ clones will be dominant, facilitating tumor progression [310]. Whereas neighboring YAP^high^ non-cancerous cells possess the ability to constrain tumor expansion. Moreover, solid stresses arising from the uneven growth of cell layers have been proposed as a mechanical driver of cell competition, further highlighting the intricate interplay between cellular mechanics and tumor dynamics within TME [311, 312].

These findings indicate that solid stresses play a crucial role in regulating tumor growth at both the macro and cellular scales. This highlights how solid stresses impact tumor progression as well as the distribution and efficacy of therapeutic agents within TME [64]. Understanding the mechanical forces within tumors can be pivotal in developing novel strategies for controlling tumor progression and enhancing the delivery of therapeutic interventions.

#### Treatment addressing solid stresses

A novel therapeutic approach has emerged that targets the key components of solid stresses, particularly the essential elements within the ECM. By focusing on degrading matrix components and reducing fibrosis, various issues within the tumor microenvironment can potentially be reversed with the aid of specific drugs.

Angiotensin inhibitors (e.g., losartan) have been proposed as agents to reduce solid stresses, particularly to alleviate compressed vessels. One of the candidates, losartan, has shown efficacy in reducing stromal collagen and HA production by inhibiting profibrotic signals like TGF-β1, cellular communication network factor 2 (CCN2), and endothelin-1 (ET-1) via angiotensin II receptor 1 (AGTR1) inhibition [282]. This reduction in solid stresses can lead to the decompression of collapsed vessels, thus improving tumor perfusion, alleviating hypoxia, reducing downstream tumor-promoting pathways, and ultimately enhancing drug delivery and efficacy [282].

In a phase II clinical trial (NCT01821729), losartan collaborated with cytotoxic therapy demonstrated promising results in unresectable, locally advanced PDAC, doubling the conversion rate to resectable tumors and enhancing overall survival (OS) [313, 314]. Similarly, in preclinical studies, PEGylated recombinant human hyaluronidase (PEGPH20) successfully reduced fibrosis in PDAC by targeting stromal HA, leading to increased OS when combined with gemcitabine [315]. Other potential approaches with similar promise include inhibiting the vitamin D receptor [316, 317], disrupting sonic hedgehog signaling [318], and targeting C-X-C chemokine receptor type 4 (CXCR4) signaling [319]. Notably, alleviating stress through these methods could enhance the effectiveness of different treatments, including immunotherapy. For instance, the combination of a FAK inhibitor (defactinib) that decreases tumor desmoplasia, anti-PD-1 (pembrolizumab), and gemcitabine demonstrated favorable safety profiles, promising initial efficacy, and exhibited biomarker activity in infiltrative T cells in PDAC within a phase I clinical investigation (NCT02546531) [320]. These findings present opportunities to investigate innovative strategies for addressing solid stresses in the TME and enhancing therapeutic outcomes for individuals battling cancer.

### Fluid stresses

Within the TME, a complex interplay of fluid stresses is generated by forces induced by fluid substances, such as shear stress and hydrostatic fluid pressure. Notably, interstitial fluid pressure emerges as a key characteristic of the TME, assuming a central and critical role in the cellular mechanotransduction of fluid stresses in TME.

In healthy organs, blood and lymphatic vessels work harmoniously to maintain fluid homeostasis and low interstitial fluid pressure (IFP). However, this balance is disturbed by abnormalities in many solid tumors. Tumor angiogenesis creates hyperpermeable blood vessels [321, 322], while solid stresses compress the blood and lymphatic vessels, contributing to uniformly elevated IFP collaboratively, hindering drug delivery into the tumor [323]. The presence of elevated IFP in tumors was first noted in Brown-Pearce carcinoma in 1950, with levels varying across tumor types, from < 1 kPa (7.5 mmHg) in brain tumors to 5 kPa (37 mmHg) in renal cell carcinomas [62, 324]. To note, both the osmotic and hydrostatic IFPs are often found to be elevated in solid tumors [78]. Within a tumor, IFP is fairly consistent but precipitously drops at the tumor margin, promoting fluid flow toward nearby tissues, which exposes extravascular cells to varying shear stress levels [325]. Despite similarities in the generation processes of solid stresses and IFP, they represent distinct mechanical stresses with unique origins and impacts [326].

#### Impact of fluid stresses

Physically, elevated IFP hinders the extravasation of drugs and immune cells into tumors by inducing direct tissue deformation and promoting an outward interstitial flow that opposes inward diffusion at the tumor margin. This effect impedes transcapillary transport, reducing drug uptake and retention in various cancers like breast, colorectal [327], uterine cervical [328], head and neck carcinomas [329], and metastatic melanoma [330, 331]. This outward flow of interstitial fluid from the tumor toward surrounding tissues promotes tumor invasion and growth by facilitating the transport of growth factors and cancer cells into neighboring normal tissue and peritumor lymphatics. Consequently, elevated IFP is responsible for the poor penetration and heterogeneous distribution of therapeutic agents like monoclonal antibodies [332].

IFP has been shown to exert various effects on both cancer cells and non-cancerous cells within the TME. Studies have demonstrated that interstitial fluid flow can upregulate the expression of integrin αvβ3 in cancer cells and CXCR4 in bone, respectively, coupled with an increased level of MMP9 [333]. Despite the co-existing IFP-induced cancer cell apoptosis via TGF-β1 signaling, these molecular changes can ultimately promote tumor progression and metastasis [333–335]. In addition, cadherins have been identified to be involved in the transmission of fluid stresses [336, 337]. Specifically, N-cadherin on CAFs and E-cadherin on cancer cells collaborate to facilitate collective cancer cell invasion, while IFP triggers β-catenin recruitment and adhesion reinforcement for further support [338]. Intriguingly, cell glycocalyx proteoglycan Glypican 1 acts as a key mediator in the crosstalk between IFP stimuli sensed by heparan sulfate and cancer cells within the TME, contributing to augmented cell migration and metastasis by MAPK signaling [339, 340]. Additionally, elevated IFP can trigger the upregulation of TGF-β in fibroblasts, which subsequently induces myofibroblast differentiation, leading to further fibroblast contraction and ECM stiffening, as well as increased production of TGF-β [341]. Notably, TGF-β plays a crucial role in immune regulation within TME, attracting various immune cells such as T cells, and neutralizing their anti-tumor functions synergistically with PD-1 signaling [341]. Furthermore, TGF-β is required for immature and tolerogenic DCs to promote the differentiation and proliferation of Tregs, enhancing immunosuppressive effects within the TME [342]. Apart from T cells, flow signals can also impact immune responses by facilitating the release of N-cadherin via A disintegrin and metalloprotease 10 (ADAM10), leading to NK cell dysfunction through the killer cell lectin-like receptor subfamily G member 1 (KLRG1) receptor [343]. Furthermore, IFP is also critical in the activation of specific signaling pathways that drive tumor progression and metastasis, including the FAK-ERK1/2 signaling pathway [344] and the PIEZO1-Src-YAP axis [345]. In contrast, exposure to IFP-induced shear flow suppresses the invasive phenotype of cancer cells by downregulating the activity and expression of MMPs [346] and induces cell cycle arrest via the BMP-SMAD1/5 pathway [347, 348].

The relationship between IFP and tumor neovascularization is intricate. IFP has been shown to induce endothelial sprouting by upregulation of MMP1, facilitating angiogenesis in tumors, with tumor IFP increasing following the initiation of tumor angiogenesis [349, 350]. Studies measuring IFP in human colon adenocarcinoma and murine carcinoma have demonstrated tremendous changes in IFP as tumor neovasculature develops. Initially, IFP is close to atmospheric pressure in avascular tumors. However, as vascular sprouts and loops begin to form and vasculature establishes with functional blood flow, IFP increases substantially. This relationship between tumor interstitial hypertension and the progression of tumor neovasculature becomes more clear after the comparison to microvascular pressure [351]. Moreover, there also exist several potential factors that are associated with IFP and tumor vascular architecture, encompassing PIEZO1-mediated regulation of endothelial NOS and calpain 2 [352], a non-canonical Notch downstream effector RAC1 [353], YAP/TAZ-induced expression of cysteine-rich angiogenic inducer 61 (CYR61), connective tissue growth factor (CTGF), and ankyrin repeat domain 1 (ANKRD1) [354], as well as JNK signaling [355].

Notably, a substantial portion of HMGB1 present in the interstitial fluids of TME is oxidized. Unlike its reduced isoform, oxidized HMGB1 acts as a tolerogenic signal through a receptor for advanced glycation end-products (RAGE)-dependent mechanism [356]. Blocking extracellular HMGB1 has been shown to inhibit tumor growth by the profound remodeling of the immune microenvironment and the enhancement of checkpoint inhibitor-based immunotherapy. Additionally, IFP has been linked to increased resistance to doxorubicin in human breast cancer cells, mediated by the overexpression of ATP-binding cassette subfamily C member 1 (ABCC1) [357]. Therefore, lowering tumor IFP through specific targeted antagonists is indeed a promising strategy to improve the efficacy of anti-tumor treatment.

#### Treatment addressing fluid stresses

One therapeutic approach to target the elevated IFP in solid tumors involves normalizing the leaky and tortuous vasculature, thereby restoring the aberrant tumor vasculature to a more functional phenotype similar to normal blood vessels [358, 359]. This can be achieved through carefully dosed anti-angiogenic therapy. Anti-angiogenic agents target various regulators engaged in tumor angiogenesis (i.e., VEGF, fibroblast growth factor (FGF), PDGF, epidermal growth factor (EGF), TGF-β, HIF1, NF-κB, MMPs, and integrins), with the VEGF and its receptor VEGFR signaling pathway playing a dominant role in this process [359]. Therefore, monoclonal antibodies and small molecule tyrosine kinase inhibitors that target the VEGF/VEGFR pathway, such as Bevacizumab, Ranibizumab, Sorafenib, and Sunitinib, present promising strategies for anti-tumor treatment.

Beyond single anti-angiogenic therapy, the combination of VEGFA neutralization and angiopoietin 2 (ANGPT2) inhibition has been shown to normalize tumor vasculature, which can improve drug delivery, increase sensitivity to radiation therapy, and enhance immune cell infiltration in TME [359–361]. Similarly, combining anti-angiogenic therapy with immune checkpoint blockade has been demonstrated to enhance the efficacy of cancer immunotherapy by normalizing vascular-immune crosstalk and potentiating the anti-tumor immune response [362, 363].

Furthermore, the role of solid stresses in compressing blood and lymphatic vessels is critical to elevated IFP. Therefore, alleviating solid stresses with hydrolases or hyaluronidases can target ECM degradation, decompressing these vessels, and improving perfusion and flow within TME [286, 364]. This dual strategy of targeting both solid stresses and abnormal vasculature holds promise for enhancing the delivery and effectiveness of anti-tumor therapies.

Hopefully, studies have shown that curvature-mediated properties play a significant role in determining the ability of nanoparticles to efficiently navigate through TME against elevated IFP, contributing to superior intratumoral accumulation and anti-tumor efficacy [364].

### Matrix stiffness

Stiffness, an intrinsic material property of tissues, represents a crucial mechanical abnormality in tumors. It is distinct from solid or fluid mechanical stresses, which refer to forces exerted on a material. Tumor stiffness can vary significantly across different types of cancer, ranging from 1 kPa in brain tumors to 70 kPa in cholangiocarcinomas [62].

Increased tissue stiffness is a key mechanical abnormality observed in tumors and has been utilized as a marker for diagnosis and prognosis through techniques such as tomoelastography [365, 366]. A greater stiffness is commonly associated with malignant tumors rather than benign ones, including the carcinoma of breast [367], pancreas [368], liver [369], and prostate [370].

As reviewed in non-cancerous conditions, ECM deposition and post-translational modifications of ECM components (i.e., nonenzymatic glycation and crosslinking of collagen) play a crucial role in modulating tissue stiffness [50]. Furthermore, mechanical stresses such as tensile and compressive forces, can also contribute to alterations in ECM stiffness via a phenomenon termed strain-stiffening [299, 371]. Collagen fibers within the ECM can stretch when subjected to tension, which occurs during cell contraction or tumor expansion. The stretching of collagen fibers increases tissue stiffness, triggering focal adhesion contractility in surrounding CAFs, which ultimately leads to a cycle of increased matrix deposition and stiffening [69, 372, 373]. At a subcellular scale, mechanical stresses applied to cell nuclei can increase the stiffness of the nucleus through the phosphorylation of emerin, a nuclear envelope protein that helps maintain the structural stability of the nucleus [374].

#### Impact of matrix stiffness

The stiffness of TME plays a pivotal role in various aspects of cancer biology, including cell proliferation [375], angiogenesis [376], metabolism [377, 378], invasion [87], migration, and metastasis [45, 377]. Changes in substrate stiffness can influence collective cell migration by promoting EMT in vivo, a critical process in tumor progression and metastasis [217].

In TME, ECM stiffening can mechanically activate metabolic pathways such as glycolysis and glutamine metabolism in both cancer cells and CAFs, leading to the coordination of non-essential amino acid flux, which influences the metabolic interactions between different cell types within the TME. For instance, CAFs can produce aspartate in this altered metabolic state, which supports cancer cell proliferation. Concurrently, cancer cells can release glutamate, which helps maintain the redox balance of CAFs and contributes to ECM remodeling, ultimately promoting tumor growth and malignancy [378]. Additionally, the rewiring of glutamine metabolism leads to the promotion of microtubule (MT) glutamylation and self-stabilization, thus enhancing cancer cell invasion [377]. In glioblastomas, a less stiff microenvironment that resembles the brain tissue can induce a glycolysis-dominant metabolic state in cancer cells. This metabolic shift is mediated by signaling through CD44 and integrin receptors, supporting invasive phenotypes in the cancer cells [379]. Furthermore, ECM stiffness can also influence the expression of immune checkpoint proteins such as PD-L1. Cancer cells cultured on a stiffer medium exhibit increased expression of PD-L1 in an actin-dependent manner, which serves as an adaptive immune resistance strategy, allowing cancer cells to escape immune surveillance and evade immune responses [380]. In addition to these effects, ECM stiffness plays a role in controlling VEGF_165_ secretion and neuroblastoma angiogenesis through the YAP-RUNX2-serine-arginine-rich splicing factor 1 (SRSF1) axis [376]. In women with high mammographic density, stiff breast tissue is linked to elevated levels of phosphorylated ERK, progesterone receptors, and receptor activator of NF-κB (RANK) signaling [367]. Notably, profibrotic phenotypes in response to substrate stiffness can be preserved as mechanical memory by microRNA-21, sustaining the effects of mechanical stimuli on cancer cells [381].

Studies have shown that CAFs play a crucial role in modulating the composition and physical properties of ECM, thus regulating cancer cell metastasis [382, 383]. In desmoplastic tumors, CAFs are activated to adopt a profibrotic phenotype in response to excessive TGF-β signals. This activation leads to increased collagen production by CAFs, contributing to ECM stiffening through a positive feedback loop [384, 385]. Notably, different subpopulations of CAFs may have opposing effects on tumor behavior [386]. For instance, myofibroblastic CAFs (myCAFs) can secrete factors such as HA that promote tumor growth, while inflammatory CAFs (iCAFs) may release hepatocyte growth factor (HGF) that supports tumor progression [386]. In contrast, the presence of type I collagen, also produced by myCAFs, can exert tumor-suppressive effects by mechanical restriction, counteracting the pro-tumorigenic signals induced by ECM stiffening [386]. Therefore, therapeutic strategies that selectively target CAF-derived tumor-promoting mediators while preserving tumor-suppressive factors, have the potential to remodel CAF subpopulations and enhance anti-tumor phenotypes.

Beyond cancer cells and CAFs, other cells within the TME also contribute to modulating ECM stiffness and influencing cancer progression. Studies have shown that increased liver stiffness, often associated with cirrhosis, can promote the progression of hepatocellular carcinoma (HCC) [387]. HSCs are one such cell type that plays a crucial role in the TME of HCC. Intriguingly, HSCs can release the same factors that CAFs secrete, such as HGF and type I collagen, whereas these factors function distinctively from those observed in CAFs. For instance, quiescent HSC-derived HGF can protect against hepatocyte death and HCC development, while type I collagen from activated myofibroblastic HSCs promotes tumor proliferation and progression through increased stiffness and activation of TAZ in peritumoral hepatocytes and discoidin domain receptor 1 (DDR1) in established tumors [375]. Specifically, the nuclear translocation of p300 acetyltransferase via AKT signaling enhances the expression of metastasis-associated genes (i.e., CXCL12), playing a critical role in stiffness-induced activation of HSCs into tumor-promoting myofibroblasts [388].

In the complex landscape of the TME, high levels of ECM stiffness can exert profound effects on immune cell behavior, particularly in the context of cancer progression and immune evasion.

The deposition of collagen and the stiffening of the matrix in the TME can polarize macrophages to adopt a tumor-promoting M2-type phenotype, with the upregulation of CD206, IL-4, IL-10, and TGF-β and less production of reactive oxygen species (ROS). This transition is facilitated by various sensors like PIEZO1, TRP ion channels, and integrins, as well as transducers including YAP/TAZ, Rho/ROCK, FAK, and LOX. Consequently, this process further intensifies the stiffness of the ECM [389].

Physiologically cultured DCs exhibit reduced proliferation, activation, and cytokine production. In contrast, elevated stiffness in the microenvironment enhances DC activation and triggers heightened glucose metabolic pathways, promoting an adaptive immune response phenotype mediated by TAZ and PIEZO1 [390]. More specifically, the stiffness-induced stimulation of PIEZO1 integrates the SIRT1-HIF1α-dependent metabolic pathway and calcium-calcineurin-NFAT signaling pathway, orchestrating the reciprocal differentiation of Th1s and Tregs via DC-derived IL-12 and TGF-β1 [391]. The migration of DCs within the ECM hinges on integrin-based adhesion structures. The surface expression of C-type lectin receptors (CLRs) on immature DCs is downregulated by the stiff ECM in the TME, inhibiting the binding of internalized antigens [392]. Notably, the immunosuppressive characteristics of DCs can be induced by ECM stiffness, thereby contributing to resistance to immunotherapy. Whereas ECM degradation is essential for the migration and accumulation of DCs [393].

Increased stiffness within the TME can lead to the enlargement of a subset of highly active NK cells. This enhanced activation of NK subsets is dependent on the major histocompatibility complex class I polypeptide-related sequence A (MICA), which contributes to a holistic activation of NK cells and improves cancer cell lysis, leading to the release of tumor antigens [394]. However, the elevated stiffness of the ECM can disrupt the balance between tissue inhibitors of metalloproteinases (TIMPs) and MMPs, which impedes the anti-tumor functions of NK cells, potentially compromising their cytotoxic activity against cancer cells within the TME [393].

#### Treatment addressing matrix stiffness

Given that ECM stiffness can influence cellular behavior and facilitate tumor progression within TME, targeting ECM stiffness could potentially shift non-cancerous cells towards an anti-tumor state and offer a novel approach to anti-tumor therapy. Currently, several strategies can be employed to counteract the impact of matrix stiffness on tumor progression: reducing matrix protein production, breaking down matrix proteins and crosslinking, and targeting mechanosensors and mechanotransducers activated by matrix stiffness [389].

For instance, direct targeting of ECM components by enzymes like hyaluronate lyase or collagenase can enzymatically decrease matrix protein production and crosslinking, leading to reduced matrix stiffness [279, 395]. In addition, CAFs and TGF-β are the key drivers of matrix protein production within TME. Erdafitinib, a pan-FGFR inhibitor, has shown promising outcomes depending on specific CAF subpopulations, necessitating precise diagnostic and therapeutic approaches tailored to these CAF subsets [396, 397]. Addressing the multifaceted role of TGF-β in fibrosis and tumorigenesis, targeting TGF-β signaling could be a potential avenue for cancer treatment. Neutralizing antibodies targeting TGF-β, like fresolimumab, have demonstrated improved OS rates in breast cancer patients when combined with local radiotherapy [398]. Similarly, galunisertib, a selective TGF-β receptor kinase inhibitor, has shown enhanced OS in pancreatic cancer patients when combined with gemcitabine therapy [399].

In addition to curbing matrix protein production, degrading matrix proteins within TME is also a promising strategy. However, ECM degradation by MMPs may also increase cancer invasion and metastasis [400], thus urging a comprehensive understanding of these matrix degraders. Notably, targeting mechanosensors and mechanotransducers activated by matrix stiffness can directly attenuate downstream signaling pathways, including PIEZO1, TRP channels, integrins, and YAP/TAZ [389]. Novel approaches emerge to target abnormal tumor metabolism, such as metformin, an activator of adenosine monophosphate-activated protein kinase, which can reduce fibrosis and stiffness [401]. Furthermore, inhibiting glutamine metabolism pharmacologically has also been shown to impede cancer aggressiveness by reducing stiffness [377]. Indeed, the emerging perspective that elevated tumor stiffness is associated with augmented malignancy opens up opportunities to capitalize on this characteristic by devising mechanosensitive treatments [402]. By understanding and leveraging the mechanical properties of tumors, innovative therapeutic approaches can be developed to target and potentially disrupt the aggressive behavior of tumors influenced by increased stiffness.

### Viscoelasticity

In addition to matrix stiffness, the TME possesses another important material characteristic known as viscoelasticity, comprising both viscosity and elasticity. Here, we focus specifically on viscosity when discussing the enhancement of viscoelastic properties, as elasticity or stiffness has been previously addressed in earlier sections. Physically, this property can be influenced by the presence of macromolecules, such as mucins produced by resident epithelial and cancer cells, which play a significant role in viscoelastic behaviors within the TME [403]. In addition, the growth of primary tumors can lead to the compression of lymphatic vessels, impairing drainage and resulting in the gradual accumulation of macromolecules over time [62]. Moreover, the increased degradation of the ECM at the tumor site can worsen macromolecular crowding, contributing to a rise in viscosity within the TME [404].

#### Impact of viscoelasticity

Viscoelasticity plays a crucial role in various tumor behaviors, including cancer cell motility [249, 405], proliferation, migration [406, 407], progression [408], EMT [250], extravasation, and colonization [409].

Studies have revealed that elevated viscoelasticity with the TME can initiate a series of cellular responses in cell migration and cancer dissemination. This cascade begins with actin remodeling mediated by the ARP2/3 complex, leading to the polarization of the Na^+^/H^+^ exchanger 1 (NHE1) and subsequent cell swelling. This NHE1 activation further enhances RhoA-dependent cell contractility via the TRPV4 channel. The interplay between actin remodeling, NHE1-mediated swelling, and RhoA-based contractility collectively contributes to increased cell motility in the presence of elevated viscoelasticity [409]. Additionally, pre-exposure to high viscoelasticity equips cancer cells with a TRPV4-dependent mechanical memory via the Hippo pathway, ultimately enhancing cancer cell migration, extravasation, and colonization [409].

Specifically, the viscoelastic property of the TME can play a crucial role in regulating the dynamics and symmetry of cancer cell growth through various signaling pathways. Upon stimulation by the viscoelastic matrix during the G_1_ phase, TRPV4 activates the downstream phosphatidylinositol 3-kinase (PI3K)/Akt pathway, promoting cytoplasmic translocation of the cell cycle inhibitor p27^Kip1^, which allows the S phase entry and cell proliferation [407]. In addition, viscoelasticity can induce symmetry breaking in an ARP2/3 complex-dependent manner, promoting invasive protrusions, YAP nuclear translocation, and EMT for oncogenesis [250].

In patients with Type 2 diabetes mellitus, the accumulation of advanced glycation end-products (AGEs) in ECM leads to architecture remodeling and viscoelasticity enhancement without significant elastic changes. High levels of AGEs coupled with augmented viscoelasticity, along with oncogenic β-catenin signaling, can promote cancer cell proliferation and invasion via an integrin β1-tensin 1-YAP mechanotransductive pathway. Whereas strategies targeting AGE decrease and inhibit tumor growth, suggesting that AGE-mediated structural modifications contribute to increased ECM viscoelasticity, driving cancer progression in vivo, regardless of changes in stiffness [408].

#### Treatment addressing viscoelasticity

Viscoelasticity is an intrinsic property of various tissues, not just limited to tumors. Therefore, drugs aimed at targeting the fundamental components of viscoelasticity may have widespread effects throughout the body. Interestingly, augmented matrix viscoelasticity has been linked to reduced effectiveness of the chemotherapy drug temozolomide in glioblastoma cells [410]. However, contrasting findings from another study indicate that increased viscoelasticity could actually enhance the sensitivity of glioblastoma cells to agents like temozolomide and carmustine [411].

These conflicting results further highlight the complexity of the relationship between viscoelasticity and drug efficacy in anti-tumor treatment, except for surgical interventions. Understanding the intricate interplay between viscoelasticity and drug response is crucial for developing targeted therapeutic strategies that can effectively combat cancer while minimizing potential adverse effects on normal tissues. Further research is required to elucidate the mechanisms underlying these divergent responses and optimize treatment approaches in cancer patients.

**Table 3:** Impact of mechanical cues within the tumor microenvironment

Mechanical Cues	Phenotype	Tissues	Experiment Type	Biological Mechanisms	Range^a^	References
Solid stresses	Tumor-promoting	Solid tumors	In vivo/in vitro	Compressed and damaged vessels; elevated IFP; hypoxia; reduced drug delivery	2.8 to 142.4 mmHg	[65, 285–287]
		Breast cancer	In vivo/in vitro	Enhanced migration; fibronectin deposition; augmented cell-matrix adhesion; YAP/TAZ/TEAD-AP-1 complex; Wnt/β-catenin-mediated adipocyte dedifferentiation	5.8 to 52.5 mmHg (~ 1.5 mmHg)	[288, 297, 303]
		Colorectal cancer	In vivo/in vitro	Wnt/β-catenin-YAP1-TBX5 complex; Ret-β-catenin-Myc pathway; integrin αvβ8-dependent TGF-β activation	0.4 to 5.6 kPa	[213, 296, 304]
		Pancreatic cancer	In vivo/in vitro	YAP1/TEAD2-E2F	11.5 to 18 mmHg	[298, 412]
		Liver cancer	In vitro	Integrin α5β1-Src-FAK, AKT, and ERK signaling pathways	6.4 to 15 mmHg	[53, 413]
		Glioma	In vitro	YAP-mediated cell competition	/	[310]
	Tumor-suppressive	Solid tumors	In vitro	Stress-induced growth inhibition	45 to 120 mmHg	[64]
		Breast cancer	In vitro	Enhanced apoptosis; reduced cell viability	5.8 to 58 mmHg	[288]
		Colorectal cancer	In vivo/in vitro	YAP nuclear exclusion; decreased proliferation	~ 11 kPa	[299]
Fluid stresses	Tumor-promoting	Breast cancer	In vivo/in vitro	Poor drug and nutrient distribution; HMGB1-RAGE pathway; ABCC1 overexpression	6 to > 30 mmHg (-2 to 6 mmHg)	[327, 356, 357]
		Liver metastasis of colorectal cancer	In vivo	Poor drug and nutrient distribution	9 to 33 mmHg (-2 to 6 mmHg)	[327]
		Uterine cervical cancer	In vivo	Poor drug and nutrient distribution	10 to 26 mmHg (0 to 3 mmHg)	[328]
		Head and neck carcinoma	In vivo	ADAM10-N-cadherin-KLRG1 receptor axis	4 to 33 mmHg	[329, 343]
		Metastatic melanoma	In vivo	Poor drug and nutrient distribution	2 to 41 mmHg (-1 to 3 mmHg)	[330, 331]
		Prostate cancer	In vitro/in vivo	Integrin αvβ3 and CXCR4-mediated MMP9 upregulation; PIEZO1-Src-YAP axis	0.005 mPa to 0.1 mPa; ~ 0.05 ml min^− 1^ flow rate	[333, 345]
		Epidermoid carcinoma	In vitro	E-cadherin/N-cadherin adhesion	/	[338]
		Renal carcinoma	In vitro	Heparan sulfate-Glypican 1-MAPK axis	~ 0.125 Pa shear stress	[339]
		Liver cancer	In vitro	FAK-ERK1/2 pathway	0.01 to 0.2 Pa shear stress	[344]
	Tumor-suppressive	Glioma	In vitro	Downregulation of the activity and expression of MMPs	0.009 to 0.068 Pa shear stress	[346]
		Osteosarcoma	In vitro	BMP-SMAD1/5-induced cell cycle arrest	0.08 to 0.3 Pa	[347]
		Urinary bladder transitional cell carcinoma	In vitro	BMP-SMAD1/5-induced cell cycle arrest	0.1 to 7 Pa shear stress (0.02 to 2 Pa shear stress)	[348]
Matrix stiffness	Tumor-promoting	Liver cancer	In vivo/in vitro	Activation of HSCs, TAZ, and DDR1; p300 nuclear translocation	> 12 kPa	[375, 388]
		Neuroblastoma	In vitro	YAP-RUNX2-SRSF1-VEGF_165_ axis	< 10 kPa to 2 GPa^b^	[376]
		Squamous cell carcinoma	In vivo/in vitro	YAP/TAZ-dependent glutamate/aspartate crosstalk	~ 8 kPa (~ 1 kPa)	[378]
		Breast cancer	In vivo/in vitro	Microtubule glutamylation	~ 50 kPa	[377]
		Glioblastoma	In vitro	Metabolic shift between glycolysis and OXPHOS	2.3 to 5.2 kPa (~ 0.3 kPa)	[379]
		Lung cancer	In vitro	Actin-dependent PD-L1 upregulation	20 to 30 kPa (0.5 to 5 kPa)	[380]
		Colon cancer	In vivo/in vitro	PIEZO1 deficiency; TGF-β1-induced differentiation of Tregs	~ 50 kPa (~ 2 kPa)	[391]
	Tumor-suppressive	Liver cancer	In vivo/in vitro	Hepatoprotection from quiescent HSC-derived HGF	> 12 kPa	[375]
		Liver metastasis of colorectal and pancreatic cancers	In vivo/in vitro	Type I collagen-induced mechanical restriction	~ 16 kPa	[386]
		Lymphoma	In vivo/in vitro	Enhanced DC metabolism and function	~ 50 kPa (~ 2 kPa)	[390]
		Colon cancer	In vivo/in vitro	PIEZO1 stimulation; IL-12-induced differentiation of Th1s	~ 50 kPa	[391]
Viscoelasticity	Tumor-promoting	Breast cancer	In vivo/in vitro	ARP2/3 complex-dependent symmetry breaking; invasive protrusions; YAP nuclear translocation; EMT; TRPV4-PI3K/Akt pathway; TRPV4-RhoA pathway	3.5 to ≥ 40 cP viscosity (~ 0.7 cP viscosity)	[250, 407, 409]
		Liver cancer	In vitro	Integrin β1-tensin 1-YAP pathway	~ 0.25 loss tangent (~ 0.21 loss tangent)	[408]

a: Table 3 summarizes the range of mechanical cues present within the TME, when available, the physiological range observed in normal tissues is provided in parentheses, as reported in the referenced literature

b: The stiffness of neuroblastoma varies according to its original sites. Tumors derived from the adrenal gland, retroperitoneum, and peritoneum typically exhibit stiffness of less than 10 kPa, whereas those originating from bone, joints, and articular cartilage range from 30 kPa to 2 GPa

**Table 4:** Typical clinical trials targeting mechanotransduction

Target	Drug	Target in Mechanotransduction Process	Mechanism	Tumor Type	Phase	Current Status	ClinicalTrials.gov ID
Collagen	EN3835	Solid stresses and matrix stiffness	Collagenase-mediated collagen digestion	Uterine leiomyoma (fibroids)	1	Favorable prognosis	NCT02889848
Hyaluronic acid	PEGPH20	Solid stresses and matrix stiffness	Hyaluronidase-mediated hyaluronic acid digestion	Stage IV pancreatic cancer	3	Failure	NCT02715804
AGTR1	Losartan	Solid stresses and matrix stiffness	Inhibition of profibrotic signals	Locally advanced pancreatic cancer	2	Favorable prognosis	NCT01821729
FAK	Defactinib	Solid stresses and matrix stiffness	Inhibition of FAK-associated desmoplasia	Advanced pancreatic cancer	1	Favorable prognosis	NCT02546531
VEGF/VEGFR	Bevacizumab	Fluid stresses	Anti-angiogenesis	Solid tumors	4	Favorable prognosis	NCT01588184
VEGF/VEGFR	Lenvatinib	Fluid stresses	Anti-angiogenesis	Refractory differentiated thyroid cancer	2	Favorable prognosis	NCT02702388
CAFs	Erdafitinib	Matrix stiffness	Inhibition of FGFR-mediated matrix production	Advanced solid tumors	2	Active, not recruiting	NCT04083976
TGF-β	Fresolimumab	Downstream signaling pathway	Inhibition of TGF-β signaling	Metastatic breast cancer	2	Favorable prognosis	NCT01401062
TGF-β	Galunisertib	Downstream signaling pathway	Inhibition of TGF-β signaling	Advanced or metastatic pancreatic cancer	2	Favorable prognosis	NCT01373164
YAP/TAZ	Simvastatin	Mechanosensor	YAP-mediated Treg dysfunction	Prostate cancer	2	Recruiting	NCT05586360
TRP	CBP-1019	Mechanosensor	Inhibition of TRPV6	Solid tumors	1b	Recruiting	NCT06576037
Integrins	Cilengitide	Mechanosensor	Inhibition of αvβ3 and αvβ5 integrins	Prostate cancer	2	Failure	NCT00121238

## Conclusions

Over the past few decades, remarkable progress has been achieved in the field of cancer therapy through the development of targeted approaches, which specifically interact with genetic vulnerabilities or molecular hallmarks present within cancer cells, typically situated within key canonical signaling pathways [414]. For instance, critical markers like HER2, BCR-ABL, and PD-1/PD-L1 have been targeted by medications such as trastuzumab, imatinib, and immune checkpoint inhibitors (ICIs), respectively [414]. Despite the diversity of therapeutic agents available, challenges persist in the delivery, permeation, and efficacy of drugs to tumors due to TME, which has garnered increased attention in recent years, exhibiting aberrations in immune response, metabolism, and mechanical properties. Particularly, the mechanical traits of the TME have received the least exploration and remain a novel field with the potential to offer new strategies for cancer treatment in the future (Table 4).

Advancements in research have revealed that primary TME is characterized by a diverse range of severe mechanical cues, such as elevated solid stresses, interstitial hypertension, augmented ECM stiffness, and enhanced viscoelasticity, playing a critical role in tumor behaviors and therapeutic response via cellular mechanotransduction. To date, the mechanical stimuli present in the TME can activate various mechanosensors, including PIEZO, integrin, TRP channels, and YAP/TAZ. Fortunately, cellular mechanotransduction is not confined exclusively to the cancerous context [50]. Given the ubiquity of this process, insights from physiological settings and non-cancerous diseases may offer valuable parallels, references, and shared characteristics for tumors, outlining a more comprehensive blueprint for the regulatory networks and downstream pathways of key mechanosensors. This serves as a crucial piece in unraveling the mechanical microenvironment of cancer. Therefore, future research should advance synergistically in both cancer and non-cancer domains, with mutual exchange of knowledge and collaborative progress, facilitating cross-disciplinary advancements in both fields.

Notably, current research on the mechanical properties within the TME predominantly focuses on individual cell types or isolated mechanical stresses, potentially overlooking the interaction among different cell types and the complex array of mechanical cues. Indeed, the transmission of forces within the TME involves all constituents, whether cells or non-cellular components, spatially collaborating to conduct forces toward surrounding tissues, regardless of the involved mechanoreceptors and pathways. Moreover, in contrast to biochemical pathways, where factors operate in separate signaling pathways with limited interaction, mechanical forces interact directly within the shared physical space of the TME. For instance, cell proliferation within the TME establishes an intercellular network of mechanical forces. Marginal cells encounter circumferential tension and radial compression, while those in the core receive compressive stress in all directions, both contributing to the solid stresses within the TME. Hence, future studies should focus on establishing a collaborative framework that mimics the interaction of various mechanical cues and diverse components of the TME, thereby enhancing the comprehension of the complex interplay within the TME.

As research delves deeper into exploring the therapeutic potential of the mechanical characteristics within TME, concerns have emerged regarding the ubiquitous distribution of mechanical cues and their downstream signaling cascades throughout the body. This issue raises challenges, as direct targeting of mechanosensors lacks high specificity and carries considerable risks, thus underscoring the importance of precision targeting to develop effective treatments. Moreover, specific mechanical properties play a dual role in both facilitating and inhibiting tumor behaviors, thus introducing complexity to treatment strategies. For instance, solid stresses derived from ECM deposition can physically hinder tumor expansion while simultaneously compromising vascular function with a diminished efficacy of immunotherapy. Introducing direct ECM degradation agents in such scenarios may inadvertently disrupt physical barriers and potentially heighten the risk of metastasis [400]. Intriguingly, a noteworthy phenomenon exists, for example, compressive stress of 5.8 mmHg, which closely resembles conditions in the native breast TME, can stimulate the migration of cancer cells. However, stresses exceeding 5.8 mmHg significantly trigger apoptosis, with only 40% of cells viable at 58 mmHg, demonstrating tumor-inhibitory effects [288]. This provides further evidence that there is a specific force range in which mechanical cues may exhibit tumor-inhibitory properties, rather than promoting tumor progression, thereby complicating the development of targeted therapies. What’s worse, intricate interactions have been verified among mechanical signals. For instance, the release of macromolecules upon matrix degradation exacerbates viscoelasticity, presenting a therapeutic conundrum when attempting to target both matrix stiffness and viscoelasticity directly. These complexities might shed light on why therapeutic agents targeting these mechanical features often fail to meet anticipated outcomes in clinical trials [389], highlighting the need for further investigation into key effector molecules with greater tumor specificity.

Fortunately, it is becoming increasingly evident that established drugs designed to target canonical biological markers may also impact these mechanical features. For instance, overexpressed AGTR1 has been newly identified as a marker associated with poor response to ICI therapy, particularly in CAFs within a stiff and cold TME. Further investigation has revealed a potential targeted strategy utilizing angiotensin receptor blockers (ARBs) to inhibit type I collagen expression in CAFs by dampening the RhoA-YAP axis. This intervention can potentially revert the tumor phenotype to a more immune-infiltrated and softer environment, ultimately enhancing the response to ICI therapy [415]. These findings highlight the potential for repurposing existing drugs to target specific mechanical features of the TME and improve therapeutic outcomes in anti-tumor treatment.

In addition to therapeutic strategies, evaluating these crucial mechanical characteristics within a clinical context is of paramount importance. For instance, in a prospective cohort study involving patients with colorectal cancer, it was revealed that tumor localization on the left side (*p* = 0.0097), advanced stage (*p* < 0.0001), and RAS mutations (*p* = 0.0248) were correlated with elevated tissue stiffness [416]. Although techniques such as AFM that evaluate mechanical cues at the microscale offer high accuracy, their clinical applicability is limited due to low throughput and training-required biopsy, which compromises their efficiency. Encouragingly, a retrospective study demonstrated that a non-invasive macroscale method, amide proton transfer-weighted MRI, could precisely identify a stiff tumor with high sensitivity, specificity, and accuracy rates of 87.1%, 87.5%, and 85.9%, respectively [417]. This study underscores the promising potential of contemporary non-invasive macroscale techniques in discerning the physical attributes of tumor tissues in clinical practice. Moreover, to facilitate clinical applications, it is crucial to develop algorithms that integrate data from both invasive microscopic and non-invasive macroscopic measurement techniques, with the ultimate goal of predicting the mechanical landscape of tumors via non-invasive macroscopic detection alone.

In conclusion, cellular mechanotransduction is an essential factor in both physiological and pathophysiological processes. Investigating cellular mechanotransduction in both non-malignant and tumor tissues provides valuable insights into the underlying mechanisms and potential therapeutic targets associated with mechanical cues within the TME. However, present studies often overlook the intricate interplay between various mechanical cues and between different TME components under stresses. Thus there is a need for future studies to delve into the broader network of interactions among mechanical cues and TME components to deepen our understanding of tumor biology and develop more effective treatment strategies.

## Electronic supplementary material

Below is the link to the electronic supplementary material.

Supplementary Material 1

Supplementary Material 2

Supplementary Material 3

Supplementary Material 4

## Data Availability

No datasets were generated or analysed during the current study.

## Abbreviations

TME: Tumor microenvironment
CAFs: Cancer-associated fibroblasts
ECs: Endothelial cells
MDSCs: Myeloid-derived suppressor cells
Tregs: Regulatory T cells
DCs: Dendritic cells
NK: Natural killer
ECM: Extracellular matrix
PDAC: Pancreatic ductal adenocarcinoma
AFM: Atomic force microscopy
MRI: Magnetic resonance imaging
ULM: Ultrasound localization microscopy
IFP: Interstitial fluid pressure
MRE: Magnetic resonance elastography
SDUV: Shear wave dispersion ultrasound vibrometry
RBC: Red blood cell
CSK: C terminal Src kinase
YAP: Yes-associated protein
TAZ: Transcriptional coactivator with PDZ-binding motif
TRP: Transient receptor potential
IBD: Inflammatory bowel diseases
MscL: Mechanosensitive channel large conductance
MscS: Mechanosensitive channel small conductance
K2P: Two-pore potassium
OSCA/TMEM63: Hyperosmolality-gated calcium-permeable channel families
DEG: Degenerin/epithelial sodium channel
TMC: Transmembrane-like channel
GPCRs: G protein-coupled receptors
RTKs: Receptor tyrosine kinases
SMCs: Smooth muscle cells
Nrf2: Nuclear factor-like 2
IRFs: Interferon regulatory transcription factors
NF-κB: Nuclear factor κB
PDGF: Platelet-derived growth factor
GPAT3: Glycerol-3-phosphate acyltransferase 3
SPRR1B: Small proline-rich protein 1B
NREP: Neuronal regeneration-related protein
CaMKII: Calcium/calmodulin-dependent protein kinase II
NOS: Nitric oxide synthase
PKG: Protein kinase G
FAK: Focal adhesion kinase
LFA1: Lymphocyte function-associated antigen-1
MYH9: Myosin heavy chain 9
MYH10: Myosin heavy chain 10
ATM: Ataxia telangiectasia mutated
ARP2/3: Actin-related protein 2/3
KLF2: Krüppel-like factor 2
KLF4: Krüppel-like factor 4
NDRG1: N-myc downstream-regulated gene 1
VEGFs: Vascular endothelial growth factors
PGC1α: Peroxisome proliferator gamma coactivator-1α
HMGB1: High-mobility group box 1
SASP: Senescence-associated secretory phenotype
BACH1: BTB and CNC homology 1
UCP2: Uncoupling protein 2
AP-1: Activating protein-1
PCSK9: Proprotein convertase subtilisin/kexin type 9
VCAM1: Vascular cell adhesion molecule 1
AMPK: AMP-activated protein kinase
SIRT1: Sirtuin 1
COX2: Cyclo-oxygenase 2
EDN1: Endothelin-1
HIF1α: Hypoxia-inducible factor 1α
TNF-α: Tumor necrosis factor-α
MIF: Macrophage migration inhibitory factor
GSK3: Glycogen synthase kinase 3
BMP: Bone morphogenetic protein
RhoA: Ras homolog family member A
ROCK2: Rho-associated coiled-coil containing protein kinase 2
EMT: Epithelial-to-mesenchymal transition
MMP: Matrix metalloproteinase
TGF: Transforming growth factor
HFpEF: Heart failure with preserved ejection fraction
HSCs: Hepatic stellate cells
LOX: Lysyl oxidases
TG: Transglutaminases
SOX9: SRY-box transcription factor 9
MSCs: Mesenchymal stem cells
PDLs: Periodontal ligaments
RUNX2: Runt-related transcription factor 2
IL-1β: Interleukin 1β
CILK1: Ciliogenesis-associated kinase 1
ANO5: Anoctamin 5
CCL11: C-C motif chemokine ligand 11
S1P: Sphingosine-1-phosphate
S1PR1: Sphingosine-1-phosphate receptor 1
HA: Hyaluronic acid
CCN2: Cellular communication network factor 2
ET-1: Endothelin-1
AGTR1: Angiotensin II receptor 1
OS: Overall survival
PEGPH20: PEGylated recombinant human hyaluronidase
CXCR4: C-X-C chemokine receptor type 4
ADAM10: A disintegrin and metalloprotease 10
KLRG1: Killer cell lectin-like receptor subfamily G member 1
CYR61: Cysteine-rich angiogenic inducer 61
CTGF: Connective tissue growth factor
ANKRD1: Ankyrin repeat domain 1
RAGE: Receptor for advanced glycation end-products
ABCC1: ATP-binding cassette subfamily C member 1
FGF: Fibroblast growth factor
EGF: Epidermal growth factor
ANGPT2: Angiopoietin 2
MT: Microtubule
SRSF1: Serine-arginine-rich splicing factor 1
RANK: Receptor activator of NF-κB
myCAFs: Myofibroblastic CAFs
iCAFs: Inflammatory CAFs
HGF: Hepatocyte growth factor
HCC: Hepatocellular carcinoma
DDR1: Discoidin domain receptor 1
ROS: Reactive oxygen species
CLRs: C-type lectin receptors
MICA: Major histocompatibility complex class I polypeptide-related sequence A
TIMPs: Tissue inhibitors of metalloproteinases
NHE1: Na^+^/H^+^ exchanger 1
PI3K: Phosphatidylinositol 3-kinase
AGEs: Advanced glycation end-products
ICIs: Immune checkpoint inhibitors
ARBs: Angiotensin receptor blockers

## References

[1] Siegel RL, Giaquinto AN, Jemal A. Cancer statistics, 2024. CA Cancer J Clin. 2024;74(1):12–49. 38230766 10.3322/caac.21820

[2] Varmus HE, Weiss RA, Friis RR, Levinson W, Bishop JM. Detection of avian tumor virus-specific nucleotide sequences in avian cell DNAs (reassociation kinetics-RNA tumor viruses-gas antigen-rous sarcoma virus, chick cells). Proc Natl Acad Sci U S A. 1972;69(1):20–4. 4333039 10.1073/pnas.69.1.20 PMC427535

[3] Vogelstein B, Papadopoulos N, Velculescu VE, Zhou S, Diaz LA Jr., Kinzler KW. Cancer genome landscapes. Science. 2013;339(6127):1546–58. 23539594 10.1126/science.1235122 PMC3749880

[4] de Visser KE, Joyce JA. The evolving tumor microenvironment: from cancer initiation to metastatic outgrowth. Cancer Cell. 2023;41(3):374–403. 36917948 10.1016/j.ccell.2023.02.016

[5] Yuan S, Almagro J, Fuchs E. Beyond genetics: driving cancer with the tumour microenvironment behind the wheel. Nat Rev Cancer. 2024;24(4):274–86. 38347101 10.1038/s41568-023-00660-9 PMC11077468

[6] Chhabra Y, Weeraratna AT. Fibroblasts in cancer: Unity in heterogeneity. Cell. 2023;186(8):1580–609. 37059066 10.1016/j.cell.2023.03.016 PMC11422789

[7] Zhang L, Xu J, Zhou S, Yao F, Zhang R, You W, et al. Endothelial DGKG promotes tumor angiogenesis and immune evasion in hepatocellular carcinoma. J Hepatol. 2024;80(1):82–98. 37838036 10.1016/j.jhep.2023.10.006

[8] Jiang Z, Zhou J, Li L, Liao S, He J, Zhou S, et al. Pericytes in the tumor microenvironment. Cancer Lett. 2023;556:216074. 36682706 10.1016/j.canlet.2023.216074

[9] Song Y, Na H, Lee SE, Kim YM, Moon J, Nam TW, et al. Dysfunctional adipocytes promote tumor progression through YAP/TAZ-dependent cancer-associated adipocyte transformation. Nat Commun. 2024;15(1):4052. 38744820 10.1038/s41467-024-48179-3 PMC11094189

[10] Khanmammadova N, Islam S, Sharma P, Amit M. Neuro-immune interactions and immuno-oncology. Trends Cancer. 2023;9(8):636–49. 37258398 10.1016/j.trecan.2023.05.002 PMC10524972

[11] Wu Y, Yi M, Niu M, Mei Q, Wu K. Myeloid-derived suppressor cells: an emerging target for anticancer immunotherapy. Mol Cancer. 2022;21(1):184. 36163047 10.1186/s12943-022-01657-y PMC9513992

[12] Philip M, Schietinger A. CD8(+) T cell differentiation and dysfunction in cancer. Nat Rev Immunol. 2022;22(4):209–23. 34253904 10.1038/s41577-021-00574-3 PMC9792152

[13] Cheng J, Yan J, Liu Y, Shi J, Wang H, Zhou H, et al. Cancer-cell-derived fumarate suppresses the anti-tumor capacity of CD8(+) T cells in the tumor microenvironment. Cell Metab. 2023;35(6):961–e7810. 37178684 10.1016/j.cmet.2023.04.017

[14] Park J, Hsueh PC, Li Z, Ho PC. Microenvironment-driven metabolic adaptations guiding CD8(+) T cell anti-tumor immunity. Immunity. 2023;56(1):32–42. 36630916 10.1016/j.immuni.2022.12.008

[15] van der Leun AM, Thommen DS, Schumacher TN. CD8(+) T cell states in human cancer: insights from single-cell analysis. Nat Rev Cancer. 2020;20(4):218–32. 32024970 10.1038/s41568-019-0235-4 PMC7115982

[16] Lei X, de Groot DC, Welters MJP, de Wit T, Schrama E, van Eenennaam H, et al. CD4(+) T cells produce IFN-I to license cDC1s for induction of cytotoxic T-cell activity in human tumors. Cell Mol Immunol. 2024;21(4):374–92. 38383773 10.1038/s41423-024-01133-1 PMC10978876

[17] Arora J, Ayyappan S, Yin C, Smith BJ, Lemke-Miltner CD, Wang Z, et al. T-cell help in the tumor microenvironment enhances rituximab-mediated NK-cell ADCC. Blood. 2024;143(18):1816–24. 38457360 10.1182/blood.2023023370 PMC11076912

[18] Lei X, Khatri I, de Wit T, de Rink I, Nieuwland M, Kerkhoven R, et al. CD4(+) helper T cells endow cDC1 with cancer-impeding functions in the human tumor micro-environment. Nat Commun. 2023;14(1):217. 36639382 10.1038/s41467-022-35615-5 PMC9839676

[19] Luo Y, Xia Y, Liu D, Li X, Li H, Liu J et al. Neoadjuvant PARPi or chemotherapy in ovarian cancer informs targeting effector Treg cells for homologous-recombination-deficient tumors. Cell. 2024. 10.1016/j.cell.2024.06.013 38971151

[20] You S, Li S, Zeng L, Song J, Li Z, Li W et al. Lymphatic-localized Treg-mregDC crosstalk limits antigen trafficking and restrains anti-tumor immunity. Cancer Cell. 2024. 10.1016/j.ccell.2024.06.014 39029466

[21] Downs-Canner SM, Meier J, Vincent BG, Serody JS. B cell function in the Tumor Microenvironment. Annu Rev Immunol. 2022;40:169–93. 35044794 10.1146/annurev-immunol-101220-015603

[22] Maas RR, Soukup K, Fournier N, Massara M, Galland S, Kornete M, et al. The local microenvironment drives activation of neutrophils in human brain tumors. Cell. 2023;186(21):4546–e6627. 37769657 10.1016/j.cell.2023.08.043

[23] Friedrich M, Hahn M, Michel J, Sankowski R, Kilian M, Kehl N, et al. Dysfunctional dendritic cells limit antigen-specific T cell response in glioma. Neuro Oncol. 2023;25(2):263–76. 35609569 10.1093/neuonc/noac138 PMC9925697

[24] Zhou Y, Cheng L, Liu L, Li X. NK cells are never alone: crosstalk and communication in tumour microenvironments. Mol Cancer. 2023;22(1):34. 36797782 10.1186/s12943-023-01737-7 PMC9933398

[25] Huang J, Zhang L, Wan D, Zhou L, Zheng S, Lin S, et al. Extracellular matrix and its therapeutic potential for cancer treatment. Signal Transduct Target Ther. 2021;6(1):153. 33888679 10.1038/s41392-021-00544-0 PMC8062524

[26] Vitale I, Shema E, Loi S, Galluzzi L. Intratumoral heterogeneity in cancer progression and response to immunotherapy. Nat Med. 2021;27(2):212–24. 33574607 10.1038/s41591-021-01233-9

[27] Mantovani A, Allavena P, Marchesi F, Garlanda C. Macrophages as tools and targets in cancer therapy. Nat Rev Drug Discov. 2022;21(11):799–820. 35974096 10.1038/s41573-022-00520-5 PMC9380983

[28] Petroni G, Buqué A, Coussens LM, Galluzzi L. Targeting oncogene and non-oncogene addiction to inflame the tumour microenvironment. Nat Rev Drug Discov. 2022;21(6):440–62. 35292771 10.1038/s41573-022-00415-5

[29] Hosein AN, Brekken RA, Maitra A. Pancreatic cancer stroma: an update on therapeutic targeting strategies. Nat Rev Gastroenterol Hepatol. 2020;17(8):487–505. 32393771 10.1038/s41575-020-0300-1 PMC8284850

[30] LeSavage BL, Zhang D, Huerta-López C, Gilchrist AE, Krajina BA, Karlsson K, et al. Engineered matrices reveal stiffness-mediated chemoresistance in patient-derived pancreatic cancer organoids. Nat Mater. 2024;23(8):1138–49. 38965405 10.1038/s41563-024-01908-x PMC13098013

[31] Martínez-Reyes I, Chandel NS. Cancer metabolism: looking forward. Nat Rev Cancer. 2021;21(10):669–80. 34272515 10.1038/s41568-021-00378-6

[32] Mao X, Xu J, Wang W, Liang C, Hua J, Liu J, et al. Crosstalk between cancer-associated fibroblasts and immune cells in the tumor microenvironment: new findings and future perspectives. Mol Cancer. 2021;20(1):131. 34635121 10.1186/s12943-021-01428-1 PMC8504100

[33] Iskratsch T, Wolfenson H, Sheetz MP. Appreciating force and shape—the rise of mechanotransduction in cell biology. Nat Rev Mol Cell Biol. 2014;15(12):825–33. 25355507 10.1038/nrm3903 PMC9339222

[34] Tschumperlin DJ. Mechanotransduction Compr Physiol. 2011;1(2):1057–73. 23737212 10.1002/cphy.c100016

[35] Hu Y, Li H, Zhang C, Feng J, Wang W, Chen W, et al. DNA-based ForceChrono probes for deciphering single-molecule force dynamics in living cells. Cell. 2024;187(13):3445–e5915. 38838668 10.1016/j.cell.2024.05.008

[36] Firmin J, Ecker N, Rivet Danon D, Ozguc O, Barraud Lange V, Turlier H, et al. Mechanics of human embryo compaction. Nature. 2024;629(8012):646–51. 38693259 10.1038/s41586-024-07351-x

[37] Bergert M, Lembo S, Sharma S, Russo L, Milovanovic D, Gretarsson KH, et al. Cell surface mechanics gate embryonic stem cell differentiation. Cell Stem Cell. 2021;28(2):209–16. e4. 33207217 10.1016/j.stem.2020.10.017 PMC7875094

[38] Solis AG, Bielecki P, Steach HR, Sharma L, Harman CCD, Yun S, et al. Mechanosensation of cyclical force by PIEZO1 is essential for innate immunity. Nature. 2019;573(7772):69–74. 31435009 10.1038/s41586-019-1485-8 PMC6939392

[39] Sladitschek-Martens HL, Guarnieri A, Brumana G, Zanconato F, Battilana G, Xiccato RL, et al. YAP/TAZ activity in stromal cells prevents ageing by controlling cGAS-STING. Nature. 2022;607(7920):790–8. 35768505 10.1038/s41586-022-04924-6 PMC7613988

[40] Yui S, Azzolin L, Maimets M, Pedersen MT, Fordham RP, Hansen SL, et al. YAP/TAZ-Dependent reprogramming of Colonic epithelium links ECM remodeling to tissue regeneration. Cell Stem Cell. 2018;22(1):35–e497. 29249464 10.1016/j.stem.2017.11.001 PMC5766831

[41] Scholz N, Dahse AK, Kemkemer M, Bormann A, Auger GM, Vieira Contreras F, et al. Molecular sensing of mechano- and ligand-dependent adhesion GPCR dissociation. Nature. 2023;615(7954):945–53. 36890234 10.1038/s41586-023-05802-5

[42] Collins JM, Lang A, Parisi C, Moharrer Y, Nijsure MP, Thomas Kim JH, et al. YAP and TAZ couple osteoblast precursor mobilization to angiogenesis and mechanoregulation in murine bone development. Dev Cell. 2024;59(2):211–e275. 38141609 10.1016/j.devcel.2023.11.029 PMC10843704

[43] Long Y, Niu Y, Liang K, Du Y. Mechanical communication in fibrosis progression. Trends Cell Biol. 2022;32(1):70–90. 34810063 10.1016/j.tcb.2021.10.002

[44] Kalli M, Poskus MD, Stylianopoulos T, Zervantonakis IK. Beyond matrix stiffness: targeting force-induced cancer drug resistance. Trends Cancer. 2023;9(11):937–54. 37558577 10.1016/j.trecan.2023.07.006 PMC10592424

[45] Cambria E, Coughlin MF, Floryan MA, Offeddu GS, Shelton SE, Kamm RD. Linking cell mechanical memory and cancer metastasis. Nat Rev Cancer. 2024;24(3):216–28. 38238471 10.1038/s41568-023-00656-5 PMC11146605

[46] Tao J, Choudhury MI, Maity D, Kim T, Sun SX, Fan C-M. Mechanical compression creates a quiescent muscle stem cell niche. Commun Biology. 2023;6(1). 10.1038/s42003-023-04411-2 PMC9839757 36639551

[47] Xiao R, Liu J, Xu XZS. Mechanosensitive GPCRs and ion channels in shear stress sensing. Curr Opin Cell Biol. 2023;84:102216. 37595342 10.1016/j.ceb.2023.102216 PMC10528224

[48] Chugh M, Munjal A, Megason SG. Hydrostatic pressure as a driver of cell and tissue morphogenesis. Semin Cell Dev Biol. 2022;131:134–45. 35534334 10.1016/j.semcdb.2022.04.021 PMC9529827

[49] Wang X, Ji L, Wang J, Liu C. Matrix stiffness regulates osteoclast fate through integrin-dependent mechanotransduction. Bioact Mater. 2023;27:138–53. 37064801 10.1016/j.bioactmat.2023.03.014 PMC10090259

[50] Di X, Gao X, Peng L, Ai J, Jin X, Qi S, et al. Cellular mechanotransduction in health and diseases: from molecular mechanism to therapeutic targets. Signal Transduct Target Ther. 2023;8(1):282. 37518181 10.1038/s41392-023-01501-9 PMC10387486

[51] Huang M, Maehara A, Tang D, Zhu J, Wang L, Lv R, et al. Impact of residual stress on coronary plaque stress/strain calculations using optical coherence tomography image-based multi-layer models. Front Cardiovasc Med. 2024;11:1395257. 38725836 10.3389/fcvm.2024.1395257 PMC11079268

[52] Kazemi-Lari MA, Shimkunas R, Jian Z, Hegyi B, Izu L, Shaw JA, et al. Modeling cardiomyocyte mechanics and autoregulation of contractility by mechano-chemo-transduction feedback. iScience. 2022;25(7):104667. 35860762 10.1016/j.isci.2022.104667 PMC9289640

[53] Peng Z, Hao M, Tong H, Yang H, Huang B, Zhang Z, et al. The interactions between integrin α(5)β(1) of liver cancer cells and fibronectin of fibroblasts promote tumor growth and angiogenesis. Int J Biol Sci. 2022;18(13):5019–37. 35982891 10.7150/ijbs.72367 PMC9379399

[54] Kopanska KS, Alcheikh Y, Staneva R, Vignjevic D, Betz T. Tensile forces originating from Cancer spheroids facilitate Tumor Invasion. PLoS ONE. 2016;11(6):e0156442. 27271249 10.1371/journal.pone.0156442 PMC4896628

[55] Sitarska E, Diz-Muñoz A. Pay attention to membrane tension: mechanobiology of the cell surface. Curr Opin Cell Biol. 2020;66:11–8. 32416466 10.1016/j.ceb.2020.04.001 PMC7594640

[56] Shah DU, Reynolds TPS, Ramage MH. The strength of plants: theory and experimental methods to measure the mechanical properties of stems. J Exp Bot. 2017;68(16):4497–516. 28981787 10.1093/jxb/erx245

[57] Savadipour A, Nims RJ, Rashidi N, Garcia-Castorena JM, Tang R, Marushack GK, et al. Membrane stretch as the mechanism of activation of PIEZO1 ion channels in chondrocytes. Proc Natl Acad Sci U S A. 2023;120(30):e2221958120. 37459546 10.1073/pnas.2221958120 PMC10372640

[58] Ren K, Gao J, Han D. AFM Force relaxation curve reveals that the decrease of membrane tension is the essential reason for the Softening of Cancer cells. Front Cell Dev Biol. 2021;9:663021. 34055793 10.3389/fcell.2021.663021 PMC8152666

[59] Honarmandi P, Lee H, Lang MJ, Kamm RD. A microfluidic system with optical laser tweezers to study mechanotransduction and focal adhesion recruitment. Lab Chip. 2011;11(4):684–94. 21152510 10.1039/c0lc00487a

[60] Hochmuth RM. Micropipette aspiration of living cells. J Biomech. 2000;33(1):15–22. 10609514 10.1016/s0021-9290(99)00175-x

[61] Seknazi E, Pokroy B. Residual strain and stress in Biocrystals. Adv Mater. 2018;30(41):e1707263. 29766594 10.1002/adma.201707263

[62] Nia HT, Munn LL, Jain RK. Physical traits of cancer. Science. 2020;370(6516). 10.1126/science.aaz0868 PMC8274378 33122355

[63] Zhang W, Sommer G, Niestrawska JA, Holzapfel GA, Nordsletten D. The effects of viscoelasticity on residual strain in aortic soft tissues. Acta Biomater. 2022;140:398–411. 34823042 10.1016/j.actbio.2021.11.019

[64] Helmlinger G, Netti PA, Lichtenbeld HC, Melder RJ, Jain RK. Solid stress inhibits the growth of multicellular tumor spheroids. Nat Biotechnol. 1997;15(8):778–83. 9255794 10.1038/nbt0897-778

[65] Stylianopoulos T, Martin JD, Chauhan VP, Jain SR, Diop-Frimpong B, Bardeesy N, et al. Causes, consequences, and remedies for growth-induced solid stress in murine and human tumors. Proc Natl Acad Sci U S A. 2012;109(38):15101–8. 22932871 10.1073/pnas.1213353109 PMC3458380

[66] Ciarletta P, Destrade M, Gower AL. On residual stresses and homeostasis: an elastic theory of functional adaptation in living matter. Sci Rep. 2016;6:24390. 27113413 10.1038/srep24390 PMC4845028

[67] Campàs O, Mammoto T, Hasso S, Sperling RA, O’Connell D, Bischof AG, et al. Quantifying cell-generated mechanical forces within living embryonic tissues. Nat Methods. 2014;11(2):183–9. 24317254 10.1038/nmeth.2761 PMC3939080

[68] Grashoff C, Hoffman BD, Brenner MD, Zhou R, Parsons M, Yang MT, et al. Measuring mechanical tension across vinculin reveals regulation of focal adhesion dynamics. Nature. 2010;466(7303):263–6. 20613844 10.1038/nature09198 PMC2901888

[69] Nia HT, Liu H, Seano G, Datta M, Jones D, Rahbari N et al. Solid stress and elastic energy as measures of tumour mechanopathology. Nat Biomed Eng. 2016;1. 10.1038/s41551-016-0004 PMC5621647 28966873

[70] Katritsis D, Kaiktsis L, Chaniotis A, Pantos J, Efstathopoulos EP, Marmarelis V. Wall shear stress: theoretical considerations and methods of measurement. Prog Cardiovasc Dis. 2007;49(5):307–29. 17329179 10.1016/j.pcad.2006.11.001

[71] Papaioannou TG, Stefanadis C. Vascular wall shear stress: basic principles and methods. Hellenic J Cardiol. 2005;46(1):9–15. 15807389

[72] Yzet T, Bouzerar R, Baledent O, Renard C, Lumbala DM, Nguyen-Khac E, et al. Dynamic measurements of total hepatic blood flow with phase contrast MRI. Eur J Radiol. 2010;73(1):119–24. 19008062 10.1016/j.ejrad.2008.09.032

[73] Liebig M, Hassanzada A, Kämmerling M, Genz B, Vollmar B, Abshagen K. Microcirculatory disturbances and cellular changes during progression of hepatic steatosis to liver tumors. Exp Biol Med (Maywood). 2018;243(1):1–12. 29065724 10.1177/1535370217738730 PMC5788156

[74] Errico C, Pierre J, Pezet S, Desailly Y, Lenkei Z, Couture O, et al. Ultrafast ultrasound localization microscopy for deep super-resolution vascular imaging. Nature. 2015;527(7579):499–502. 26607546 10.1038/nature16066

[75] Peeters G, Debbaut C, Cornillie P, De Schryver T, Monbaliu D, Laleman W, et al. A multilevel modeling framework to study hepatic perfusion characteristics in case of liver cirrhosis. J Biomech Eng. 2015;137(5):051007. 25473885 10.1115/1.4029280

[76] Bufi N, Durand-Smet P, Asnacios A. Single-cell mechanics: the parallel plates technique. Methods Cell Biol. 2015;125:187–209. 25640430 10.1016/bs.mcb.2014.11.002

[77] Chivukula VK, Krog BL, Nauseef JT, Henry MD, Vigmostad SC. Alterations in cancer cell mechanical properties after fluid shear stress exposure: a micropipette aspiration study. Cell Health Cytoskelet. 2015;7:25–35. 25908902 10.2147/CHC.S71852 PMC4405123

[78] Heldin CH, Rubin K, Pietras K, Ostman A. High interstitial fluid pressure - an obstacle in cancer therapy. Nat Rev Cancer. 2004;4(10):806–13. 15510161 10.1038/nrc1456

[79] Abraldes JG, Sarlieve P, Tandon P. Measurement of portal pressure. Clin Liver Dis. 2014;18(4):779–92. 25438283 10.1016/j.cld.2014.07.002

[80] Wu HJ, Zhang ZQ, Yu B, Liu S, Qin KR, Zhu L. Pressure activates src-dependent FAK-Akt and ERK1/2 signaling pathways in rat hepatic stellate cells. Cell Physiol Biochem. 2010;26(3):273–80. 20798511 10.1159/000320583

[81] Ozerdem U. Measuring interstitial fluid pressure with fiberoptic pressure transducers. Microvasc Res. 2009;77(2):226–9. 18809414 10.1016/j.mvr.2008.08.002 PMC2649693

[82] Salavati H, Debbaut C, Pullens P, Ceelen W. Interstitial fluid pressure as an emerging biomarker in solid tumors. Biochim Biophys Acta Rev Cancer. 2022;1877(5):188792. 36084861 10.1016/j.bbcan.2022.188792

[83] Wang Y, Lu H, Huang L, Li D, Qiu W, Li L et al. Noninvasive estimation of Tumor interstitial fluid pressure from Subharmonic Scattering of Ultrasound contrast Microbubbles. Biosens (Basel). 2023;13(5). 10.3390/bios13050528 PMC10216054 37232888

[84] Theocharis AD, Skandalis SS, Gialeli C, Karamanos NK. Extracellular matrix structure. Adv Drug Deliv Rev. 2016;97:4–27. 26562801 10.1016/j.addr.2015.11.001

[85] Voutouri C, Mpekris F, Panagi M, Krolak C, Michael C, Martin JD, et al. Ultrasound stiffness and perfusion markers correlate with tumor volume responses to immunotherapy. Acta Biomater. 2023;167:121–34. 37321529 10.1016/j.actbio.2023.06.007

[86] Ahmed R, Ye J, Gerber SA, Linehan DC, Doyley MM. Preclinical imaging using single Track Location Shear Wave Elastography: Monitoring the Progression of Murine pancreatic tumor liver metastasis in vivo. IEEE Trans Med Imaging. 2020;39(7):2426–39. 32012006 10.1109/TMI.2020.2971422 PMC7329602

[87] Long L, Liu M, Deng X, Jin J, Cao M, Zhang J, et al. Tumor stiffness measurement at Multifrequency MR Elastography to Predict Lymphovascular Space Invasion in Endometrial Cancer. Radiology. 2024;311(3):e232242. 38832881 10.1148/radiol.232242

[88] Lehtonen AJ, Arasalo O, Srbova L, Heilala M, Pokki J. Magnetic microrheometry of tumor-relevant stiffness levels and probabilistic quantification of viscoelasticity differences inside 3D cell culture matrices. PLoS ONE. 2023;18(3):e0282511. 36947558 10.1371/journal.pone.0282511 PMC10032533

[89] Klaue D, Seidel R. Torsional stiffness of single superparamagnetic microspheres in an external magnetic field. Phys Rev Lett. 2009;102(2):028302. 19257322 10.1103/PhysRevLett.102.028302

[90] Chaudhuri O, Cooper-White J, Janmey PA, Mooney DJ, Shenoy VB. Effects of extracellular matrix viscoelasticity on cellular behaviour. Nature. 2020;584(7822):535–46. 32848221 10.1038/s41586-020-2612-2 PMC7676152

[91] Huang D, Huang Y, Xiao Y, Yang X, Lin H, Feng G, et al. Viscoelasticity in natural tissues and engineered scaffolds for tissue reconstruction. Acta Biomater. 2019;97:74–92. 31400521 10.1016/j.actbio.2019.08.013

[92] Salbreux G, Charras G, Paluch E. Actin cortex mechanics and cellular morphogenesis. Trends Cell Biol. 2012;22(10):536–45. 22871642 10.1016/j.tcb.2012.07.001

[93] Green RA, Paluch E, Oegema K. Cytokinesis in animal cells. Annu Rev Cell Dev Biol. 2012;28:29–58. 22804577 10.1146/annurev-cellbio-101011-155718

[94] Lekka M, Gnanachandran K, Kubiak A, Zieliński T, Zemła J. Traction force microscopy - measuring the forces exerted by cells. Micron. 2021;150:103138. 34416532 10.1016/j.micron.2021.103138

[95] Hao Y, Cheng S, Tanaka Y, Hosokawa Y, Yalikun Y, Li M. Mechanical properties of single cells: measurement methods and applications. Biotechnol Adv. 2020;45:107648. 33080313 10.1016/j.biotechadv.2020.107648

[96] Jin P, Jan LY, Jan YN. Mechanosensitive Ion channels: structural features relevant to mechanotransduction mechanisms. Annu Rev Neurosci. 2020;43:207–29. 32084327 10.1146/annurev-neuro-070918-050509

[97] Cooper J, Giancotti FG. Integrin signaling in Cancer: mechanotransduction, stemness, epithelial plasticity, and Therapeutic Resistance. Cancer Cell. 2019;35(3):347–67. 30889378 10.1016/j.ccell.2019.01.007 PMC6684107

[98] Ke W, Liao Z, Liang H, Tong B, Song Y, Li G, et al. Stiff substrate induces Nucleus Pulposus Cell Ferroptosis via YAP and N-Cadherin mediated mechanotransduction. Adv Healthc Mater. 2023;12(23):e2300458. 37022980 10.1002/adhm.202300458

[99] Fuentes DE, Butler PJ. Coordinated mechanosensitivity of membrane rafts and focal adhesions. Cell Mol Bioeng. 2012;5(2):143–54. 23487555 10.1007/s12195-012-0225-z PMC3593241

[100] Marullo S, Doly S, Saha K, Enslen H, Scott MGH, Coureuil M. Mechanical GPCR activation by Traction forces exerted on receptor N-Glycans. ACS Pharmacol Transl Sci. 2020;3(2):171–8. 32296760 10.1021/acsptsci.9b00106 PMC7155188

[101] Liu J, Zhao C, Xiao X, Li A, Liu Y, Zhao J, et al. Endothelial discoidin domain receptor 1 senses flow to modulate YAP activation. Nat Commun. 2023;14(1):6457. 37833282 10.1038/s41467-023-42341-z PMC10576099

[102] Zhong G, Su S, Li J, Zhao H, Hu D, Chen J, et al. Activation of Piezo1 promotes osteogenic differentiation of aortic valve interstitial cell through YAP-dependent glutaminolysis. Sci Adv. 2023;9(22):eadg0478. 37267365 10.1126/sciadv.adg0478 PMC10413650

[103] Murthy SE, Dubin AE, Patapoutian A. Piezos thrive under pressure: mechanically activated ion channels in health and disease. Nat Rev Mol Cell Biol. 2017;18(12):771–83. 28974772 10.1038/nrm.2017.92

[104] Szczot M, Liljencrantz J, Ghitani N, Barik A, Lam R, Thompson JH et al. PIEZO2 mediates injury-induced tactile pain in mice and humans. Sci Transl Med. 2018;10(462). 10.1126/scitranslmed.aat9892 PMC6875774 30305456

[105] Zeng WZ, Marshall KL, Min S, Daou I, Chapleau MW, Abboud FM, et al. PIEZOs mediate neuronal sensing of blood pressure and the baroreceptor reflex. Science. 2018;362(6413):464–7. 30361375 10.1126/science.aau6324 PMC6563913

[106] Nonomura K, Lukacs V, Sweet DT, Goddard LM, Kanie A, Whitwam T, et al. Mechanically activated ion channel PIEZO1 is required for lymphatic valve formation. Proc Natl Acad Sci U S A. 2018;115(50):12817–22. 30482854 10.1073/pnas.1817070115 PMC6294938

[107] Faucherre A, Moha Ou Maati H, Nasr N, Pinard A, Theron A, Odelin G, et al. Piezo1 is required for outflow tract and aortic valve development. J Mol Cell Cardiol. 2020;143:51–62. 32251670 10.1016/j.yjmcc.2020.03.013

[108] Kang H, Hong Z, Zhong M, Klomp J, Bayless KJ, Mehta D, et al. Piezo1 mediates angiogenesis through activation of MT1-MMP signaling. Am J Physiol Cell Physiol. 2019;316(1):C92–103. 30427721 10.1152/ajpcell.00346.2018 PMC6383143

[109] Pathak MM, Nourse JL, Tran T, Hwe J, Arulmoli J, Le DT, et al. Stretch-activated ion channel Piezo1 directs lineage choice in human neural stem cells. Proc Natl Acad Sci U S A. 2014;111(45):16148–53. 25349416 10.1073/pnas.1409802111 PMC4234578

[110] Gudipaty SA, Lindblom J, Loftus PD, Redd MJ, Edes K, Davey CF, et al. Mechanical stretch triggers rapid epithelial cell division through Piezo1. Nature. 2017;543(7643):118–21. 28199303 10.1038/nature21407 PMC5334365

[111] Li X, Han L, Nookaew I, Mannen E, Silva MJ, Almeida M et al. Stimulation of Piezo1 by mechanical signals promotes bone anabolism. Elife. 2019;8. 10.7554/eLife.49631 PMC6779475 31588901

[112] Ellefsen KL, Holt JR, Chang AC, Nourse JL, Arulmoli J, Mekhdjian AH, et al. Myosin-II mediated traction forces evoke localized Piezo1-dependent ca(2+) flickers. Commun Biol. 2019;2:298. 31396578 10.1038/s42003-019-0514-3 PMC6685976

[113] Song Y, Li D, Farrelly O, Miles L, Li F, Kim SE, et al. The Mechanosensitive Ion Channel Piezo inhibits Axon Regeneration. Neuron. 2019;102(2):373–. – 89.e6. 30819546 10.1016/j.neuron.2019.01.050 PMC6487666

[114] Ma S, Cahalan S, LaMonte G, Grubaugh ND, Zeng W, Murthy SE, et al. Common PIEZO1 allele in African populations causes RBC Dehydration and attenuates plasmodium infection. Cell. 2018;173(2):443–. – 55.e12. 29576450 10.1016/j.cell.2018.02.047 PMC5889333

[115] Nguetse CN, Purington N, Ebel ER, Shakya B, Tetard M, Kremsner PG, et al. A common polymorphism in the mechanosensitive ion channel PIEZO1 is associated with protection from severe malaria in humans. Proc Natl Acad Sci U S A. 2020;117(16):9074–81. 32265284 10.1073/pnas.1919843117 PMC7183233

[116] Winograd-Katz SE, Fässler R, Geiger B, Legate KR. The integrin adhesome: from genes and proteins to human disease. Nat Rev Mol Cell Biol. 2014;15(4):273–88. 24651544 10.1038/nrm3769

[117] Sun Z, Costell M, Fässler R. Integrin activation by talin, kindlin and mechanical forces. Nat Cell Biol. 2019;21(1):25–31. 30602766 10.1038/s41556-018-0234-9

[118] Horton ER, Byron A, Askari JA, Ng DHJ, Millon-Frémillon A, Robertson J, et al. Definition of a consensus integrin adhesome and its dynamics during adhesion complex assembly and disassembly. Nat Cell Biol. 2015;17(12):1577–87. 26479319 10.1038/ncb3257 PMC4663675

[119] Moreno-Layseca P, Icha J, Hamidi H, Ivaska J. Integrin trafficking in cells and tissues. Nat Cell Biol. 2019;21(2):122–32. 30602723 10.1038/s41556-018-0223-z PMC6597357

[120] Israeli-Rosenberg S, Manso AM, Okada H, Ross RS. Integrins and integrin-associated proteins in the cardiac myocyte. Circ Res. 2014;114(3):572–86. 24481847 10.1161/CIRCRESAHA.114.301275 PMC3975046

[121] Kechagia JZ, Ivaska J, Roca-Cusachs P. Integrins as biomechanical sensors of the microenvironment. Nat Rev Mol Cell Biol. 2019;20(8):457–73. 31182865 10.1038/s41580-019-0134-2

[122] Zhang B, Li X, Zhou X, Lou C, Wang S, Lv H, et al. Magneto-mechanical stimulation modulates osteocyte fate via the ECM-integrin-CSK axis and wnt pathway. iScience. 2023;26(8):107365. 37554458 10.1016/j.isci.2023.107365 PMC10405320

[123] Franklin JM, Wu Z, Guan KL. Insights into recent findings and clinical application of YAP and TAZ in cancer. Nat Rev Cancer. 2023;23(8):512–25. 37308716 10.1038/s41568-023-00579-1

[124] Moya IM, Halder G. Hippo-YAP/TAZ signalling in organ regeneration and regenerative medicine. Nat Rev Mol Cell Biol. 2019;20(4):211–26. 30546055 10.1038/s41580-018-0086-y

[125] Yang S, Huang F, Zhang F, Sheng X, Fan W, Dissanayaka WL. Emerging roles of YAP/TAZ in tooth and surrounding: from Development to Regeneration. Stem Cell Rev Rep. 2023;19(6):1659–75. 37178226 10.1007/s12015-023-10551-z

[126] Liu C, Cheng X, Chen J, Wang Y, Wu X, Tian R, et al. Suppression of YAP/TAZ-Notch1-NICD axis by bromodomain and extraterminal protein inhibition impairs liver regeneration. Theranostics. 2019;9(13):3840–52. 31281517 10.7150/thno.33370 PMC6587347

[127] Koo JH, Plouffe SW, Meng Z, Lee DH, Yang D, Lim DS, et al. Induction of AP-1 by YAP/TAZ contributes to cell proliferation and organ growth. Genes Dev. 2020;34(1–2):72–86. 31831627 10.1101/gad.331546.119 PMC6938666

[128] Driskill JH, Pan D. Control of stem cell renewal and fate by YAP and TAZ. Nat Rev Mol Cell Biol. 2023;24(12):895–911. 37626124 10.1038/s41580-023-00644-5

[129] Shi Q, Gui J, Sun L, Song Y, Na J, Zhang J, et al. Frizzled-9 triggers actin polymerization and activates mechano-transducer YAP to rescue simulated microgravity-induced osteoblast dysfunction. Faseb j. 2023;37(9):e23147. 37585277 10.1096/fj.202300977R

[130] Ji M, Chen D, Shu Y, Dong S, Zhang Z, Zheng H, et al. The role of mechano-regulated YAP/TAZ in erectile dysfunction. Nat Commun. 2023;14(1):3758. 37353497 10.1038/s41467-023-39009-z PMC10290143

[131] Panciera T, Azzolin L, Cordenonsi M, Piccolo S. Mechanobiology of YAP and TAZ in physiology and disease. Nat Rev Mol Cell Biol. 2017;18(12):758–70. 28951564 10.1038/nrm.2017.87 PMC6192510

[132] Levental I, Levental KR, Heberle FA. Lipid rafts: controversies resolved, mysteries remain. Trends Cell Biol. 2020;30(5):341–53. 32302547 10.1016/j.tcb.2020.01.009 PMC7798360

[133] Petersen EN, Chung HW, Nayebosadri A, Hansen SB. Kinetic disruption of lipid rafts is a mechanosensor for phospholipase D. Nat Commun. 2016;7:13873. 27976674 10.1038/ncomms13873 PMC5171650

[134] Zhang C, Zhou T, Chen Z, Yan M, Li B, Lv H, et al. Coupling of integrin α5 to Annexin A2 by Flow drives endothelial activation. Circ Res. 2020;127(8):1074–90. 32673515 10.1161/CIRCRESAHA.120.316857

[135] Gu X, Reagan AM, McClellan ME, Elliott MH. Caveolins and caveolae in ocular physiology and pathophysiology. Prog Retin Eye Res. 2017;56:84–106. 27664379 10.1016/j.preteyeres.2016.09.005 PMC5237608

[136] Das J, Maji S, Agarwal T, Chakraborty S, Maiti TK. Hemodynamic shear stress induces protective autophagy in HeLa cells through lipid raft-mediated mechanotransduction. Clin Exp Metastasis. 2018;35(3):135–48. 29536225 10.1007/s10585-018-9887-9

[137] Das J, Agarwal T, Chakraborty S, Maiti TK. Compressive stress-induced autophagy promotes invasion of HeLa cells by facilitating protein turnover in vitro. Exp Cell Res. 2019;381(2):201–7. 31075254 10.1016/j.yexcr.2019.04.037

[138] Zhang M, Ma Y, Ye X, Zhang N, Pan L, Wang B. TRP (transient receptor potential) ion channel family: structures, biological functions and therapeutic interventions for diseases. Signal Transduct Target Ther. 2023;8(1):261. 37402746 10.1038/s41392-023-01464-x PMC10319900

[139] Kefauver JM, Ward AB, Patapoutian A. Discoveries in structure and physiology of mechanically activated ion channels. Nature. 2020;587(7835):567–76. 33239794 10.1038/s41586-020-2933-1 PMC8477435

[140] De Logu F, Nassini R, Materazzi S, Carvalho Gonçalves M, Nosi D, Rossi Degl’Innocenti D, et al. Schwann cell TRPA1 mediates neuroinflammation that sustains macrophage-dependent neuropathic pain in mice. Nat Commun. 2017;8(1):1887. 29192190 10.1038/s41467-017-01739-2 PMC5709495

[141] Chen Y, Mu J, Zhu M, Mukherjee A, Zhang H. Transient receptor potential channels and inflammatory bowel disease. Front Immunol. 2020;11:180. 32153564 10.3389/fimmu.2020.00180 PMC7044176

[142] Fakih D, Migeon T, Moreau N, Baudouin C, Réaux-Le Goazigo A, Mélik Parsadaniantz S. Transient receptor potential channels: important players in Ocular Pain and Dry Eye Disease. Pharmaceutics. 2022;14(9). 10.3390/pharmaceutics14091859 PMC9506338 36145607

[143] Zhao R, Afthinos A, Zhu T, Mistriotis P, Li Y, Serra SA, et al. Cell sensing and decision-making in confinement: the role of TRPM7 in a tug of war between hydraulic pressure and cross-sectional area. Sci Adv. 2019;5(7):eaaw7243. 31355337 10.1126/sciadv.aaw7243 PMC6656542

[144] Deng Z, Paknejad N, Maksaev G, Sala-Rabanal M, Nichols CG, Hite RK, et al. Cryo-EM and X-ray structures of TRPV4 reveal insight into ion permeation and gating mechanisms. Nat Struct Mol Biol. 2018;25(3):252–60. 29483651 10.1038/s41594-018-0037-5 PMC6252174

[145] Gründer S, Ramírez AO, Jékely G. Neuropeptides and degenerin/epithelial na(+) channels: a relationship from mammals to cnidarians. J Physiol. 2023;601(9):1583–95. 36479972 10.1113/JP282309

[146] Cox CD, Bavi N, Martinac B. Bacterial mechanosensors. Annu Rev Physiol. 2018;80:71–93. 29195054 10.1146/annurev-physiol-021317-121351

[147] Brohawn SG, Su Z, MacKinnon R. Mechanosensitivity is mediated directly by the lipid membrane in TRAAK and TREK1 K + channels. Proc Natl Acad Sci U S A. 2014;111(9):3614–9. 24550493 10.1073/pnas.1320768111 PMC3948252

[148] Zhang M, Shan Y, Cox CD, Pei D. A mechanical-coupling mechanism in OSCA/TMEM63 channel mechanosensitivity. Nat Commun. 2023;14(1):3943. 37402734 10.1038/s41467-023-39688-8 PMC10319725

[149] Yuan F, Yang H, Xue Y, Kong D, Ye R, Li C, et al. OSCA1 mediates osmotic-stress-evoked Ca2 + increases vital for osmosensing in Arabidopsis. Nature. 2014;514(7522):367–71. 25162526 10.1038/nature13593

[150] Li Q, Montell C. Mechanism for food texture preference based on grittiness. Curr Biol. 2021;31(9):1850–e616. 33657409 10.1016/j.cub.2021.02.007 PMC8119346

[151] Du H, Ye C, Wu D, Zang YY, Zhang L, Chen C, et al. The Cation Channel TMEM63B is an Osmosensor required for hearing. Cell Rep. 2020;31(5):107596. 32375046 10.1016/j.celrep.2020.107596

[152] Tábara LC, Al-Salmi F, Maroofian R, Al-Futaisi AM, Al-Murshedi F, Kennedy J, et al. TMEM63C mutations cause mitochondrial morphology defects and underlie hereditary spastic paraplegia. Brain. 2022;145(9):3095–107. 35718349 10.1093/brain/awac123 PMC9473353

[153] Yan H, Helman G, Murthy SE, Ji H, Crawford J, Kubisiak T, et al. Heterozygous variants in the Mechanosensitive Ion Channel TMEM63A Result in transient hypomyelination during infancy. Am J Hum Genet. 2019;105(5):996–1004. 31587869 10.1016/j.ajhg.2019.09.011 PMC6848986

[154] Ballesteros A, Swartz KJ. Regulation of membrane homeostasis by TMC1 mechanoelectrical transduction channels is essential for hearing. Sci Adv. 2022;8(31):eabm5550. 35921424 10.1126/sciadv.abm5550 PMC9348795

[155] Böl M, Kohn S, Leichsenring K, Morales-Orcajo E, Ehret AE. On multiscale tension-compression asymmetry in skeletal muscle. Acta Biomater. 2022;144:210–20. 35339701 10.1016/j.actbio.2022.03.034

[156] Rosenfeld D, Landau S, Shandalov Y, Raindel N, Freiman A, Shor E, et al. Morphogenesis of 3D vascular networks is regulated by tensile forces. Proc Natl Acad Sci U S A. 2016;113(12):3215–20. 26951667 10.1073/pnas.1522273113 PMC4812755

[157] Loverde JR, Tolentino RE, Soteropoulos P, Pfister BJ. Biomechanical forces regulate gene transcription during Stretch-mediated growth of mammalian neurons. Front Neurosci. 2020;14:600136. 33408609 10.3389/fnins.2020.600136 PMC7780124

[158] Gao X, Wei T, Liao B, Ai J, Zhou L, Gong L, et al. Physiological stretch induced proliferation of human urothelial cells via integrin α6-FAK signaling pathway. Neurourol Urodyn. 2018;37(7):2114–20. 29953644 10.1002/nau.23572

[159] Basu R, Huse M. Mechanical communication at the immunological synapse. Trends Cell Biol. 2017;27(4):241–54. 27986534 10.1016/j.tcb.2016.10.005 PMC5367987

[160] Opplert J, Babault N. Acute effects of Dynamic stretching on muscle flexibility and performance: an analysis of the current literature. Sports Med. 2018;48(2):299–325. 29063454 10.1007/s40279-017-0797-9

[161] Plocienniczak M, Tracy LF. Muscle tension Dysphonia. JAMA Otolaryngol Head Neck Surg. 2022;148(9):895. 35900756 10.1001/jamaoto.2022.1944

[162] Rysä J, Tokola H, Ruskoaho H. Mechanical stretch induced transcriptomic profiles in cardiac myocytes. Sci Rep. 2018;8(1):4733. 29549296 10.1038/s41598-018-23042-w PMC5856749

[163] Bloomekatz J, Diaz JT, Yelon D, Chi NC. Cardiac morphogenesis: crowding and tension resolved through Social Distancing. Dev Cell. 2021;56(2):159–60. 33497621 10.1016/j.devcel.2021.01.001 PMC8552508

[164] Priya R, Allanki S, Gentile A, Mansingh S, Uribe V, Maischein HM, et al. Tension heterogeneity directs form and fate to pattern the myocardial wall. Nature. 2020;588(7836):130–4. 33208950 10.1038/s41586-020-2946-9

[165] Medvedev RY, Afolabi SO, Turner DGP, Glukhov AV. Mechanisms of stretch-induced electro-anatomical remodeling and atrial arrhythmogenesis. J Mol Cell Cardiol. 2024;193:11–24. 38797242 10.1016/j.yjmcc.2024.05.011 PMC11260238

[166] Veeraval L, O’Leary CJ, Cooper HM. Adherens junctions: guardians of cortical development. Front Cell Dev Biol. 2020;8:6. 32117958 10.3389/fcell.2020.00006 PMC7025593

[167] Yang S, Wang M, Tian D, Zhang X, Cui K, Lü S, et al. DNA-functionalized artificial mechanoreceptor for de novo force-responsive signaling. Nat Chem Biol. 2024;20(8):1066–77. 38448735 10.1038/s41589-024-01572-x

[168] Mascharak S, desJardins-Park HE, Davitt MF, Griffin M, Borrelli MR, Moore AL et al. Preventing Engrailed-1 activation in fibroblasts yields wound regeneration without scarring. Science. 2021;372(6540). 10.1126/science.aba2374 PMC9008875 33888614

[169] . DF-1.7%âãÏÓ 1 0 obj </Image/Height 45/Filter/FlateDecode/Type/XObject/Width 228/Length 32/BitsPerComponent 8 > > stream xœíÁ p.

[170] Wang P, Jia Y, Liu T, Jan YN, Zhang W. Visceral mechano-sensing neurons Control Drosophila feeding by using Piezo as a Sensor. Neuron. 2020;108(4):640–e504. 32910893 10.1016/j.neuron.2020.08.017 PMC8386590

[171] Liu CSC, Mandal T, Biswas P, Hoque MA, Bandopadhyay P, Sinha BP et al. Piezo1 mechanosensing regulates integrin-dependent chemotactic migration in human T cells. Elife. 2024;12. 10.7554/eLife.91903 PMC10942591 38393325

[172] Chicurel ME, Singer RH, Meyer CJ, Ingber DE. Integrin binding and mechanical tension induce movement of mRNA and ribosomes to focal adhesions. Nature. 1998;392(6677):730–3. 9565036 10.1038/33719

[173] Bastianello G, Porcella G, Beznoussenko GV, Kidiyoor G, Ascione F, Li Q, et al. Cell stretching activates an ATM mechano-transduction pathway that remodels cytoskeleton and chromatin. Cell Rep. 2023;42(12):113555. 38088930 10.1016/j.celrep.2023.113555

[174] Kirkland NJ, Yuen AC, Tozluoglu M, Hui N, Paluch EK, Mao Y. Tissue mechanics regulate Mitotic Nuclear Dynamics during Epithelial Development. Curr Biol. 2020;30(13):2419–e324. 32413305 10.1016/j.cub.2020.04.041 PMC7342018

[175] Chang W, Wang Y, Luxton GWG, Östlund C, Worman HJ, Gundersen GG. Imbalanced nucleocytoskeletal connections create common polarity defects in progeria and physiological aging. Proc Natl Acad Sci U S A. 2019;116(9):3578–83. 30808750 10.1073/pnas.1809683116 PMC6397528

[176] Seelbinder B, Ghosh S, Schneider SE, Scott AK, Berman AG, Goergen CJ, et al. Nuclear deformation guides chromatin reorganization in cardiac development and disease. Nat Biomed Eng. 2021;5(12):1500–16. 34857921 10.1038/s41551-021-00823-9 PMC9300284

[177] Dantas M, Oliveira A, Aguiar P, Maiato H, Ferreira JG. Nuclear tension controls mitotic entry by regulating cyclin B1 nuclear translocation. J Cell Biol. 2022;221(12). 10.1083/jcb.202205051 PMC9565158 36222828

[178] Nava MM, Miroshnikova YA, Biggs LC, Whitefield DB, Metge F, Boucas J, et al. Heterochromatin-Driven Nuclear Softening protects the genome against mechanical stress-Induced damage. Cell. 2020;181(4):800–e1722. 32302590 10.1016/j.cell.2020.03.052 PMC7237863

[179] Maurya MR, Gupta S, Li JY, Ajami NE, Chen ZB, Shyy JY et al. Longitudinal shear stress response in human endothelial cells to atheroprone and atheroprotective conditions. Proc Natl Acad Sci U S A. 2021;118(4). 10.1073/pnas.2023236118 PMC7848718 33468662

[180] Zhang G, Qin Q, Zhang C, Sun X, Kazama K, Yi B, et al. NDRG1 signaling is essential for endothelial inflammation and vascular remodeling. Circ Res. 2023;132(3):306–19. 36562299 10.1161/CIRCRESAHA.122.321837 PMC9898177

[181] Ranade SS, Qiu Z, Woo SH, Hur SS, Murthy SE, Cahalan SM, et al. Piezo1, a mechanically activated ion channel, is required for vascular development in mice. Proc Natl Acad Sci U S A. 2014;111(28):10347–52. 24958852 10.1073/pnas.1409233111 PMC4104881

[182] Zainal Abidin NA, Timofeeva M, Szydzik C, Akbaridoust F, Lav C, Marusic I, et al. A microfluidic method to investigate platelet mechanotransduction under extensional strain. Res Pract Thromb Haemost. 2023;7(1):100037. 36846647 10.1016/j.rpth.2023.100037 PMC9944983

[183] Chiu JJ, Chien S. Effects of disturbed flow on vascular endothelium: pathophysiological basis and clinical perspectives. Physiol Rev. 2011;91(1):327–87. 21248169 10.1152/physrev.00047.2009 PMC3844671

[184] Ding Z, Pothineni NVK, Goel A, Lüscher TF, Mehta JL. PCSK9 and inflammation: role of shear stress, pro-inflammatory cytokines, and LOX-1. Cardiovasc Res. 2020;116(5):908–15. 31746997 10.1093/cvr/cvz313

[185] Kant S, Tran KV, Kvandova M, Caliz AD, Yoo HJ, Learnard H, et al. PGC1α regulates the endothelial response to fluid shear stress via Telomerase Reverse Transcriptase Control of Heme Oxygenase-1. Arterioscler Thromb Vasc Biol. 2022;42(1):19–34. 34789002 10.1161/ATVBAHA.121.317066 PMC8702461

[186] Tsao PS, Buitrago R, Chan JR, Cooke JP. Fluid flow inhibits endothelial adhesiveness. Nitric oxide and transcriptional regulation of VCAM-1. Circulation. 1996;94(7):1682–9. 8840861 10.1161/01.cir.94.7.1682

[187] Feinberg MW, Moore KJ. MicroRNA regulation of atherosclerosis. Circ Res. 2016;118(4):703–20. 26892968 10.1161/CIRCRESAHA.115.306300 PMC4762069

[188] Meng Q, Pu L, Qi M, Li S, Sun B, Wang Y, et al. Laminar shear stress inhibits inflammation by activating autophagy in human aortic endothelial cells through HMGB1 nuclear translocation. Commun Biol. 2022;5(1):425. 35523945 10.1038/s42003-022-03392-y PMC9076621

[189] Davies PF, Remuzzi A, Gordon EJ, Dewey CF Jr., Gimbrone MA. Jr. Turbulent fluid shear stress induces vascular endothelial cell turnover in vitro. Proc Natl Acad Sci U S A. 1986;83(7):2114–7. 3457378 10.1073/pnas.83.7.2114 PMC323241

[190] Yamawaki H, Pan S, Lee RT, Berk BC. Fluid shear stress inhibits vascular inflammation by decreasing thioredoxin-interacting protein in endothelial cells. J Clin Invest. 2005;115(3):733–8. 15696199 10.1172/JCI200523001 PMC546457

[191] Jia M, Li Q, Guo J, Shi W, Zhu L, Huang Y, et al. Deletion of BACH1 attenuates atherosclerosis by reducing endothelial inflammation. Circ Res. 2022;130(7):1038–55. 35196865 10.1161/CIRCRESAHA.121.319540

[192] Luo JY, Cheng CK, He L, Pu Y, Zhang Y, Lin X, et al. Endothelial UCP2 is a mechanosensitive suppressor of atherosclerosis. Circ Res. 2022;131(5):424–41. 35899624 10.1161/CIRCRESAHA.122.321187 PMC9390236

[193] Okamoto T, Park EJ, Kawamoto E, Usuda H, Wada K, Taguchi A, et al. Endothelial connexin-integrin crosstalk in vascular inflammation. Biochim Biophys Acta Mol Basis Dis. 2021;1867(9):166168. 33991620 10.1016/j.bbadis.2021.166168

[194] Tsao PS, Lewis NP, Alpert S, Cooke JP. Exposure to shear stress alters endothelial adhesiveness. Role of nitric oxide. Circulation. 1995;92(12):3513–9. 8521574 10.1161/01.cir.92.12.3513

[195] Claude-Taupin A, Isnard P, Bagattin A, Kuperwasser N, Roccio F, Ruscica B, et al. The AMPK-Sirtuin 1-YAP axis is regulated by fluid flow intensity and controls autophagy flux in kidney epithelial cells. Nat Commun. 2023;14(1):8056. 38052799 10.1038/s41467-023-43775-1 PMC10698145

[196] Miceli C, Roccio F, Penalva-Mousset L, Burtin M, Leroy C, Nemazanyy I, et al. The primary cilium and lipophagy translate mechanical forces to direct metabolic adaptation of kidney epithelial cells. Nat Cell Biol. 2020;22(9):1091–102. 32868900 10.1038/s41556-020-0566-0

[197] Kriz W, Lemley KV. Potential relevance of shear stress for slit diaphragm and podocyte function. Kidney Int. 2017;91(6):1283–6. 28501303 10.1016/j.kint.2017.02.032

[198] Dunne OM, Martin SL, Sergeant GP, McAuley DF, O’Kane CM, Button B, et al. TRPV2 modulates mechanically Induced ATP Release from Human bronchial epithelial cells. Respir Res. 2024;25(1):188. 38678280 10.1186/s12931-024-02807-0 PMC11056070

[199] Henstock JR, Rotherham M, Rose JB, El Haj AJ. Cyclic hydrostatic pressure stimulates enhanced bone development in the foetal chick femur in vitro. Bone. 2013;53(2):468–77. 23333177 10.1016/j.bone.2013.01.010

[200] Alasaadi DN, Alvizi L, Hartmann J, Stillman N, Moghe P, Hiiragi T, et al. Competence for neural crest induction is controlled by hydrostatic pressure through Yap. Nat Cell Biol. 2024;26(4):530–41. 38499770 10.1038/s41556-024-01378-y PMC11021196

[201] Stewart MP, Helenius J, Toyoda Y, Ramanathan SP, Muller DJ, Hyman AA. Hydrostatic pressure and the actomyosin cortex drive mitotic cell rounding. Nature. 2011;469(7329):226–30. 21196934 10.1038/nature09642

[202] Stavenschi E, Corrigan MA, Johnson GP, Riffault M, Hoey DA. Physiological cyclic hydrostatic pressure induces osteogenic lineage commitment of human bone marrow stem cells: a systematic study. Stem Cell Res Ther. 2018;9(1):276. 30359324 10.1186/s13287-018-1025-8 PMC6203194

[203] Yamaguchi T, Hashiguchi K, Katsuki S, Iwamoto W, Tsuruhara S, Terada S. Activation of the intrinsic and extrinsic pathways in high pressure-induced apoptosis of murine erythroleukemia cells. Cell Mol Biol Lett. 2008;13(1):49–57. 17952376 10.2478/s11658-007-0034-x PMC6275616

[204] Waletzko-Hellwig J, Sass JO, Bader R, Frerich B, Dau M. Evaluation of Integrity of Allogeneic Bone processed with high hydrostatic pressure: a Pilot Animal Study. Biomater Res. 2024;28:0067. 39148817 10.34133/bmr.0067 PMC11325089

[205] Li X, Xue YM, Guo HM, Deng CY, Peng DW, Yang H, et al. High hydrostatic pressure induces atrial electrical remodeling through upregulation of inflammatory cytokines. Life Sci. 2020;242:117209. 31870776 10.1016/j.lfs.2019.117209

[206] Marchioni A, Tonelli R, Cerri S, Castaniere I, Andrisani D, Gozzi F et al. Pulmonary Stretch and Lung Mechanotransduction: implications for progression in the fibrotic lung. Int J Mol Sci. 2021;22(12). 10.3390/ijms22126443 PMC8234308 34208586

[207] Karsdal MA, Nielsen SH, Leeming DJ, Langholm LL, Nielsen MJ, Manon-Jensen T, et al. The good and the bad collagens of fibrosis - their role in signaling and organ function. Adv Drug Deliv Rev. 2017;121:43–56. 28736303 10.1016/j.addr.2017.07.014

[208] Kalappurakkal JM, Anilkumar AA, Patra C, van Zanten TS, Sheetz MP, Mayor S. Integrin mechano-chemical signaling generates plasma membrane nanodomains that promote cell spreading. Cell. 2019;177(7):1738–e5623. 31104842 10.1016/j.cell.2019.04.037 PMC6879320

[209] Maurer M, Lammerding J. The Driving Force: Nuclear Mechanotransduction in Cellular function, Fate, and Disease. Annu Rev Biomed Eng. 2019;21:443–68. 30916994 10.1146/annurev-bioeng-060418-052139 PMC6815102

[210] Weickenmeier J, de Rooij R, Budday S, Steinmann P, Ovaert TC, Kuhl E. Brain stiffness increases with myelin content. Acta Biomater. 2016;42:265–72. 27475531 10.1016/j.actbio.2016.07.040

[211] Hu J, Chen Q, Zhu H, Hou L, Liu W, Yang Q, et al. Microglial Piezo1 senses Aβ fibril stiffness to restrict Alzheimer’s disease. Neuron. 2023;111(1):15–e298. 36368316 10.1016/j.neuron.2022.10.021

[212] Hussien AA, Niederoest B, Bollhalder M, Goedecke N, Snedeker JG. The stiffness-sensitive transcriptome of human tendon stromal cells. Adv Healthc Mater. 2023;12(7):e2101216. 36509005 10.1002/adhm.202101216 PMC11468939

[213] Nolte M, Margadant C. Controlling immunity and inflammation through integrin-dependent regulation of TGF-β. Trends Cell Biol. 2020;30(1):49–59. 31744661 10.1016/j.tcb.2019.10.002

[214] Przybyla L, Lakins JN, Weaver VM. Tissue mechanics orchestrate wnt-dependent human embryonic stem cell differentiation. Cell Stem Cell. 2016;19(4):462–75. 27452175 10.1016/j.stem.2016.06.018 PMC5336327

[215] Sales A, Khodr V, Machillot P, Chaar L, Fourel L, Guevara-Garcia A, et al. Differential bioactivity of four BMP-family members as function of biomaterial stiffness. Biomaterials. 2022;281:121363. 35063741 10.1016/j.biomaterials.2022.121363 PMC7613911

[216] Sun Q, Pei F, Zhang M, Zhang B, Jin Y, Zhao Z, et al. Curved Nanofiber Network induces Cellular Bridge formation to promote stem cell mechanotransduction. Adv Sci (Weinh). 2023;10(3):e2204479. 36382560 10.1002/advs.202204479 PMC9875655

[217] Barriga EH, Franze K, Charras G, Mayor R. Tissue stiffening coordinates morphogenesis by triggering collective cell migration in vivo. Nature. 2018;554(7693):523–7. 29443958 10.1038/nature25742 PMC6013044

[218] Geiger B, Bershadsky A, Pankov R, Yamada KM. Transmembrane crosstalk between the extracellular matrix–cytoskeleton crosstalk. Nat Rev Mol Cell Biol. 2001;2(11):793–805. 11715046 10.1038/35099066

[219] Dityatev A, Schachner M. Extracellular matrix molecules and synaptic plasticity. Nat Rev Neurosci. 2003;4(6):456–68. 12778118 10.1038/nrn1115

[220] Georges PC, Janmey PA. Cell type-specific response to growth on soft materials. J Appl Physiol (1985). 2005;98(4):1547–53. 15772065 10.1152/japplphysiol.01121.2004

[221] Flanagan LA, Ju YE, Marg B, Osterfield M, Janmey PA. Neurite branching on deformable substrates. NeuroReport. 2002;13(18):2411–5. 12499839 10.1097/01.wnr.0000048003.96487.97 PMC2408859

[222] Lei M, Harn HI, Li Q, Jiang J, Wu W, Zhou W, et al. The mechano-chemical circuit drives skin organoid self-organization. Proc Natl Acad Sci U S A. 2023;120(36):e2221982120. 37643215 10.1073/pnas.2221982120 PMC10483620

[223] Klingberg F, Chow ML, Koehler A, Boo S, Buscemi L, Quinn TM, et al. Prestress in the extracellular matrix sensitizes latent TGF-β1 for activation. J Cell Biol. 2014;207(2):283–97. 25332161 10.1083/jcb.201402006 PMC4210443

[224] Faleeva M, Ahmad S, Theofilatos K, Lynham S, Watson G, Whitehead M, et al. Sox9 accelerates vascular aging by regulating Extracellular Matrix composition and stiffness. Circ Res. 2024;134(3):307–24. 38179698 10.1161/CIRCRESAHA.123.323365 PMC10826924

[225] Zhang W, Zhang S, Zhang W, Yue Y, Qian W, Wang Z. Matrix stiffness and its influence on pancreatic diseases. Biochim Biophys Acta Rev Cancer. 2021;1876(1):188583. 34139274 10.1016/j.bbcan.2021.188583

[226] Querejeta R, López B, González A, Sánchez E, Larman M, Martínez Ubago JL, et al. Increased collagen type I synthesis in patients with heart failure of hypertensive origin: relation to myocardial fibrosis. Circulation. 2004;110(10):1263–8. 15313958 10.1161/01.CIR.0000140973.60992.9A

[227] López B, González A, Querejeta R, Larman M, Rábago G, Díez J. Association of cardiotrophin-1 with myocardial fibrosis in hypertensive patients with heart failure. Hypertension. 2014;63(3):483–9. 24366078 10.1161/HYPERTENSIONAHA.113.02654

[228] Martos R, Baugh J, Ledwidge M, O’Loughlin C, Conlon C, Patle A, et al. Diastolic heart failure: evidence of increased myocardial collagen turnover linked to diastolic dysfunction. Circulation. 2007;115(7):888–95. 17283265 10.1161/CIRCULATIONAHA.106.638569

[229] López B, González A, Querejeta R, Larman M, Díez J. Alterations in the pattern of collagen deposition may contribute to the deterioration of systolic function in hypertensive patients with heart failure. J Am Coll Cardiol. 2006;48(1):89–96. 16814653 10.1016/j.jacc.2006.01.077

[230] Zile MR, Baicu CF, Ikonomidis JS, Stroud RE, Nietert PJ, Bradshaw AD, et al. Myocardial stiffness in patients with heart failure and a preserved ejection fraction: contributions of collagen and titin. Circulation. 2015;131(14):1247–59. 25637629 10.1161/CIRCULATIONAHA.114.013215 PMC4390480

[231] Qian W, Hadi T, Silvestro M, Ma X, Rivera CF, Bajpai A, et al. Microskeletal stiffness promotes aortic aneurysm by sustaining pathological vascular smooth muscle cell mechanosensation via Piezo1. Nat Commun. 2022;13(1):512. 35082286 10.1038/s41467-021-27874-5 PMC8791986

[232] Kisseleva T, Brenner D. Molecular and cellular mechanisms of liver fibrosis and its regression. Nat Rev Gastroenterol Hepatol. 2021;18(3):151–66. 33128017 10.1038/s41575-020-00372-7

[233] Bataller R, Brenner DA. Liver fibrosis. J Clin Invest. 2005;115(2):209–18. 15690074 10.1172/JCI24282 PMC546435

[234] Qiu S, Zhao X, Chen J, Zeng J, Chen S, Chen L, et al. Characterizing viscoelastic properties of breast cancer tissue in a mouse model using indentation. J Biomech. 2018;69:81–9. 29361276 10.1016/j.jbiomech.2018.01.007

[235] Perepelyuk M, Chin L, Cao X, van Oosten A, Shenoy VB, Janmey PA, et al. Normal and fibrotic rat livers demonstrate shear strain softening and Compression Stiffening: a model for soft tissue mechanics. PLoS ONE. 2016;11(1):e0146588. 26735954 10.1371/journal.pone.0146588 PMC4703410

[236] Reihsner R, Menzel EJ. Two-dimensional stress-relaxation behavior of human skin as influenced by non-enzymatic glycation and the inhibitory agent aminoguanidine. J Biomech. 1998;31(11):985–93. 9880055 10.1016/s0021-9290(98)00088-8

[237] Geerligs M, Peters GW, Ackermans PA, Oomens CW, Baaijens FP. Linear viscoelastic behavior of subcutaneous adipose tissue. Biorheology. 2008;45(6):677–88. 19065014

[238] Augat P, Schorlemmer S. The role of cortical bone and its microstructure in bone strength. Age Ageing. 2006;35(Suppl 2):ii27–31. 16926200 10.1093/ageing/afl081

[239] Fan F, Cai X, Follet H, Peyrin F, Laugier P, Niu H, et al. Cortical bone viscoelastic damping assessed with resonant ultrasound spectroscopy reflects porosity and mineral content. J Mech Behav Biomed Mater. 2021;117:104388. 33636678 10.1016/j.jmbbm.2021.104388

[240] Dabrowska S, Ekiert-Radecka M, Karbowniczek J, Weglarz WP, Heljak M, Lojkowski M, et al. Calcification alters the viscoelastic properties of tendon fascicle bundles depending on matrix content. Acta Biomater. 2023;166:360–74. 37172636 10.1016/j.actbio.2023.05.010

[241] Zhang JJ, Li X, Tian Y, Zou JK, Gan D, Deng DK, et al. Harnessing mechanical stress with viscoelastic biomaterials for Periodontal Ligament Regeneration. Adv Sci (Weinh). 2024;11(18):e2309562. 38460171 10.1002/advs.202309562 PMC11095218

[242] Hodgkinson T, Kelly DC, Curtin CM, O’Brien FJ. Mechanosignalling in cartilage: an emerging target for the treatment of osteoarthritis. Nat Rev Rheumatol. 2022;18(2):67–84. 34934171 10.1038/s41584-021-00724-w

[243] Chaudhuri O, Gu L, Klumpers D, Darnell M, Bencherif SA, Weaver JC, et al. Hydrogels with tunable stress relaxation regulate stem cell fate and activity. Nat Mater. 2016;15(3):326–34. 26618884 10.1038/nmat4489 PMC4767627

[244] Domingues MM, Carvalho FA, Santos NC. Nanomechanics of blood clot and Thrombus formation. Annu Rev Biophys. 2022;51:201–21. 34990221 10.1146/annurev-biophys-111821-072110

[245] Budday S, Sommer G, Birkl C, Langkammer C, Haybaeck J, Kohnert J, et al. Mechanical characterization of human brain tissue. Acta Biomater. 2017;48:319–40. 27989920 10.1016/j.actbio.2016.10.036

[246] Budday S, Sommer G, Holzapfel GA, Steinmann P, Kuhl E. Viscoelastic parameter identification of human brain tissue. J Mech Behav Biomed Mater. 2017;74:463–76. 28756040 10.1016/j.jmbbm.2017.07.014

[247] Elosegui-Artola A. The extracellular matrix viscoelasticity as a regulator of cell and tissue dynamics. Curr Opin Cell Biol. 2021;72:10–8. 33993058 10.1016/j.ceb.2021.04.002

[248] Cameron AR, Frith JE, Cooper-White JJ. The influence of substrate creep on mesenchymal stem cell behaviour and phenotype. Biomaterials. 2011;32(26):5979–93. 21621838 10.1016/j.biomaterials.2011.04.003

[249] Gonzalez-Molina J, Zhang X, Borghesan M, Mendonça da Silva J, Awan M, Fuller B, et al. Extracellular fluid viscosity enhances liver cancer cell mechanosensing and migration. Biomaterials. 2018;177:113–24. 29886384 10.1016/j.biomaterials.2018.05.058

[250] Elosegui-Artola A, Gupta A, Najibi AJ, Seo BR, Garry R, Tringides CM, et al. Matrix viscoelasticity controls spatiotemporal tissue organization. Nat Mater. 2023;22(1):117–27. 36456871 10.1038/s41563-022-01400-4 PMC10332325

[251] Yanez LZ, Han J, Behr BB, Pera RAR, Camarillo DB. Human oocyte developmental potential is predicted by mechanical properties within hours after fertilization. Nat Commun. 2016;7:10809. 26904963 10.1038/ncomms10809 PMC4770082

[252] Richardson BM, Walker CJ, Maples MM, Randolph MA, Bryant SJ, Anseth KS. Mechanobiological interactions between dynamic compressive loading and viscoelasticity on chondrocytes in Hydrazone Covalent Adaptable Networks for Cartilage tissue Engineering. Adv Healthc Mater. 2021;10(9):e2002030. 33738966 10.1002/adhm.202002030 PMC8785214

[253] Lee HP, Stowers R, Chaudhuri O. Volume expansion and TRPV4 activation regulate stem cell fate in three-dimensional microenvironments. Nat Commun. 2019;10(1):529. 30705265 10.1038/s41467-019-08465-x PMC6355972

[254] Murphy JG, Rajagopal KR. The residually stressed unloaded state of arteries: membrane and thin cylinder approximations. J Mech Behav Biomed Mater. 2021;122:104521. 34293693 10.1016/j.jmbbm.2021.104521

[255] Murphy JG, Rajagopal KR. Inflation of residually stressed Fung-type membrane models of arteries. J Mech Behav Biomed Mater. 2021;122:104699. 34332451 10.1016/j.jmbbm.2021.104699

[256] Melnikov A, Merodio J, Bustamante R, Dorfmann L. Bifurcation analysis of residually stressed neo-hookean and Ogden electroelastic tubes. Philos Trans Math Phys Eng Sci. 2022;380(2234):20210331. 10.1098/rsta.2021.0331 36031836

[257] Pashneh-Tala S, MacNeil S, Claeyssens F. The tissue-Engineered Vascular Graft-Past, Present, and Future. Tissue Eng Part B Rev. 2016;22(1):68–100. 26447530 10.1089/ten.teb.2015.0100 PMC4753638

[258] Wang J, Kong L, Gafur A, Peng X, Kristi N, Xu J, et al. Photooxidation crosslinking to recover residual stress in decellularized blood vessel. Regen Biomater. 2021;8(2):rbaa058. 33738112 10.1093/rb/rbaa058 PMC7955719

[259] Guccione JM, Moonly SM, Wallace AW, Ratcliffe MB. Residual stress produced by ventricular volume reduction surgery has little effect on ventricular function and mechanics: a finite element model study. J Thorac Cardiovasc Surg. 2001;122(3):592–9. 11547315 10.1067/mtc.2001.114939

[260] Zhao JB, Sha H, Zhuang FY, Gregersen H. Morphological properties and residual strain along the small intestine in rats. World J Gastroenterol. 2002;8(2):312–7. 11925615 10.3748/wjg.v8.i2.312 PMC4658374

[261] Hansen I, Gregersen H. Morphometry and residual strain in porcine ureter. Scand J Urol Nephrol. 1999;33(1):10–6. 10100357 10.1080/003655999750016203

[262] Gregersen H, Lee TC, Chien S, Skalak R, Fung YC. Strain distribution in the layered wall of the esophagus. J Biomech Eng. 1999;121(5):442–8. 10529910 10.1115/1.2835072

[263] Chaudhry HR, Bukiet B, Findley T, Ritter AB. Evaluation of residual stress in rabbit skin and the relevant material constants. J Theor Biol. 1998;192(2):191–5. 9637057 10.1006/jtbi.1997.0616

[264] Xu G, Bayly PV, Taber LA. Residual stress in the adult mouse brain. Biomech Model Mechanobiol. 2009;8(4):253–62. 18651186 10.1007/s10237-008-0131-4 PMC4605564

[265] Han HC, Fung YC. Residual strains in porcine and canine trachea. J Biomech. 1991;24(5):307–15. 2050707 10.1016/0021-9290(91)90349-r

[266] Yang Y, Paivinen P, Xie C, Krup AL, Makela TP, Mostov KE, et al. Ciliary hedgehog signaling patterns the digestive system to generate mechanical forces driving elongation. Nat Commun. 2021;12(1):7186. 34893605 10.1038/s41467-021-27319-z PMC8664829

[267] Zheng S, Banerji R, LeBourdais R, Zhang S, DuBois E, O’Shea T, et al. Alteration of mechanical stresses in the murine brain by age and hemorrhagic stroke. PNAS Nexus. 2024;3(4):pgae141. 38659974 10.1093/pnasnexus/pgae141 PMC11042661

[268] Taber LA, Hu N, Pexieder T, Clark EB, Keller BB. Residual strain in the ventricle of the stage 16–24 chick embryo. Circ Res. 1993;72(2):455–62. 8418994 10.1161/01.res.72.2.455

[269] Cai G, Nguyen A, Bashirzadeh Y, Lin SS, Bi D, Liu AP. Compressive stress drives adhesion-dependent unjamming transitions in breast cancer cell migration. Front Cell Dev Biol. 2022;10:933042. 36268514 10.3389/fcell.2022.933042 PMC9577106

[270] Liu H, Huang Y, Yang Y, Han Y, Jia L, Li W. Compressive force-induced LincRNA-p21 inhibits mineralization of cementoblasts by impeding autophagy. Faseb j. 2022;36(1):e22120. 34958157 10.1096/fj.202101589R

[271] Wang H, Li T, Jiang Y, Chen S, Zou S, Bonewald LF, et al. Force-Loaded Cementocytes regulate Osteoclastogenesis via S1P/S1PR1/Rac1 Axis. J Dent Res. 2023;102(12):1376–86. 37735908 10.1177/00220345231195765

[272] Oshinowo O, Azer SS, Lin J, Lam WA. Why platelet mechanotransduction matters for hemostasis and thrombosis. J Thromb Haemost. 2023;21(9):2339–53. 37331517 10.1016/j.jtha.2023.06.010 PMC10529432

[273] Maître JL, Turlier H, Illukkumbura R, Eismann B, Niwayama R, Nédélec F, et al. Asymmetric division of contractile domains couples cell positioning and fate specification. Nature. 2016;536(7616):344–8. 27487217 10.1038/nature18958 PMC4998956

[274] Cho S, Vashisth M, Abbas A, Majkut S, Vogel K, Xia Y, et al. Mechanosensing by the Lamina protects against Nuclear rupture, DNA damage, and cell-cycle arrest. Dev Cell. 2019;49(6):920–e355. 31105008 10.1016/j.devcel.2019.04.020 PMC6581604

[275] Dupont S, Morsut L, Aragona M, Enzo E, Giulitti S, Cordenonsi M, et al. Role of YAP/TAZ in mechanotransduction. Nature. 2011;474(7350):179–83. 21654799 10.1038/nature10137

[276] Robertson IB, Rifkin DB. Regulation of the bioavailability of TGF-β and TGF-β-Related proteins. Cold Spring Harb Perspect Biol. 2016;8(6). 10.1101/cshperspect.a021907 PMC4888822 27252363

[277] Sleboda DA, Roberts TJ. Internal fluid pressure influences muscle contractile force. Proc Natl Acad Sci U S A. 2020;117(3):1772–8. 31879350 10.1073/pnas.1914433117 PMC6983394

[278] Seano G, Nia HT, Emblem KE, Datta M, Ren J, Krishnan S, et al. Solid stress in brain tumours causes neuronal loss and neurological dysfunction and can be reversed by lithium. Nat Biomed Eng. 2019;3(3):230–45. 30948807 10.1038/s41551-018-0334-7 PMC6452896

[279] Jain RK, Martin JD, Stylianopoulos T. The role of mechanical forces in tumor growth and therapy. Annu Rev Biomed Eng. 2014;16:321–46. 25014786 10.1146/annurev-bioeng-071813-105259 PMC4109025

[280] Heisenberg CP, Bellaïche Y. Forces in tissue morphogenesis and patterning. Cell. 2013;153(5):948–62. 23706734 10.1016/j.cell.2013.05.008

[281] Voutouri C, Polydorou C, Papageorgis P, Gkretsi V, Stylianopoulos T. Hyaluronan-Derived swelling of Solid Tumors, the contribution of collagen and Cancer cells, and implications for Cancer Therapy. Neoplasia. 2016;18(12):732–41. 27886639 10.1016/j.neo.2016.10.001 PMC5122704

[282] Chauhan VP, Martin JD, Liu H, Lacorre DA, Jain SR, Kozin SV, et al. Angiotensin inhibition enhances drug delivery and potentiates chemotherapy by decompressing tumour blood vessels. Nat Commun. 2013;4:2516. 24084631 10.1038/ncomms3516 PMC3806395

[283] Simon DD, Horgan CO, Humphrey JD. Mechanical restrictions on biological responses by adherent cells within collagen gels. J Mech Behav Biomed Mater. 2012;14:216–26. 23022259 10.1016/j.jmbbm.2012.05.009 PMC3516288

[284] Tsoumakidou M. The advent of immune stimulating CAFs in cancer. Nat Rev Cancer. 2023;23(4):258–69. 36807417 10.1038/s41568-023-00549-7

[285] Stylianopoulos T, Munn LL, Jain RK. Reengineering the Tumor vasculature: improving Drug Delivery and Efficacy. Trends Cancer. 2018;4(4):258–9. 29606306 10.1016/j.trecan.2018.02.010 PMC6161778

[286] Griffon-Etienne G, Boucher Y, Brekken C, Suit HD, Jain RK. Taxane-induced apoptosis decompresses blood vessels and lowers interstitial fluid pressure in solid tumors: clinical implications. Cancer Res. 1999;59(15):3776–82. 10446995

[287] Padera TP, Stoll BR, Tooredman JB, Capen D, di Tomaso E, Jain RK. Pathology: cancer cells compress intratumour vessels. Nature. 2004;427(6976):695. 14973470 10.1038/427695a

[288] Tse JM, Cheng G, Tyrrell JA, Wilcox-Adelman SA, Boucher Y, Jain RK, et al. Mechanical compression drives cancer cells toward invasive phenotype. Proc Natl Acad Sci U S A. 2012;109(3):911–6. 22203958 10.1073/pnas.1118910109 PMC3271885

[289] Kirby TJ, Lammerding J. Emerging views of the nucleus as a cellular mechanosensor. Nat Cell Biol. 2018;20(4):373–81. 29467443 10.1038/s41556-018-0038-y PMC6440800

[290] Elosegui-Artola A, Andreu I, Beedle AEM, Lezamiz A, Uroz M, Kosmalska AJ, et al. Force triggers YAP Nuclear Entry by regulating transport across Nuclear pores. Cell. 2017;171(6):1397–e41014. 29107331 10.1016/j.cell.2017.10.008

[291] Li Y, Zhong Z, Xu C, Wu X, Li J, Tao W, et al. 3D micropattern force triggers YAP nuclear entry by transport across nuclear pores and modulates stem cells paracrine. Natl Sci Rev. 2023;10(8):nwad165. 37457331 10.1093/nsr/nwad165 PMC10347367

[292] Tajik A, Zhang Y, Wei F, Sun J, Jia Q, Zhou W, et al. Transcription upregulation via force-induced direct stretching of chromatin. Nat Mater. 2016;15(12):1287–96. 27548707 10.1038/nmat4729 PMC5121013

[293] Kumar A, Mazzanti M, Mistrik M, Kosar M, Beznoussenko GV, Mironov AA, et al. ATR mediates a checkpoint at the nuclear envelope in response to mechanical stress. Cell. 2014;158(3):633–46. 25083873 10.1016/j.cell.2014.05.046 PMC4121522

[294] Zanconato F, Cordenonsi M, Piccolo S. YAP/TAZ at the roots of Cancer. Cancer Cell. 2016;29(6):783–803. 27300434 10.1016/j.ccell.2016.05.005 PMC6186419

[295] Piccolo S, Panciera T, Contessotto P, Cordenonsi M. YAP/TAZ as master regulators in cancer: modulation, function and therapeutic approaches. Nat Cancer. 2023;4(1):9–26. 36564601 10.1038/s43018-022-00473-z PMC7614914

[296] Rosenbluh J, Nijhawan D, Cox AG, Li X, Neal JT, Schafer EJ, et al. β-Catenin-driven cancers require a YAP1 transcriptional complex for survival and tumorigenesis. Cell. 2012;151(7):1457–73. 23245941 10.1016/j.cell.2012.11.026 PMC3530160

[297] Zanconato F, Forcato M, Battilana G, Azzolin L, Quaranta E, Bodega B, et al. Genome-wide association between YAP/TAZ/TEAD and AP-1 at enhancers drives oncogenic growth. Nat Cell Biol. 2015;17(9):1218–27. 26258633 10.1038/ncb3216 PMC6186417

[298] Kapoor A, Yao W, Ying H, Hua S, Liewen A, Wang Q, et al. Yap1 activation enables bypass of oncogenic Kras addiction in pancreatic cancer. Cell. 2014;158(1):185–97. 24954535 10.1016/j.cell.2014.06.003 PMC4109295

[299] Barbazan J, Pérez-González C, Gómez-González M, Dedenon M, Richon S, Latorre E, et al. Cancer-associated fibroblasts actively compress cancer cells and modulate mechanotransduction. Nat Commun. 2023;14(1):6966. 37907483 10.1038/s41467-023-42382-4 PMC10618488

[300] Kubow KE, Vukmirovic R, Zhe L, Klotzsch E, Smith ML, Gourdon D, et al. Mechanical forces regulate the interactions of fibronectin and collagen I in extracellular matrix. Nat Commun. 2015;6:8026. 26272817 10.1038/ncomms9026 PMC4539566

[301] Saini K, Cho S, Dooling LJ, Discher DE. Tension in fibrils suppresses their enzymatic degradation - A molecular mechanism for ‘use it or lose it’. Matrix Biol. 2020;85–86:34–46. 31201857 10.1016/j.matbio.2019.06.001 PMC6906264

[302] Smith ML, Gourdon D, Little WC, Kubow KE, Eguiluz RA, Luna-Morris S, et al. Force-induced unfolding of fibronectin in the extracellular matrix of living cells. PLoS Biol. 2007;5(10):e268. 17914904 10.1371/journal.pbio.0050268 PMC1994993

[303] Li Y, Mao AS, Seo BR, Zhao X, Gupta SK, Chen M, et al. Compression-induced dedifferentiation of adipocytes promotes tumor progression. Sci Adv. 2020;6(4):eaax5611. 32010780 10.1126/sciadv.aax5611 PMC6976290

[304] Fernández-Sánchez ME, Barbier S, Whitehead J, Béalle G, Michel A, Latorre-Ossa H, et al. Mechanical induction of the tumorigenic β-catenin pathway by tumour growth pressure. Nature. 2015;523(7558):92–5. 25970250 10.1038/nature14329

[305] Wipff PJ, Rifkin DB, Meister JJ, Hinz B. Myofibroblast contraction activates latent TGF-beta1 from the extracellular matrix. J Cell Biol. 2007;179(6):1311–23. 18086923 10.1083/jcb.200704042 PMC2140013

[306] Rachidi S, Metelli A, Riesenberg B, Wu BX, Nelson MH, Wallace C et al. Platelets subvert T cell immunity against cancer via GARP-TGFβ axis. Sci Immunol. 2017;2(11). 10.1126/sciimmunol.aai7911 PMC5539882 28763790

[307] van Neerven SM, Vermeulen L. Cell competition in development, homeostasis and cancer. Nat Rev Mol Cell Biol. 2023;24(3):221–36. 36175766 10.1038/s41580-022-00538-y

[308] Merino MM, Levayer R, Moreno E. Survival of the Fittest: essential roles of cell competition in Development, Aging, and Cancer. Trends Cell Biol. 2016;26(10):776–88. 27319281 10.1016/j.tcb.2016.05.009

[309] Vishwakarma M, Piddini E. Outcompeting cancer. Nat Rev Cancer. 2020;20(3):187–98. 31932757 10.1038/s41568-019-0231-8

[310] Liu Z, Yee PP, Wei Y, Liu Z, Kawasawa YI, Li W. Differential YAP expression in glioma cells induces cell competition and promotes tumorigenesis. J Cell Sci. 2019;132(5). 10.1242/jcs.225714 PMC6432718 30665893

[311] Levayer R. Solid stress, competition for space and cancer: the opposing roles of mechanical cell competition in tumour initiation and growth. Semin Cancer Biol. 2020;63:69–80. 31077845 10.1016/j.semcancer.2019.05.004 PMC7221353

[312] Shraiman BI. Mechanical feedback as a possible regulator of tissue growth. Proc Natl Acad Sci U S A. 2005;102(9):3318–23. 15728365 10.1073/pnas.0404782102 PMC552900

[313] Murphy JE, Wo JY-L, Ferrone C, Jiang W, Yeap BY, Blaszkowsky LS, et al. TGF-B1 inhibition with losartan in combination with FOLFIRINOX (F-NOX) in locally advanced pancreatic cancer (LAPC): preliminary feasibility and R0 resection rates from a prospective phase II study. J Clin Oncol. 2017;35(4suppl):386.

[314] Murphy JE, Wo JY, Ryan DP, Clark JW, Jiang W, Yeap BY, et al. Total neoadjuvant therapy with FOLFIRINOX in Combination with Losartan followed by Chemoradiotherapy for locally advanced pancreatic Cancer: a phase 2 clinical trial. JAMA Oncol. 2019;5(7):1020–7. 31145418 10.1001/jamaoncol.2019.0892 PMC6547247

[315] Provenzano PP, Cuevas C, Chang AE, Goel VK, Von Hoff DD, Hingorani SR. Enzymatic targeting of the stroma ablates physical barriers to treatment of pancreatic ductal adenocarcinoma. Cancer Cell. 2012;21(3):418–29. 22439937 10.1016/j.ccr.2012.01.007 PMC3371414

[316] Sherman MH, Yu RT, Engle DD, Ding N, Atkins AR, Tiriac H, et al. Vitamin D receptor-mediated stromal reprogramming suppresses pancreatitis and enhances pancreatic cancer therapy. Cell. 2014;159(1):80–93. 25259922 10.1016/j.cell.2014.08.007 PMC4177038

[317] Kong W, Liu Z, Sun M, Liu H, Kong C, Ma J, et al. Synergistic autophagy blockade and VDR signaling activation enhance stellate cell reprogramming in pancreatic ductal adenocarcinoma. Cancer Lett. 2022;539:215718. 35526650 10.1016/j.canlet.2022.215718

[318] Olive KP, Jacobetz MA, Davidson CJ, Gopinathan A, McIntyre D, Honess D, et al. Inhibition of hedgehog signaling enhances delivery of chemotherapy in a mouse model of pancreatic cancer. Science. 2009;324(5933):1457–61. 19460966 10.1126/science.1171362 PMC2998180

[319] Chen IX, Chauhan VP, Posada J, Ng MR, Wu MW, Adstamongkonkul P, et al. Blocking CXCR4 alleviates desmoplasia, increases T-lymphocyte infiltration, and improves immunotherapy in metastatic breast cancer. Proc Natl Acad Sci U S A. 2019;116(10):4558–66. 30700545 10.1073/pnas.1815515116 PMC6410779

[320] Wang-Gillam A, Lim KH, McWilliams R, Suresh R, Lockhart AC, Brown A, et al. Defactinib, Pembrolizumab, and Gemcitabine in patients with Advanced Treatment Refractory Pancreatic Cancer: a phase I dose escalation and expansion study. Clin Cancer Res. 2022;28(24):5254–62. 36228156 10.1158/1078-0432.CCR-22-0308 PMC9772237

[321] Claesson-Welsh L, Dejana E, McDonald DM. Permeability of the endothelial barrier: identifying and reconciling controversies. Trends Mol Med. 2021;27(4):314–31. 33309601 10.1016/j.molmed.2020.11.006 PMC8005435

[322] Matsumoto Y, Nichols JW, Toh K, Nomoto T, Cabral H, Miura Y, et al. Vascular bursts enhance permeability of tumour blood vessels and improve nanoparticle delivery. Nat Nanotechnol. 2016;11(6):533–8. 26878143 10.1038/nnano.2015.342

[323] Carmeliet P, Jain RK. Angiogenesis in cancer and other diseases. Nature. 2000;407(6801):249–57. 11001068 10.1038/35025220

[324] Young JS, Lumsden CE, Stalker AL. The significance of the tissue pressure of normal testicular and of neoplastic (Brown-Pearce carcinoma) tissue in the rabbit. J Pathol Bacteriol. 1950;62(3):313–33. 14784896 10.1002/path.1700620303

[325] Pedersen JA, Lichter S, Swartz MA. Cells in 3D matrices under interstitial flow: effects of extracellular matrix alignment on cell shear stress and drag forces. J Biomech. 2010;43(5):900–5. 20006339 10.1016/j.jbiomech.2009.11.007

[326] Chauhan VP, Boucher Y, Ferrone CR, Roberge S, Martin JD, Stylianopoulos T, et al. Compression of pancreatic tumor blood vessels by hyaluronan is caused by solid stress and not interstitial fluid pressure. Cancer Cell. 2014;26(1):14–5. 25026209 10.1016/j.ccr.2014.06.003 PMC4381566

[327] Less JR, Posner MC, Boucher Y, Borochovitz D, Wolmark N, Jain RK. Interstitial hypertension in human breast and colorectal tumors. Cancer Res. 1992;52(22):6371–4. 1423283

[328] Roh HD, Boucher Y, Kalnicki S, Buchsbaum R, Bloomer WD, Jain RK. Interstitial hypertension in carcinoma of uterine cervix in patients: possible correlation with tumor oxygenation and radiation response. Cancer Res. 1991;51(24):6695–8. 1742744

[329] Gutmann R, Leunig M, Feyh J, Goetz AE, Messmer K, Kastenbauer E, et al. Interstitial hypertension in head and neck tumors in patients: correlation with tumor size. Cancer Res. 1992;52(7):1993–5. 1551128

[330] Curti BD, Urba WJ, Alvord WG, Janik JE, Smith JW 2nd, Madara K, et al. Interstitial pressure of subcutaneous nodules in melanoma and lymphoma patients: changes during treatment. Cancer Res. 1993;53(10 Suppl):2204–7. 8485703

[331] Boucher Y, Kirkwood JM, Opacic D, Desantis M, Jain RK. Interstitial hypertension in superficial metastatic melanomas in humans. Cancer Res. 1991;51(24):6691–4. 1742743

[332] Jain RK, Baxter LT. Mechanisms of heterogeneous distribution of monoclonal antibodies and other macromolecules in tumors: significance of elevated interstitial pressure. Cancer Res. 1988;48(24 Pt 1):7022–32. 3191477

[333] Jasuja H, Jaswandkar SV, Katti DR, Katti KS. Interstitial fluid flow contributes to prostate cancer invasion and migration to bone; study conducted using a novel horizontal flow bioreactor. Biofabrication. 2023;15(2). 10.1088/1758-5090/acc09a PMC10020972 36863017

[334] Polacheck WJ, Charest JL, Kamm RD. Interstitial flow influences direction of tumor cell migration through competing mechanisms. Proc Natl Acad Sci U S A. 2011;108(27):11115–20. 21690404 10.1073/pnas.1103581108 PMC3131352

[335] Shieh AC, Rozansky HA, Hinz B, Swartz MA. Tumor cell invasion is promoted by interstitial flow-induced matrix priming by stromal fibroblasts. Cancer Res. 2011;71(3):790–800. 21245098 10.1158/0008-5472.CAN-10-1513

[336] Lawler K, O’Sullivan G, Long A, Kenny D. Shear stress induces internalization of E-cadherin and invasiveness in metastatic oesophageal cancer cells by a src-dependent pathway. Cancer Sci. 2009;100(6):1082–7. 19432901 10.1111/j.1349-7006.2009.01160.x PMC11159203

[337] Leckband DE, de Rooij J. Cadherin adhesion and mechanotransduction. Annu Rev Cell Dev Biol. 2014;30:291–315. 25062360 10.1146/annurev-cellbio-100913-013212

[338] Labernadie A, Kato T, Brugués A, Serra-Picamal X, Derzsi S, Arwert E, et al. A mechanically active heterotypic E-cadherin/N-cadherin adhesion enables fibroblasts to drive cancer cell invasion. Nat Cell Biol. 2017;19(3):224–37. 28218910 10.1038/ncb3478 PMC5831988

[339] Moran H, Cancel LM, Mayer MA, Qazi H, Munn LL, Tarbell JM. The cancer cell glycocalyx proteoglycan Glypican-1 mediates interstitial flow mechanotransduction to enhance cell migration and metastasis. Biorheology. 2019;56(2–3):151–61. 31256115 10.3233/BIR-180203

[340] Tarbell JM, Cancel LM. The glycocalyx and its significance in human medicine. J Intern Med. 2016;280(1):97–113. 26749537 10.1111/joim.12465

[341] Swartz MA, Lund AW. Lymphatic and interstitial flow in the tumour microenvironment: linking mechanobiology with immunity. Nat Rev Cancer. 2012;12(3):210–9. 22362216 10.1038/nrc3186

[342] Ghiringhelli F, Puig PE, Roux S, Parcellier A, Schmitt E, Solary E, et al. Tumor cells convert immature myeloid dendritic cells into TGF-beta-secreting cells inducing CD4 + CD25 + regulatory T cell proliferation. J Exp Med. 2005;202(7):919–29. 16186184 10.1084/jem.20050463 PMC2213166

[343] Lou C, Wu K, Shi J, Dai Z, Xu Q. N-cadherin protects oral cancer cells from NK cell killing in the circulation by inducing NK cell functional exhaustion via the KLRG1 receptor. J Immunother Cancer. 2022;10(9). 10.1136/jitc-2022-005061 PMC9472211 36096526

[344] Sun J, Luo Q, Liu L, Song G. Low-level shear stress promotes migration of liver cancer stem cells via the FAK-ERK1/2 signalling pathway. Cancer Lett. 2018;427:1–8. 29678550 10.1016/j.canlet.2018.04.015

[345] Kim OH, Choi YW, Park JH, Hong SA, Hong M, Chang IH, et al. Fluid shear stress facilitates prostate cancer metastasis through Piezo1-Src-YAP axis. Life Sci. 2022;308:120936. 36084759 10.1016/j.lfs.2022.120936

[346] Qazi H, Shi ZD, Tarbell JM. Fluid shear stress regulates the invasive potential of glioma cells via modulation of migratory activity and matrix metalloproteinase expression. PLoS ONE. 2011;6(5):e20348. 21637818 10.1371/journal.pone.0020348 PMC3102715

[347] Chang SF, Chang CA, Lee DY, Lee PL, Yeh YM, Yeh CR, et al. Tumor cell cycle arrest induced by shear stress: roles of integrins and smad. Proc Natl Acad Sci U S A. 2008;105(10):3927–32. 18310319 10.1073/pnas.0712353105 PMC2268796

[348] Lee YH, Lai CW, Cheng YC. Fluid shear stress induces cell cycle arrest in human urinary bladder transitional cell Carcinoma through Bone morphogenetic protein Receptor-Smad1/5 pathway. Cell Mol Bioeng. 2018;11(3):185–95. 31719885 10.1007/s12195-018-0523-1 PMC6816781

[349] Song JW, Munn LL. Fluid forces control endothelial sprouting. Proc Natl Acad Sci U S A. 2011;108(37):15342–7. 21876168 10.1073/pnas.1105316108 PMC3174629

[350] Galie PA, Nguyen DH, Choi CK, Cohen DM, Janmey PA, Chen CS. Fluid shear stress threshold regulates angiogenic sprouting. Proc Natl Acad Sci U S A. 2014;111(22):7968–73. 24843171 10.1073/pnas.1310842111 PMC4050561

[351] Boucher Y, Leunig M, Jain RK. Tumor angiogenesis and interstitial hypertension. Cancer Res. 1996;56(18):4264–6. 8797602

[352] Li J, Hou B, Tumova S, Muraki K, Bruns A, Ludlow MJ, et al. Piezo1 integration of vascular architecture with physiological force. Nature. 2014;515(7526):279–82. 25119035 10.1038/nature13701 PMC4230887

[353] Polacheck WJ, Kutys ML, Yang J, Eyckmans J, Wu Y, Vasavada H, et al. A non-canonical notch complex regulates adherens junctions and vascular barrier function. Nature. 2017;552(7684):258–62. 29160307 10.1038/nature24998 PMC5730479

[354] Wang KC, Yeh YT, Nguyen P, Limqueco E, Lopez J, Thorossian S, et al. Flow-dependent YAP/TAZ activities regulate endothelial phenotypes and atherosclerosis. Proc Natl Acad Sci U S A. 2016;113(41):11525–30. 27671657 10.1073/pnas.1613121113 PMC5068257

[355] Wang L, Luo JY, Li B, Tian XY, Chen LJ, Huang Y, et al. Integrin-YAP/TAZ-JNK cascade mediates atheroprotective effect of unidirectional shear flow. Nature. 2016;540(7634):579–82. 27926730 10.1038/nature20602

[356] Hubert P, Roncarati P, Demoulin S, Pilard C, Ancion M, Reynders C et al. Extracellular HMGB1 blockade inhibits tumor growth through profoundly remodeling immune microenvironment and enhances checkpoint inhibitor-based immunotherapy. J Immunother Cancer. 2021;9(3). 10.1136/jitc-2020-001966 PMC7959241 33712445

[357] Shang M, Lim SB, Jiang K, Yap YS, Khoo BL, Han J, et al. Microfluidic studies of hydrostatic pressure-enhanced doxorubicin resistance in human breast cancer cells. Lab Chip. 2021;21(4):746–54. 33502419 10.1039/d0lc01103g

[358] De Palma M, Biziato D, Petrova TV. Microenvironmental regulation of tumour angiogenesis. Nat Rev Cancer. 2017;17(8):457–74. 28706266 10.1038/nrc.2017.51

[359] Liu ZL, Chen HH, Zheng LL, Sun LP, Shi L. Angiogenic signaling pathways and anti-angiogenic therapy for cancer. Signal Transduct Target Ther. 2023;8(1):198. 37169756 10.1038/s41392-023-01460-1 PMC10175505

[360] Kloepper J, Riedemann L, Amoozgar Z, Seano G, Susek K, Yu V, et al. Ang-2/VEGF bispecific antibody reprograms macrophages and resident microglia to anti-tumor phenotype and prolongs glioblastoma survival. Proc Natl Acad Sci U S A. 2016;113(16):4476–81. 27044098 10.1073/pnas.1525360113 PMC4843473

[361] Schmittnaegel M, Rigamonti N, Kadioglu E, Cassará A, Wyser Rmili C, Kiialainen A et al. Dual angiopoietin-2 and VEGFA inhibition elicits antitumor immunity that is enhanced by PD-1 checkpoint blockade. Sci Transl Med. 2017;9(385). 10.1126/scitranslmed.aak9670 28404865

[362] Lee WS, Yang H, Chon HJ, Kim C. Combination of anti-angiogenic therapy and immune checkpoint blockade normalizes vascular-immune crosstalk to potentiate cancer immunity. Exp Mol Med. 2020;52(9):1475–85. 32913278 10.1038/s12276-020-00500-y PMC8080646

[363] Fukumura D, Kloepper J, Amoozgar Z, Duda DG, Jain RK. Enhancing cancer immunotherapy using antiangiogenics: opportunities and challenges. Nat Rev Clin Oncol. 2018;15(5):325–40. 29508855 10.1038/nrclinonc.2018.29 PMC5921900

[364] Jiang X, Xu S, Miao Y, Huang K, Wang B, Ding B, et al. Curvature-mediated rapid extravasation and penetration of nanoparticles against interstitial fluid pressure for improved drug delivery. Proc Natl Acad Sci U S A. 2024;121(22):e2319880121. 38768353 10.1073/pnas.2319880121 PMC11145294

[365] Hectors SJ, Lewis S. Tomoelastography of the prostate: use of tissue stiffness for Improved Cancer Detection. Radiology. 2021;299(2):371–3. 33689473 10.1148/radiol.2021210292

[366] Tarchi SM, Pernia Marin M, Hossain MM, Salvatore M. Breast stiffness, a risk factor for cancer and the role of radiology for diagnosis. J Transl Med. 2023;21(1):582. 37649088 10.1186/s12967-023-04457-0 PMC10466778

[367] Northey JJ, Hayward MK, Yui Y, Stashko C, Kai F, Mouw JK, et al. Mechanosensitive hormone signaling promotes mammary progenitor expansion and breast cancer risk. Cell Stem Cell. 2024;31(1):106–e2613. 38181747 10.1016/j.stem.2023.12.002 PMC11050720

[368] Di Chiaro P, Nacci L, Arco F, Brandini S, Polletti S, Palamidessi A, et al. Mapping functional to morphological variation reveals the basis of regional extracellular matrix subversion and nerve invasion in pancreatic cancer. Cancer Cell. 2024;42(4):662–e8110. 38518775 10.1016/j.ccell.2024.02.017

[369] Shen Y, Wang X, Lu J, Salfenmoser M, Wirsik NM, Schleussner N, et al. Reduction of liver metastasis stiffness improves response to Bevacizumab in Metastatic Colorectal Cancer. Cancer Cell. 2020;37(6):800–e177. 32516590 10.1016/j.ccell.2020.05.005

[370] Lopez-Cavestany M, Hahn SB, Hope JM, Reckhorn NT, Greenlee JD, Schwager SC, et al. Matrix stiffness induces epithelial-to-mesenchymal transition via Piezo1-regulated calcium flux in prostate cancer cells. iScience. 2023;26(4):106275. 36950111 10.1016/j.isci.2023.106275 PMC10025097

[371] Storm C, Pastore JJ, MacKintosh FC, Lubensky TC, Janmey PA. Nonlinear elasticity in biological gels. Nature. 2005;435(7039):191–4. 15889088 10.1038/nature03521

[372] Han YL, Ronceray P, Xu G, Malandrino A, Kamm RD, Lenz M, et al. Cell contraction induces long-ranged stress stiffening in the extracellular matrix. Proc Natl Acad Sci U S A. 2018;115(16):4075–80. 29618614 10.1073/pnas.1722619115 PMC5910866

[373] Szulczewski JM, Inman DR, Proestaki M, Notbohm J, Burkel BM, Ponik SM. Directional cues in the tumor microenvironment due to cell contraction against aligned collagen fibers. Acta Biomater. 2021;129:96–109. 33965625 10.1016/j.actbio.2021.04.053 PMC8848478

[374] Guilluy C, Osborne LD, Van Landeghem L, Sharek L, Superfine R, Garcia-Mata R, et al. Isolated nuclei adapt to force and reveal a mechanotransduction pathway in the nucleus. Nat Cell Biol. 2014;16(4):376–81. 24609268 10.1038/ncb2927 PMC4085695

[375] Filliol A, Saito Y, Nair A, Dapito DH, Yu LX, Ravichandra A, et al. Opposing roles of hepatic stellate cell subpopulations in hepatocarcinogenesis. Nature. 2022;610(7931):356–65. 36198802 10.1038/s41586-022-05289-6 PMC9949942

[376] Bao M, Chen Y, Liu JT, Bao H, Wang WB, Qi YX, et al. Extracellular matrix stiffness controls VEGF(165) secretion and neuroblastoma angiogenesis via the YAP/RUNX2/SRSF1 axis. Angiogenesis. 2022;25(1):71–86. 34170441 10.1007/s10456-021-09804-7

[377] Torrino S, Grasset EM, Audebert S, Belhadj I, Lacoux C, Haynes M, et al. Mechano-induced cell metabolism promotes microtubule glutamylation to force metastasis. Cell Metab. 2021;33(7):1342–e5710. 34102109 10.1016/j.cmet.2021.05.009

[378] Bertero T, Oldham WM, Grasset EM, Bourget I, Boulter E, Pisano S, et al. Tumor-stroma mechanics coordinate amino acid availability to sustain Tumor Growth and Malignancy. Cell Metab. 2019;29(1):124–e4010. 30293773 10.1016/j.cmet.2018.09.012 PMC6432652

[379] Sohrabi A, Lefebvre A, Harrison MJ, Condro MC, Sanazzaro TM, Safarians G, et al. Microenvironmental stiffness induces metabolic reprogramming in glioblastoma. Cell Rep. 2023;42(10):113175. 37756163 10.1016/j.celrep.2023.113175 PMC10842372

[380] Miyazawa A, Ito S, Asano S, Tanaka I, Sato M, Kondo M, et al. Regulation of PD-L1 expression by matrix stiffness in lung cancer cells. Biochem Biophys Res Commun. 2018;495(3):2344–9. 29274784 10.1016/j.bbrc.2017.12.115

[381] Li CX, Talele NP, Boo S, Koehler A, Knee-Walden E, Balestrini JL, et al. MicroRNA-21 preserves the fibrotic mechanical memory of mesenchymal stem cells. Nat Mater. 2017;16(3):379–89. 27798620 10.1038/nmat4780

[382] Winkler J, Abisoye-Ogunniyan A, Metcalf KJ, Werb Z. Concepts of extracellular matrix remodelling in tumour progression and metastasis. Nat Commun. 2020;11(1):5120. 33037194 10.1038/s41467-020-18794-x PMC7547708

[383] Kalluri R. The biology and function of fibroblasts in cancer. Nat Rev Cancer. 2016;16(9):582–98. 27550820 10.1038/nrc.2016.73

[384] Rimal R, Desai P, Daware R, Hosseinnejad A, Prakash J, Lammers T, et al. Cancer-associated fibroblasts: origin, function, imaging, and therapeutic targeting. Adv Drug Deliv Rev. 2022;189:114504. 35998825 10.1016/j.addr.2022.114504

[385] Peng D, Fu M, Wang M, Wei Y, Wei X. Targeting TGF-β signal transduction for fibrosis and cancer therapy. Mol Cancer. 2022;21(1):104. 35461253 10.1186/s12943-022-01569-x PMC9033932

[386] Bhattacharjee S, Hamberger F, Ravichandra A, Miller M, Nair A, Affo S et al. Tumor restriction by type I collagen opposes tumor-promoting effects of cancer-associated fibroblasts. J Clin Invest. 2021;131(11). 10.1172/JCI146987 PMC8159701 33905375

[387] Schrader J, Gordon-Walker TT, Aucott RL, van Deemter M, Quaas A, Walsh S, et al. Matrix stiffness modulates proliferation, chemotherapeutic response, and dormancy in hepatocellular carcinoma cells. Hepatology. 2011;53(4):1192–205. 21442631 10.1002/hep.24108 PMC3076070

[388] Dou C, Liu Z, Tu K, Zhang H, Chen C, Yaqoob U, et al. P300 Acetyltransferase mediates Stiffness-Induced activation of hepatic stellate cells into tumor-promoting myofibroblasts. Gastroenterology. 2018;154(8):2209–e2114. 29454793 10.1053/j.gastro.2018.02.015 PMC6039101

[389] Xiong J, Xiao R, Zhao J, Zhao Q, Luo M, Li F, et al. Matrix stiffness affects tumor-associated macrophage functional polarization and its potential in tumor therapy. J Transl Med. 2024;22(1):85. 38246995 10.1186/s12967-023-04810-3 PMC10800063

[390] Chakraborty M, Chu K, Shrestha A, Revelo XS, Zhang X, Gold MJ, et al. Mechanical stiffness controls dendritic cell metabolism and function. Cell Rep. 2021;34(2):108609. 33440149 10.1016/j.celrep.2020.108609

[391] Wang Y, Yang H, Jia A, Wang Y, Yang Q, Dong Y et al. Dendritic cell Piezo1 directs the differentiation of T(H)1 and T(reg) cells in cancer. Elife. 2022;11. 10.7554/eLife.79957 PMC9451538 35993548

[392] Mennens SFB, Bolomini-Vittori M, Weiden J, Joosten B, Cambi A, van den Dries K. Substrate stiffness influences phenotype and function of human antigen-presenting dendritic cells. Sci Rep. 2017;7(1):17511. 29235514 10.1038/s41598-017-17787-z PMC5727489

[393] Zhang T, Jia Y, Yu Y, Zhang B, Xu F, Guo H. Targeting the tumor biophysical microenvironment to reduce resistance to immunotherapy. Adv Drug Deliv Rev. 2022;186:114319. 35545136 10.1016/j.addr.2022.114319

[394] Mordechay L, Le Saux G, Edri A, Hadad U, Porgador A, Schvartzman M. Mechanical regulation of the cytotoxic activity of natural killer cells. ACS Biomater Sci Eng. 2021;7(1):122–32. 33455204 10.1021/acsbiomaterials.0c01121

[395] Kaul A, Short WD, Wang X, Keswani SG. Hyaluronidases in Human diseases. Int J Mol Sci. 2021;22(6). 10.3390/ijms22063204 PMC8004219 33809827

[396] Loriot Y, Necchi A, Park SH, Garcia-Donas J, Huddart R, Burgess E, et al. Erdafitinib in locally Advanced or Metastatic Urothelial Carcinoma. N Engl J Med. 2019;381(4):338–48. 31340094 10.1056/NEJMoa1817323

[397] McAndrews KM, Vázquez-Arreguín K, Kwak C, Sugimoto H, Zheng X, Li B, et al. αSMA(+) fibroblasts suppress Lgr5(+) cancer stem cells and restrain colorectal cancer progression. Oncogene. 2021;40(26):4440–52. 34108617 10.1038/s41388-021-01866-7 PMC9150816

[398] Formenti SC, Lee P, Adams S, Goldberg JD, Li X, Xie MW, et al. Focal irradiation and systemic TGFβ blockade in metastatic breast Cancer. Clin Cancer Res. 2018;24(11):2493–504. 29476019 10.1158/1078-0432.CCR-17-3322 PMC5999326

[399] Melisi D, Garcia-Carbonero R, Macarulla T, Pezet D, Deplanque G, Fuchs M, et al. TGFβ receptor inhibitor galunisertib is linked to inflammation- and remodeling-related proteins in patients with pancreatic cancer. Cancer Chemother Pharmacol. 2019;83(5):975–91. 30887178 10.1007/s00280-019-03807-4

[400] Zhao Z, Yang W, Kong R, Zhang Y, Li L, Song Z, et al. circEIF3I facilitates the recruitment of SMAD3 to early endosomes to promote TGF-β signalling pathway-mediated activation of MMPs in pancreatic cancer. Mol Cancer. 2023;22(1):152. 37689715 10.1186/s12943-023-01847-2 PMC10492306

[401] Incio J, Suboj P, Chin SM, Vardam-Kaur T, Liu H, Hato T, et al. Metformin reduces Desmoplasia in Pancreatic Cancer by Reprogramming Stellate cells and Tumor-Associated macrophages. PLoS ONE. 2015;10(12):e0141392. 26641266 10.1371/journal.pone.0141392 PMC4671732

[402] Liu L, Zhang SX, Liao W, Farhoodi HP, Wong CW, Chen CC et al. Mechanoresponsive stem cells to target cancer metastases through biophysical cues. Sci Transl Med. 2017;9(400). 10.1126/scitranslmed.aan2966 PMC5890431 28747514

[403] Kufe DW. Mucins in cancer: function, prognosis and therapy. Nat Rev Cancer. 2009;9(12):874–85. 19935676 10.1038/nrc2761 PMC2951677

[404] Yin J, Kong X, Lin W. Noninvasive Cancer diagnosis in vivo based on a viscosity-activated Near-Infrared fluorescent probe. Anal Chem. 2021;93(4):2072–81. 33393756 10.1021/acs.analchem.0c03803

[405] Pittman M, Iu E, Li K, Wang M, Chen J, Taneja N, et al. Membrane ruffling is a Mechanosensor of Extracellular Fluid Viscosity. Nat Phys. 2022;18(9):1112–21. 37220497 10.1038/s41567-022-01676-y PMC10202009

[406] Adebowale K, Gong Z, Hou JC, Wisdom KM, Garbett D, Lee HP, et al. Enhanced substrate stress relaxation promotes filopodia-mediated cell migration. Nat Mater. 2021;20(9):1290–9. 33875851 10.1038/s41563-021-00981-w PMC8390443

[407] Nam S, Gupta VK, Lee HP, Lee JY, Wisdom KM, Varma S, et al. Cell cycle progression in confining microenvironments is regulated by a growth-responsive TRPV4-PI3K/Akt-p27(Kip1) signaling axis. Sci Adv. 2019;5(8):eaaw6171. 31457089 10.1126/sciadv.aaw6171 PMC6685709

[408] Fan W, Adebowale K, Vancza L, Li Y, Rabbi MF, Kunimoto K, et al. Matrix viscoelasticity promotes liver cancer progression in the pre-cirrhotic liver. Nature. 2024;626(7999):635–42. 38297127 10.1038/s41586-023-06991-9 PMC10866704

[409] Bera K, Kiepas A, Godet I, Li Y, Mehta P, Ifemembi B, et al. Extracellular fluid viscosity enhances cell migration and cancer dissemination. Nature. 2022;611(7935):365–73. 36323783 10.1038/s41586-022-05394-6 PMC9646524

[410] Cieśluk M, Piktel E, Wnorowska U, Skłodowski K, Kochanowicz J, Kułakowska A, et al. Substrate viscosity impairs temozolomide-mediated inhibition of glioblastoma cells’ growth. Biochim Biophys Acta Mol Basis Dis. 2022;1868(11):166513. 35932892 10.1016/j.bbadis.2022.166513

[411] Sinha S, Ayushman M, Tong X, Yang F. Dynamically crosslinked poly(ethylene-glycol) hydrogels reveal a critical role of viscoelasticity in modulating glioblastoma fates and drug responses in 3D. Adv Healthc Mater. 2023;12(1):e2202147. 36239185 10.1002/adhm.202202147 PMC9813196

[412] DelGiorno KE, Carlson MA, Osgood R, Provenzano PP, Brockenbough JS, Thompson CB, et al. Response to Chauhan et Al.: interstitial pressure and vascular collapse in pancreas cancer-fluids and solids, measurement and meaning. Cancer Cell. 2014;26(1):16–7. 25026210 10.1016/j.ccr.2014.06.004 PMC4465109

[413] Pagé G, Tardieu M, Gennisson JL, Besret L, Garteiser P, Van Beers BE. Tumor solid stress: Assessment with MR Elastography under Compression of patient-derived Hepatocellular Carcinomas and Cholangiocarcinomas Xenografted in mice. Cancers (Basel). 2021;13(8). 10.3390/cancers13081891 PMC8071192 33920771

[414] Scott EC, Baines AC, Gong Y, Moore R Jr., Pamuk GE, Saber H, et al. Trends in the approval of cancer therapies by the FDA in the twenty-first century. Nat Rev Drug Discov. 2023;22(8):625–40. 37344568 10.1038/s41573-023-00723-4

[415] Mei J, Chu J, Yang K, Luo Z, Yang J, Xu J et al. Angiotensin receptor blocker attacks armored and cold tumors and boosts immune checkpoint blockade. J Immunother Cancer. 2024;12(9). 10.1136/jitc-2024-009327 PMC11418576 39244215

[416] Lopez-Crapez E, Costa L, Tosato G, Ramos J, Mazard T, Guiramand J, et al. Mechanical signatures of human colon cancers. Sci Rep. 2022;12(1):12475. 35864200 10.1038/s41598-022-16669-3 PMC9304395

[417] Yu H, Zhu L, Wang Y, Yue X, Wang W, Sun Z, et al. Amide Proton transfer weighted MR Imaging for Predicting Meningioma stiffness: a feasibility study. J Magn Reson Imaging. 2023;57(4):1071–8. 35932167 10.1002/jmri.28379

